# PRMT1 mediated methylation of cGAS suppresses anti-tumor immunity

**DOI:** 10.1038/s41467-023-38443-3

**Published:** 2023-05-17

**Authors:** Jing Liu, Xia Bu, Chen Chu, Xiaoming Dai, John M. Asara, Piotr Sicinski, Gordon J. Freeman, Wenyi Wei

**Affiliations:** 1 Department of Pathology, Beth Israel Deaconess Medical Center, Harvard Medical School, Boston, MA 02215 USA; 2 Key Laboratory of Environment and Genes Related to Diseases, Ministry of Education, Xi’an, 710061 P.R. China; 3 Key Laboratory for Tumor Precision Medicine of Shaanxi Province, The First Affiliated Hospital of Xi’an Jiaotong University, Xi’an, 710061 P.R. China; 4 Department of Medical Oncology, Dana-Farber Cancer Institute, Harvard Medical School, Boston, MA 02215 USA; 5 Department of Cancer Biology, Dana-Farber Cancer Institute, Boston, MA 02215 USA; 6 Department of Genetics, Blavatnik Institute, Harvard Medical School, Boston, MA 02215 USA; 7 Mass Spectrometry Core, Beth Israel Deaconess Medical Center, Department of Medicine, Harvard Medical School, Boston, MA 02215 USA; 8 Department of Histology and Embryology, Center for Biostructure Research, Medical University of Warsaw, Warsaw, Poland; 9 Present Address: Department of Urology, The First Affiliated Hospital of Xi’an Jiaotong University, Xi’an, 710061 P.R. China

**Keywords:** Immunosurveillance, Cancer immunotherapy, Methylation, Pattern recognition receptors

## Abstract

Activation of the cGAS/STING innate immunity pathway is essential and effective for anti-tumor immunotherapy. However, it remains largely elusive how tumor-intrinsic cGAS signaling is suppressed to facilitate tumorigenesis by escaping immune surveillance. Here, we report that the protein arginine methyltransferase, PRMT1, methylates cGAS at the conserved Arg133 residue, which prevents cGAS dimerization and suppresses the cGAS/STING signaling in cancer cells. Notably, genetic or pharmaceutical ablation of *PRMT1* leads to activation of cGAS/STING-dependent DNA sensing signaling, and robustly elevates the transcription of type I and II interferon response genes. As such, PRMT1 inhibition elevates tumor-infiltrating lymphocytes in a cGAS-dependent manner, and promotes tumoral PD-L1 expression. Thus, combination therapy of PRMT1 inhibitor with anti-PD-1 antibody augments the anti-tumor therapeutic efficacy in vivo. Our study therefore defines the PRMT1/cGAS/PD-L1 regulatory axis as a critical factor in determining immune surveillance efficacy, which serves as a promising therapeutic target for boosting tumor immunity.

## Abstract (web summary)

cGAS/STING mediated immunity is linked to the anti-tumor response, but how tumor-intrinsic cGAS signals are countered during tumorigenesis and immune evasion is poorly understood. Here the authors show PRMT1 suppresses the anti-tumor immune response via arginine methylation of cGAS.

## Introduction

Innate immunity serves as the first defense mechanism against infective bacteria and viruses, among which cyclic GMP-AMP synthase (cGAS) is a major sensor for the presence of cytosolic DNA derived from bacterial or viral infection^1–3^. Upon stimulation by cytosolic DNA, cGAS converts ATP and GTP into 2′3′-cyclic GMP-AMP (cGAMP)^4–6^. As a second messenger, cGAMP binds with the stimulator of interferon genes (STING)^5,7^, triggers its translocation from the endoplasmic reticulum (ER) onto Golgi apparatus to server as a platform to recruit TANK-binding kinase 1 (TBK1) and IκB kinase (IKK) for the phosphorylation of the downstream effectors such as interferon regulatory factor 3 (IRF3) and NF-kappaB (NF-κB), which initiate the transcription of type I interferons (IFNs) and cytokines^8–10^.

The presence of cGAS/STING signaling in both tumor cells and immune-competent mice is essential for anti-tumor immunity, and depletion of cGAS and/or STING in either tumor cells or immune-competent mice robustly dampen the tumor immunogenicity and efficiency of immunotherapy^11,12^. On the other hand, administration of cGAMP or STING agonists synergize with immune checkpoint blockades (ICBs) in the syngeneic mouse models^11,13,14^. Moreover, therapies that disrupt DNA damage repair to elevate cytosolic DNA levels, such as PARP inhibitor, CHK1 inhibitor, and ATM inhibitor, synergize with anti-tumor immunotherapy in part through activating the cGAS/STING pathway^15–17^.

Notably, the posttranslational modification of cGAS, including ubiquitination, acetylation, and phosphorylation, has been reported to regulate its enzymatic activity in both immune cells and cancer cells^18–23^. However, it remains largely unknown how intra-tumoral cGAS/STING signaling is suppressed to facilitate the immune evasion of cancer cells. cGAS has a positive-charged lysine- and arginine-rich N-terminus, which is essential for cGAS phase transition and membrane translocation^24,25^. Arginine residue in protein undergoes methylation, which is catalyzed by protein arginine methyltransferase (PRMT)^26^. Three different types of arginine methylation exist for human protein, i.e., monomethylarginine (MMA), asymmetric dimethylarginine (ADMA), and symmetric dimethylarginine (SDMA)^26^. Protein arginine methylation plays an important function in negatively regulating antiviral responses, such as PRMT3-mediated RIG-1 methylation^27^, inhibition of IRF3 by PRMT6 independent of its enzyme acitivity^28^, PRMT7-mediated MAVS methylation^29^, PRMT5-mediated cGAS methylation^30,31^, although it is still controversial^32^. PRMT1 is the major type I PRMT that is responsible for over 90% of ADMA, a modification that frequently occurs in DNA- and RNA-binding proteins, such as the Histone and CHTOP^33–35^. However, it is still poorly understood whether and how PRMT1 regulates cGAS activity. Recently, PRMT1 inhibition has been reported to induce a viral mimicry response in human triple-negative breast cancer (TNBC) cells^36^, though whether and how it interferes cGAS/STING signaling remains elusive.

In this study, we provide evidence that the protein arginine methyltransferase, PRMT1, plays a critical role in suppressing cGAS/STING signaling through methylating cGAS at the conserved Arg133 residue on its N-terminus. Thus, PRMT1 is a potential target for cancer immunotherapy and PRMT1 inhibitor synergizes with immune checkpoint blockades to boost cancer immunity.

## Results

### PRMT1 methylates cGAS and suppresses cGAS/STING signaling in cancer cells

**Fig. 1:**
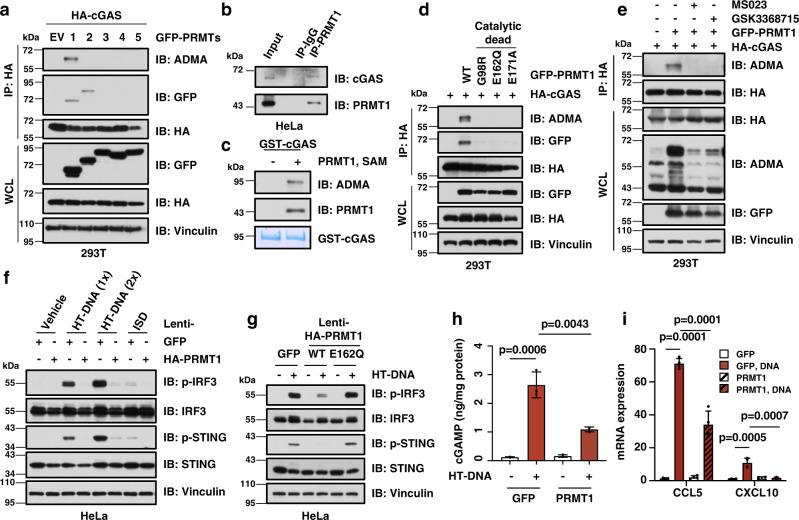
PRMT1 methylates cGAS and inhibits the cGAS/STING DNA sensing signaling. **a** PRMT1 interacts with and methylates cGAS. ADMA: asymmetric dimethylarginine. Immunoblot analysis of the HA immunoprecipitant and WCL derived from HEK293T cells that ectopically express HA-cGAS and GFP-PRMTs constructs. **b** Endogenous PRMT1 binds with cGAS. Immunoblot analysis of the immunoprecipitant derived from HeLa cells using either PRMT1 antibody or control IgG. **c** PRMT1 methylates cGAS in vitro. Purified GST-cGAS protein was incubated with PRMT1 protein and SAM for 1.5 hours, followed by immunoblot analysis with indicated antibodies. **d** PRMT1 methylates cGAS in an enzyme activity-dependent manner. Immunoblot analysis of the HA immunoprecipitant and WCL derived from HEK293T cells that ectopically express HA-cGAS and GFP-PRMT1 or indicated mutant constructs. **e** Inhibition of PRMT1 blocks cGAS methylation. Immunoblot analysis of the HA immunoprecipitant and WCL derived from HEK293T cells with the treatment of MS023 (6 µM) or GSK3368715 (6 µM) for 24 hours. **f** Stable overexpression of PRMT1 represses cGAS/STING DNA sensing signaling. HeLa cells stably expressing either GFP or HA-PRMT1 were stimulated with either HT-DNA (1×: 1 μg/mL; 2×: 2 μg/mL), or ISD (0.2 μg/mL) for 12 hours, followed by immunoblot analysis. **g** Immunoblot analysis of HeLa stable cell lines that expresses either GFP, HA-PRMT1-WT, or the catalytic-dead mutant HA-PRMT1-E162Q, with or without stimulation with HT-DNA (1 μg/mL) for 12 hours, followed by immunoblot analysis. **h** PRMT1 overexpression reduces DNA-stimulated cGAMP production. HeLa stable cells lines as in **f** were stimulated with 1 μg/mL of HT-DNA for 12 hours, followed by ELISA analysis to measure cGAMP levels. *n* = 3. **i** PRMT1 overexpression reduces DNA-stimulated expression of type I/II interferon response genes. HeLa stable cell lines as in **f** were stimulated with 1 μg/mL of HT-DNA for 12 hours, followed by qPCR analysis to measure the mRNA levels of CCL5 and CXCL10. *n* = 4. Data are presented as mean values ± SD for **h** and **i**. Two-tailed unpaired Student *t* test were used in **h** and **i**. Source data are provided as a Source Data file.

We speculate that PRMT1 might mediate cGAS arginine methylation to regulate innate immunity. To examine this hypothesis, we co-transfected HA-cGAS and GFP-PRMTs in HEK293T cells that lack endogenous cGAS and STING expression to avoid downstream inflammation signaling. Notably, we found that cGAS bound with only PRMT1 and PRMT2, but not other PRMTs we tested. Moreover, only PRMT1 triggered the asymmetric dimethylation on the arginine residues (ADMA) of both human and mouse cGAS (Fig. 1a, Supplementary Fig. 1a, b). In keeping with this finding, PRMT1 bound with endogenous cGAS (Fig. 1b) and methylated cGAS in vitro (Fig. 1c, Supplementary Fig. 1c). Moreover, the three catalytic-dead mutants of PRMT1, namely G98R, E162Q, and E171A^37,38^, were incapable of binding with or methylating cGAS (Fig. 1d, Supplementary Fig. 1d). Meanwhile, PRMT1-mediated arginine methylation of cGAS could be totally abolished by the PRMT1 inhibitors, MS023^39^ and GSK3368715^40^ (Fig. 1e), indicating that PRMT1 likely promotes the arginine methylation of cGAS in a catalysis-dependent manner.

To further explore whether and how PRMT1-mediated cGAS methylation affects cGAS-dependent DNA sensing signaling in cancer cells, we established a HeLa cell line that stably over-expressed PRMT1 (Supplementary Fig. 1e) and found that PRMT1 overexpression robustly repressed cellular DNA sensing, reflected by reduced phosphorylation of STING and IRF3 after stimulation with DNA, including HT-DNA and ISD45 (Fig. 1f, Supplementary Fig. 1f, g). In contrast, stable overexpression of the catalytic-dead PRMT1-E162Q mutant was incapable of inhibiting DNA sensing (Fig. 1g, Supplementary Fig. 1h-j), indicating that the observed suppressive effect of PRMT1 on cGAS/STING DNA sensing pathway is likely catalysis-dependent. In further support of the role of PRMT1 in directly suppressing cGAS enzymatic activity rather than indirectly affecting the activities of components of its downstream STING/TBK1/IRF3 signaling, we found that PRMT1 overexpression reduced cGAMP production after DNA stimulation (Fig. 1h). In line with this finding, PRMT1 overexpression subsequently repressed the phosphorylation of STING and IRF3, and reduced the transcription of type I interferon response genes, including CCL5 and CXCL10 (Fig. 1i, Supplementary Fig. 1k).

### Genetic ablation or pharmaceutic inhibition of PRMT1 leads to activation of cGAS/STING signaling

**Fig. 2:**
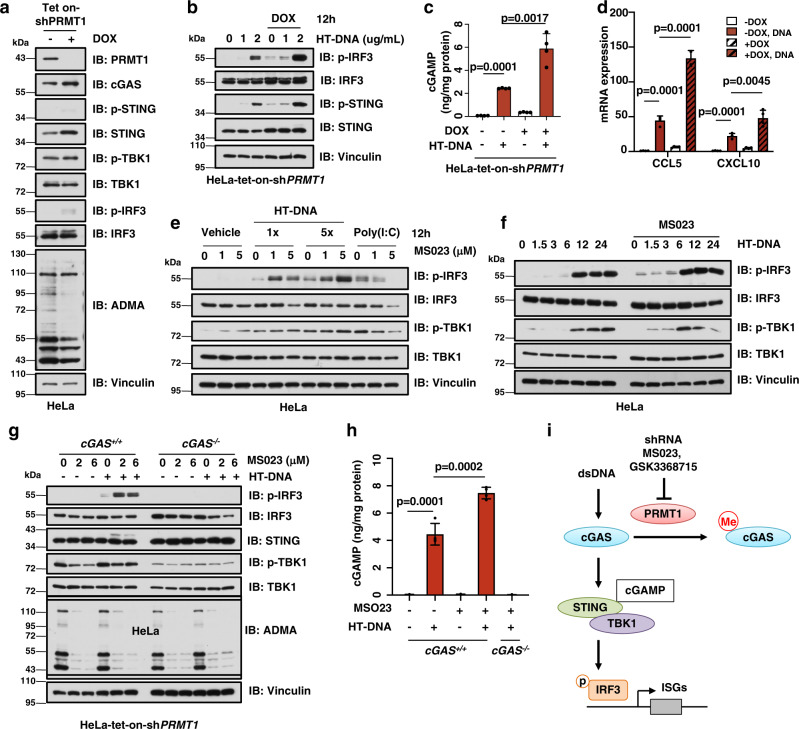
Genetic ablation or pharmaceutic inhibition of PRMT1 leads to activation of cGAS/STING signaling. **a** Immunoblot analysis of HeLa-tet-on-sh*PRMT1* stable cell lines. HeLa cells were infected with tet-inducible sh*PRMT1* and treated with doxycycline (DOX) for 3 days, followed by immunoblot analysis. **b** Genetic ablation of *PRMT1* activates cGAS/STING signaling. The stable HeLa cell as in **a** were stimulated with 1 μg/mL of HT-DNA for 12 hours, followed by immunoblot analysis. **c** Genetic ablation of *PRMT1* increases DNA-stimulated cGAMP production. The stable HeLa cells were treated as in **b**, followed by ELISA analysis to measure cGAMP levels. *n* = 4. **d** Genetic ablation of *PRMT1* increases DNA-stimulated expression of type I interferon response genes. The stable HeLa cells were treated as in **b**, followed by qPCR analysis to measure the mRNA levels of CCL5 and CXCL10. *n* = 4. **e** PRMT1 inhibition activates cGAS/STING signaling. HeLa cells were treated with 1 or 5 μM of MS023 for 48 hours, followed by stimulation with HT-DNA (1×: 1 μg/mL; 5×: 5 μg/mL) or Poly(I:C) for 12 hours, followed by immunoblot analysis. **f** PRMT1 inhibition activates cGAS/STING signaling in a time-dependent manner. HeLa cells were treated with 5 μM of MS023 for 48 hours, followed by stimulation with 1 μg/mL of HT-DNA for indicated hours, followed by immunoblot analysis. **g** PRMT1 inhibition activates cGAS/STING signaling in a cGAS-dependent manner. HeLa-*cGAS*-WT or *cGAS*-KO cells were treated with 2 or 6 μM of MS023 for 48 hours, then stimulated with 1 μg/mL of HT-DNA for 12 hours, followed by immunoblot analysis. **h** PRMT1 inhibition increases DNA-stimulated cGAMP production in a cGAS-dependent manner. HeLa-*cGAS*-WT or *cGAS*-KO cells were treated with 2 μM of MS023 for 48 hours, then stimulated with 1 μg/mL of HT-DNA for 12 hours, followed by ELISA analysis to measure cGAMP levels. *n* = 4. **i** A schematic diagram shows that PRMT1 methylates and suppresses cGAS function. Data are presented as mean values ± SD for **c**, **d**, **h**. Two tailed unpaired Student *t* test were used in for **c**, **d**, **h**. Source data are provided as a Source Data file.

To further determine the role of PRMT1 in controlling cGAS/STING signaling, we depleted the endogenous *PRMT1* using shRNA, and found that genetic deletion of PRMT1 activated the cGAS/STING/IRF3 signaling in HeLa cells in a time-dependent manner (Supplementary Fig. 2a–c). To exclude the potential effect of *PRMT1* ablation on cells proliferation rate, we further generated doxycycline (DOX)-inducible *PRMT1* knockdown cells and found DOX-induced transient *PRMT1* depletion mildly affected cell proliferation (HeLa-tet-on-sh*PRMT1*, Fig. 2a, Supplementary Fig. 2d). Similar like the constantly shRNA knockdown effect, DOX-induced *PRMT1* knockdown induced activation of cGAS-dependent DNA sensing signaling at basal and DNA-stimulated situations (Fig. 2b, Supplementary Fig. 2e, f). Moreover, deletion of *PRMT1* elevated cGAMP production (Fig. 2c) and transcription of type I interferon response genes (Fig. 2d). In echo with genetic *PRMT1* ablation, pharmaceutical inhibition of PRMT1 with the specific small molecule inhibitors, MS023^39^ and GSK3368715^40^, also led to a similar activation of DNA sensing signaling, but not RNA sensing signaling in dose- and time-dependent manners (Fig. 2e, f, Supplementary Fig. 2g–l). More importantly, PRMT1 inhibition-derived activation of DNA sensing signaling could be completely abolished by depleting endogenous *cGAS* (Fig. 2g, Supplementary Fig. 2m). Furthermore, PRMT1 inhibition also increased cGAMP production in *cGAS*^*+/+*^, but not *cGAS*^*−/−*^ cells (Fig. 2h, Supplementary Fig. 2n). These data together suggest that PRMT1 suppresses the cGAS/STING/IRF3 DNA sensing signaling in cancer cells via direct arginine methylation of cGAS (Fig. 2i).

### PRMT1 methylates cGAS at the conserved Arg133 residue on its N-terminus

**Fig. 3:**
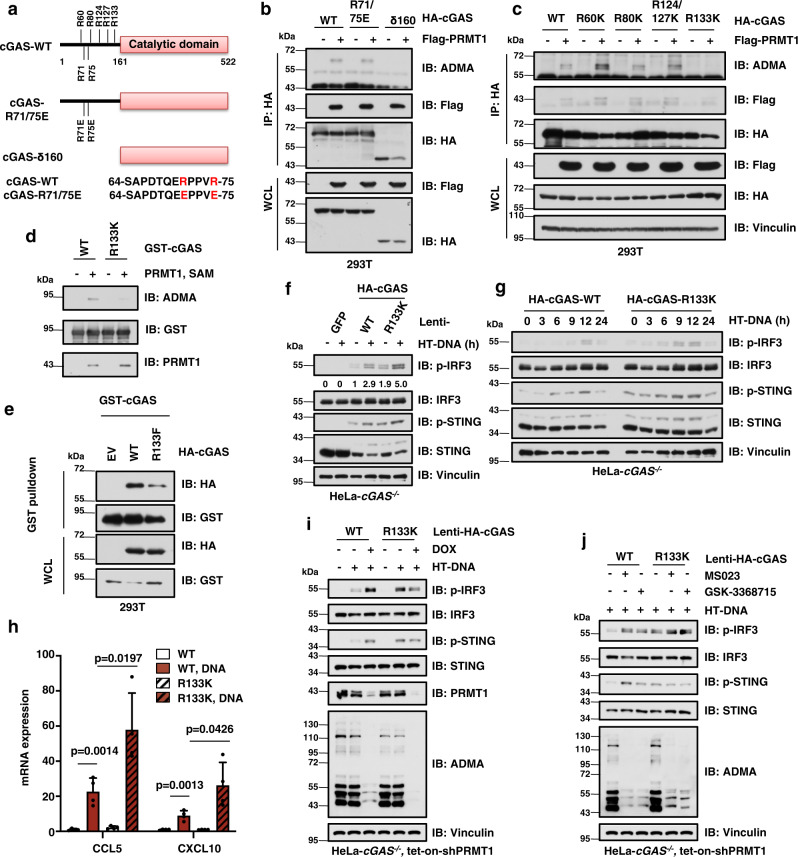
PRMT1 methylates cGAS at the conserved R133 residue to inhibit the cGAS/STING signaling. **a** Schematic diagram shows the conserved arginine residues in the N-terminus of cGAS. cGAS-δ160: cGAS that lacks the N-terminal amino acids 1–160. R71/75E: arginine 71/51 to glutamic acid mutant. **b** PRMT1 methylates cGAS at its N-terminus. Immunoblot analysis of the HA immunoprecipitant and WCL derived from HEK293T cells that ectopically express Flag-PRMT1 and HA-cGAS. **c** The conserved R133 in cGAS is a major site for PRMT1-mediated asymmetric dimethylation. **d** Mutation of the R133 residue largely reduces PRMT1-mediated methylation on cGAS in the in vitro methylation assay. **e** Mutation of the R133 residue represses cGAS dimerization. Immunoblot analysis of the GST pulldown products and WCL derived from HEK293T cells that ectopically express GST-cGAS together with HA-cGAS-WT or the R133F mutant constructs. R133F: arginine 133 to phenylalanine mutant. **f** Disruption of cGAS-R133 methylation activates cGAS/STING signaling. HeLa-*cGAS*^−/−^ cells expressing either HA-cGAS-WT or R133K were stimulated HT-DNA and followed by immunoblot analysis. **g** Disruption of cGAS arginine methylation activates cGAS/STING signaling in a time-dependent manner. The stable HeLa cell lines as in **f** were stimulated with HT-DNA for indicated times, followed by immunoblot analysis. **h** Disruption of cGAS arginine methylation increases DNA-stimulated expression of type I interferon response genes. The stable HeLa cell lines as in **f** were stimulated with HT-DNA, followed by qPCR analysis. Two-tailed unpaired Student *t* test, *n* = 4. Data are presented as mean values ± SD. **i** Mutation of cGAS-R133 abolished PRMT1-mediated regulation of the cGAS/STING signaling. The stable HeLa cell lines as in **f** were infected with tet-inducible sh*PRMT1* lentivirus, treated with DOX, and stimulated with HT-DNA, followed by immunoblot analysis. **j** PRMT1 inhibition activates the cGAS/STING signaling in cGAS-WT expressing cells, but not in the cGAS-R133K mutant expressing cells. The stable HeLa cell lines as in **f** were treated with MS023 (6 µM) or GSK3368715 (6 µM) for 48 hours and stimulated with HT-DNA, followed by immunoblot analysis. Source data are provided as a Source Data file.

Two conserved arginine residues in the N-terminus of cGAS, namely Arg-71 (R71) and Arg-75 (R75), are critical for cGAS anchoring on the plasma membrane^25^. Thus, we tested the potential role of R71/75 in PRMT1-mediated methylation by in vivo methylation assay (Fig. 3a, b). Notably, the methylation was unaffected by mutation of R71/75 to glutamic acid (E) but eliminated by truncation of the N-terminal 160 amino acids of cGAS (namely cGAS-δ160, Fig. 3a, b), suggesting that PRMT1 methylates cGAS on its N-terminus. The N-terminus of cGAS is essential for its function and regulation^20,24^, and contains multiple conserved arginine residues, including R60, R80, R124, R127, and R133 (Fig. 3a, Supplementary Fig. 3a, b). By mutating these arginine residues to lysine (K), we identified the R133 residue (R139 in mouse) as a major site for PRMT1-mediated methylation on cGAS (Fig. 3c, Supplementary Fig. 3c), which was further validated by the in vitro methylation assay and mass spectrometry analysis (Fig. 3d, Supplementary Fig. 3d–f).

Mechanistically, the methylation-mimic mutant cGAS-R133F repressed the dimerization of cGAS (Fig. 3e), but had relatively little effect on its DNA binding capability or subcellular localization (Supplementary Fig. 3g–i), suggesting that the methylation on the R133 might perturb cGAS homodimerization. Functionally, a stable HeLa cell line expressing the methylation deficient mutant cGAS-R133K had a relatively higher basal level and DNA-stimulated levels of p-STING and p-IRF3 in dose- and time-dependent manners (Fig. 3f, g, Supplementary Fig. 3j-m), thus elevating the expression of type I interferon response genes, CCL5 and CXCL10 (Fig. 3h). In contrast, the stable cell lines expressing methylation-mimic cGAS-R133F mutant had a relatively lower level of p-IRF3 than in WT cells (Supplementary Fig. 3n, o). Meanwhile, further depletion of *PRMT1* only activates cGAS/STING DNA sensing signaling in HeLa cells expressing WT-cGAS, but not in HeLa cells expressing the cGAS-R133K mutant (Fig. 3i). Consistently, pharmaceutical inhibition of PRMT1 by MS023 and GSK3368715 elevated the phosphorylation of IRF3 only in HeLa cells expressing wild-type (WT)-cGAS, but not in HeLa cells expressing the cGAS-R133K mutant (Fig. 3j). Taken together, these data indicate that PRMT1-mediated methylation on the conserved R133 residue of cGAS suppresses the cGAS/STING DNA sensing signaling.

### PRMT1 suppresses tumor immunity in a cGAS-dependent manner

**Fig. 4:**
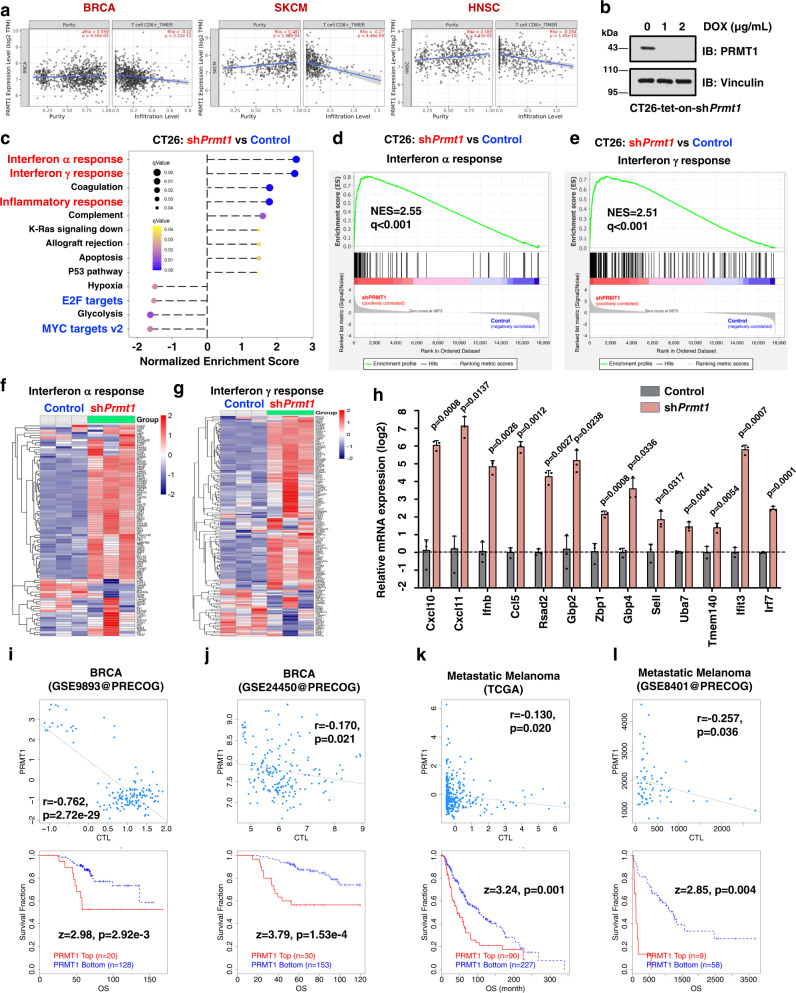
PRMT1 represses tumor immune cells infiltration in human tumor samples and in mouse tumor cells. **a** PRMT1 expression is inversely correlated with CD8+ T cells infiltration in multiple cancer types in TCGA by TIMER2. SKCM: skin cutaneous melanoma, HNSC: head and neck squamous cell carcinoma, BRCA: breast invasive carcinoma. Spearman correlation rho and *p* value are presented. Error band represents the 95% confidence interval. **b** Immunoblot analysis of CT26-tet-on-sh*Prmt*1 cells after treatment with doxycycline for 48 hours. CT26 mouse tumor cells were infected with tet-inducible-sh*Prmt1* lentivirus and selected with 5 μg/mL puromycin for 7 days. The stable CT26-tet-on-sh*Prmt1* cell lines were treated with indicated doses of DOX for 3 days and harvested for immunoblot analysis. **c** Genetic ablation of *Prmt1* activates the expression of type I and II interferon genes. The stable CT26-tet-on-sh*Prmt1* cell lines were treated with DOX for 3 days to induce deletion of endogenous *Prmt1*, then subjected to RNA-sequencing and Gene set enrichment analysis (GSEA). **d** Gene set enrichment plots of the IFN-α response hallmarks after genetic ablation of *Prmt1* in CT26 cells as in **b**. NES = 2.55, *q* < 0.001. **e** Gene set enrichment plots of the IFN-γ response hallmarks after genetic ablation of *Prmt1* in CT26 cells as in **b**. NES = 2.51, *q* < 0.001. **f** Heatmap of IFN-α response genes after genetic ablation of *Prmt1* in CT26 cells as in **d**. **g** Heatmap of IFN-γ response genes after genetic ablation of *Prmt1* in CT26 cells as in **e**. **h** Depletion of *Prmt1* in CT26 mouse tumor cells induces the transcription of Interferon α and IFN-γ response genes. Two-tailed unpaired Student *t* test, *n* = 4. Data are presented as mean values ± SD. **i**, **j** Correlation analysis between PRMT1 expression with cytotoxic T lymphocytes (CTL) infiltration and survival in BRCA, derived from GSE9893 (**i**) and GSE24450 (**j**) at PRECOG by TIDE. **k**, **l** Correlation analysis between PRMT1 expression with CTL infiltration and survival in metastatic melanoma, derived from TCGA (**k**) and GSE8401 (**l**) by TIDE. **i**–**l** Spearman correlation rho and *p* value are presented. Source data are provided as a Source Data file.

Because the cGAS/STING pathway is essential for anti-tumor immunity^11,12^, we further determined whether PRMT1 is a rational target for immunotherapy. Through analyzing the role of PRMT1 in tumorigenesis and immune cell infiltration for patients in TCGA (see methods for details), we found PRMT1 was highly expressed in most cancer types, including breast cancer (BRCA, Supplementary Fig. 4a). More importantly, PRMT1 expression was negatively correlated with the infiltration of CD8^+^ T cells and macrophages in BRCA, skin cutaneous melanoma (SKCM), and head and neck squamous cell carcinoma (Fig. 4a, Supplementary Fig. 4b, c). Moreover, PRMT1 expression was reversely correlated with the effector T cell signature^41^ in BRCA and lung adenocarcinoma (Supplementary Fig. 4d).

We next determined the causal effect of PRMT1 in tumor immune surveillance in vitro and in vivo. To this end, we generated a mouse cancer cell line CT26 that stably expressed a DOX-inducible *shPrmt1* construct (hereafter CT26-tet-on-sh*Prmt1*), which proceeded to RNA-sequencing to analyze PRMT1-responsive genes (Fig. 4b). Notably, *Prmt1* depletion elevated the expression of type I and II interferons response genes (Fig. 4c–g), and other inflammation-related genes signatures, including inflammatory response and IL6-JAK-STAT3 signaling (Supplementary Fig. 4e–g). The elevated expression of type I and II interferon response genes was further validated by RT-qPCR after genetic deletion of *PRMT1* (Fig. 4h). In line with the critical role of PRMT1 in suppressing tumor immune surveillance, relatively higher PRMT1 expression level predicted lower cytotoxic T lymphocytes (CTL) infiltration and a worse prognosis for patients with BRCA (Fig. 4i, j), metastatic melanoma (Fig. 4k, l), and several other cancer types, including multiple myeloma (MM, Supplementary Fig. 4h) and neuroblastoma (NB, Supplementary Fig. 4i). Furthermore, the reverse correlation between PRMT1 expression and CTL infiltration also occurred in other cancer types, including acute myeloid leukemia (AML), ovarian cancer (OVCA), lung cancer (LUCA), bladder cancer (BLCA), liver cancer, and diffuse large B cell lymphoma (DLBC, Supplementary Fig. 4h–o), supporting the notion that PRMT1 acts as a suppressor of tumor immune surveillance.

### Pharmaceutic inhibition of PRMT1 triggers tumor immunity in a cGAS-dependent manner in vitro and in vivo

**Fig. 5:**
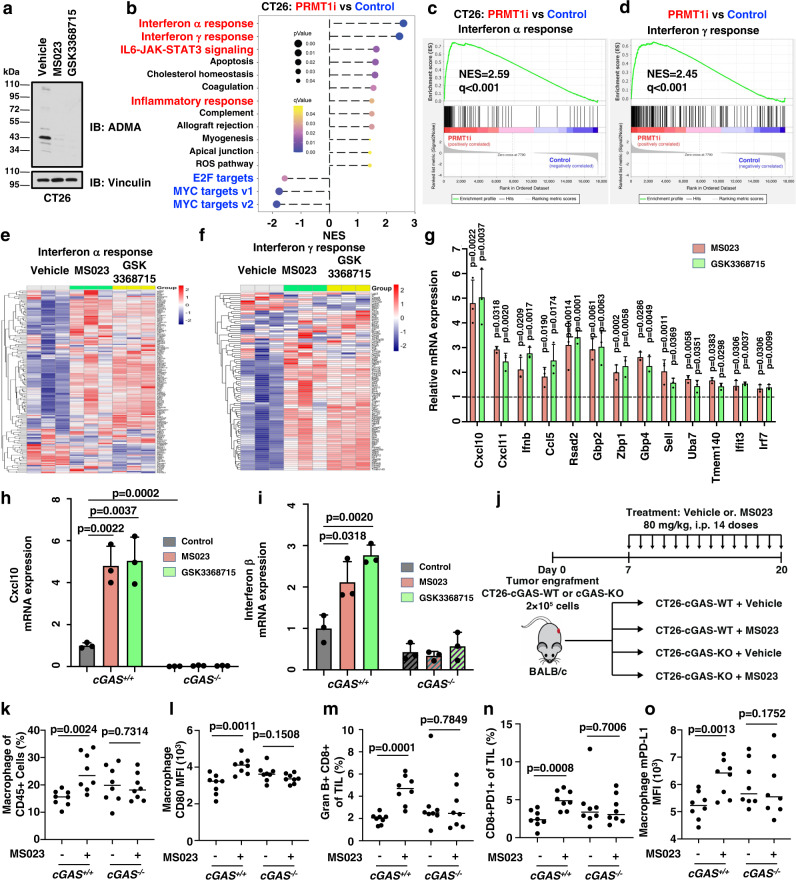
PRMT1 inhibition activates type I and II interferon response genes expression and promotes immune cell infiltration in a cGAS-dependent manner. **a** Immunoblot analysis of CT26 mouse tumor cells after treatment with 6 µM of MS023 or GSK3368715 for 48 hours. **b** PRMT1 inhibition activates the expression of type I and II interferon genes. CT26 cells were treated with MS023 (6 µM) or GSK3368715 (6 µM) for 48 hours, then subjected to RNA-sequencing and GSEA analysis. PRMT1i represents a total of 6 samples with either MS023 or GSK3368715 treatment. *n* = 3. **c**, **d** Gene set enrichment plots of the IFN-α (**c**) and IFN-γ response (**d**) hallmarks after treatment with PRMT1 inhibitors in CT26 cells. **e**, **f** Heatmap of IFN-α (**e**) and IFN-γ response (**f**) genes for CT26 cells after treatment with MS023 or GSK3368715 in CT26 cells as in **c** and **d**, respectively. **g** Inhibition of PRMT1 in CT26 mouse tumor cells induces the transcription of IFN-α and IFN-γ response genes. **h**, **i** PRMT1 inhibition increases Cxcl10 (**h**) and Interferon β (**i**) expression in a cGAS-dependent manner in CT26 cells. **j** A schematic diagram illustrates the animal experimental design for the tumor-infiltrating immune cells analysis. CT26-cGAS-WT and cGAS-KO cells were injected into BALB/c mice and tumors were harvested at 14 days after tumor engraftment, followed by isolation and staining of tumor-infiltrating immune cells. **k**, **l** PRMT1 inhibition induces macrophage infiltration (**k**) and activation (**l**) in CT26 cells-derived syngeneic tumor in a cGAS-dependent manner. **m**, **n** PRMT1 inhibition induces cytotoxic Gran B^+^CD8^+^ T cells (**m**) and CD8^+^PD-1^+^ T cells (**n**) in CT26 cells-derived syngeneic tumor in a cGAS-dependent manner. **o** PRMT1 inhibition increases mPD-L1 expression in tumor-infiltrated macrophages. Data are presented as mean values ± SD for **g**–**i**, *n* = 3. Data are presented as mean values with scatter dots for **k**–**o**, *n* = 8. Two-tailed unpaired Student *t* test were used in **g**–**o**. The animal experiments in **k**–**o** were replicated twice, and the representative data were presented. Source data are provided as a Source Data file.

To further explore the potential of PRMT1 as an immunotherapeutic target, we further treated CT26 cells with PRMT1 inhibitors, MS023 and GSK3368715, and performed RNA-sequencing for these samples (Fig. 5a). In line with the genetic ablation data, pharmaceutical inhibition of PRMT1 displayed the same phenotype, including elevated expression of type I and II interferons response genes and other inflammation-related gene signatures (Fig. 5b–f, Supplementary Fig. 5a–k). Interestingly, the expression levels of MYC-target genes were significantly reduced after genetic and pharmaceutical ablation of *PRMT1* (Fig. 5b, Supplementary Fig. 5c, d, h), which was consistent with previous reports depicting MYC as a suppressor of anti-tumor immunity^42,43^. Similarly, CDK4 and E2F1 also have been reported as downstream substrates of PRMT1^44,45^, thus the reduced gene signature of E2F targets might be due to the inactivation of CDK4/E2F signaling (Fig. 5b, Supplementary Fig. 5d). The elevated expression of type I and II interferon response genes was further validated by RT-qPCR after pharmaceutical inhibition of *PRMT1* (Fig. 5g). More importantly, PRMT1 inhibition-induced elevation in type I and II interferon response genes, including *Cxcl10*, *Ifnb,* and others, was completely abolished by depletion of endogenous *cGAS* (Fig. 5h, I, Supplementary Fig. 5l–u). These data suggest that genetic or pharmaceutical ablation of *PRMT1* increases the expression of type I and II interferon response genes in a cGAS-dependent manner, which might predict a better response to cancer immunotherapy.

To demonstrate the inhibitory effect of PRMT1 in cancer immune surveillance through methylation of cGAS in vivo, we measured immune cell infiltration in a syngeneic mouse model of engraftment with either CT26-*cGAS*-WT or CT26-*cGAS*-KO cells after treatment with PRMT1 inhibitor MS023 (Fig. 5j, Supplementary Fig. 5v). We found that PRMT1 inhibitor treatment stimulated macrophages infiltration in only CT26-*cGAS*-WT cell-engrafted tumor, but not the CT26-*cGAS*-KO cell-engrafted tumor (Fig. 5k). Moreover, PRMT1 inhibition increased the CD80 MFI in macrophages, indicating a relatively higher level of CD80 activation and cytotoxic T cells (GranB^+^CD8^+^) in CT26-*cGAS*-WT, but not the CT26-*cGAS*-KO cell-engrafted tumor (Fig. 5l, m). Notably, PRMT1 inhibition also led to an increased population of the CD8+ PD-1+ T cells in CT26-*cGAS*-WT, but not the CT26-*cGAS*-KO cell-engrafted tumor (Fig. 5n). This observation is consistent with the reverse correlation between PRMT1 level and macrophages or effector T cells infiltration in cancer patients (Fig. 4a, Supplementary Fig. 4b–d). Notably, we also observed an elevation of PD-L1 abundance in infiltrating macrophages after PRMT1 inhibition in CT26-engrafted tumors (Fig. 5o), which prompted us to further determine whether and how PRMT1 regulates PD-L1 expression.

### *PRMT1* ablation increases PD-L1 expression in a cGAS-dependent manner

**Fig. 6:**
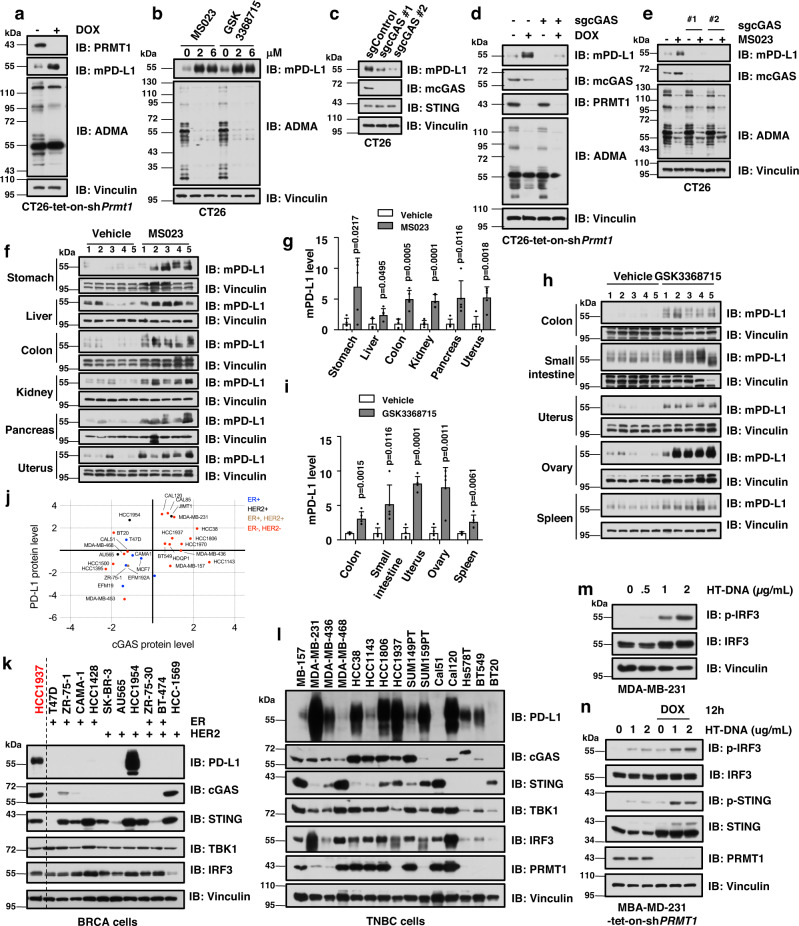
PRMT1 inhibition increase PD-L1 in a cGAS-dependent manner. **a** Genetic ablation of *Prmt1* elevates mPD-L1 expression. The stable CT26-tet-on-sh*Prmt1* cells were treated with DOX for 3 days to induce deletion of endogenous *Prmt1*, followed by immunoblot analysis. **b** PRMT1 inhibition elevates mPD-L1 expression. CT26 cells were treated with the indicated doses of MS023 or GSK3368715 for 48 hours, followed by immunoblot analysis. **c** Depletion of endogenous *cGAS* reduces mPD-L1 expression. **d** Genetic ablation of *Prmt1* elevates mPD-L1 expression in a cGAS-dependent manner. CT26-tet-on-sh*Prmt1* cells as in **a** were infected with sg*cGAS* lentivirus and treated with DOX, followed by immunoblot analysis. **e** PRMT1 inhibition elevates mPD-L1 expression in a cGAS-dependent manner. The stable cell lines as in **c** were treated with MS023, followed by immunoblot analysis. **f**, **g** Immunoblot analysis of mPD-L1 (**f**) and quantification (**g**) in different organs/tissues of mice after i.p. administration of 50 mg/kg MS023 or vehicle for 14 days. **h**, **i** Immunoblot analysis of mPD-L1 (**h**) and quantification (**i**) in different organs/tissues of mice after gavage of 80 mg/kg GSK3368715 or vehicle for 14 days. **j** Correlation analysis of cGAS and PD-L1 protein expression in human BRCA cell lines. The protein levels were retrieved from DepMap. ER+: estrogen receptor positive; HER2+: human epidermal growth factor receptor 2 positive; ER+, HER2+: ER and HER2 double positive; ER−, HER2−: ER and HER2 negative. **k**, **l** Immunoblot analysis of PD-L1 and cGAS in a panel of BRCA (**k**) and TNBC cell lines (**l**). **m** Immunoblot analysis of MDA-MB-231 cells after stimulation with indicated doses of HT-DNA for 12 hours. **n** Genetic ablation of *PRMT1* increases cGAS/STING DNA sensing signaling in MBA-MD-231 cells. MDA-MB-231 cells were infected with tet-on-sh*PRMT1* lentivirus and selected with 1 μg/mL of puromycin for 7 days. The stable cell lines were treated with DOX for 3 days, and stimulated with HT-DNA for 12 hours, followed by harvesting for immunoblot analysis. Data are presented as mean values ± SD, and two-tailed unpaired Student *t* test were used in for 6 **g** and **i**, *n* = 5.

To investigate how PRMT1 regulates PD-L1 expression, we next generated multiple mouse tumors cells, including CT26, MC38, 4T1, and B16, that stably expressed tet-inducible sh*Prmt1* (Supplementary Fig. 6a–d), and found that mPD-L1 expression was robustly elevated after doxycycline-induced knockdown of *Prmt1* in these mouse tumor cells (Fig. 6a, Supplementary Fig. 6b–d). Similarly, pharmaceutical inhibition of PRMT1 also increased mPD-L1 expression in these tumor cells (Fig. 6b, Supplementary Fig. 6e–g). To further determine whether PRMT1-mediated change in PD-L1 expression is cGAS-dependent, we depleted endogenous *cGAS* in CT26, MC38, and 4T1 mouse tumor cells, and found a significant reduction in mPD-L1 expression in these cells (Fig. 6c, Supplementary Fig. 6h–j). More importantly, genetic ablation or pharmaceutical inhibition of *PRMT1* increased mPD-L1 expression only in *cGAS-WT* cells, but not in *cGAS-KO* cells (Fig. 6d, e, Supplementary Fig. 6k–m). Moreover, mPD-L1 expression was also elevated in multiple mouse organs/tissues in mice treated with PRMT1-specific inhibitors, MS023 and GSK3368715 (Fig. 6f–i). In line with this finding, PD-L1 expression was positively correlated with cGAS level in human BRCA samples and BRCA cell lines (Fig. 6j, Supplementary Fig. 6n, o). Interestingly, the TNBC cells were largely cGAS-positive and PD-L1-positive, while most HER2-positive or ER2-positive BRCA cells had neither cGAS nor PD-L1 expression (Fig. 6j–l, Supplementary Fig. 6o). Moreover, these cGAS-positive TNBC cells, including MDA-MB-231, have intact cGAS/STING DNA sensing signaling (Fig. 6m, Supplementary Fig. 6p–r). Similar to the results in HeLa cells, DOX-induced genetic ablation of *PRMT1* in MDA-MB-231 cells also activated DNA sensing signaling, but not RNA sensing signaling (Fig. 6n, Supplementary Fig. 6s). These results imply that PRMT1 regulates PD-L1 expression in a cGAS-dependent manner, which might account for the repressive role of PRMT1 in cancer immune surveillance.

### PRMT1 inhibitor synergizes with anti-PD-1 antibody to boost anti-tumor immunity

**Fig. 7:**
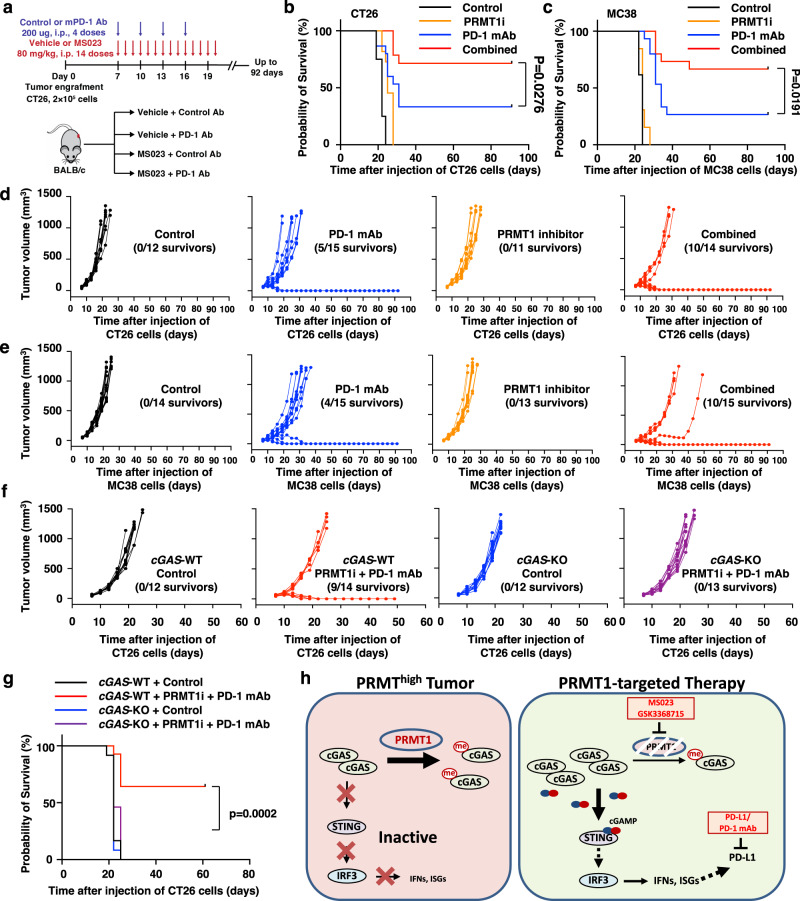
PRMT1 inhibition boosts the efficacy of anti-PD-1 immunotherapy in vivo in a cGAS-dependent manner. **a** A schematic diagram illustrates the animal experiment design for the CT26 syngeneic tumor model with PRMT1 inhibitor and anti-PD-1 antibody treatment. **b** Kaplan–Meier survival curves for each treatment group in CT26 syngeneic tumor model (Control, *n* = 12; PD-1 monoclonal antibodies (mAb), *n* = 15; MS023, *n* = 11; Combined, *n* = 14). Two-sided Gehan-Breslow-Wilcoxon test. **c** Kaplan–Meier survival curves for each treatment group in MC38 syngeneic tumor model (Control, *n* = 14; PD-1 mAb, *n* = 15; MS023, *n* = 13; Combined, *n* = 15). Two-sided Gehan-Breslow-Wilcoxon test. **d** Volumes of CT26 syngeneic tumors treated with vehicle + control antibodies (black lines, *n* = 12), or vehicle + anti-PD-1 mAb (blue lines, *n* = 15), or PRMT1 inhibitor MS023+ control antibodies (orange lines, *n* = 11), or combined therapy (red lines, *n* = 14) were plotted individually. **e** Volumes of the MC38 syngeneic tumors treated with vehicle + control antibodies (black lines, *n* = 14), vehicle + anti-PD-1 mAb (blue lines, *n* = 15), PRMT1 inhibitor MS023 + control antibodies (orange lines, *n* = 13) or combined therapy (red lines, *n* = 15) were plotted individually. **f** Volumes of CT26-*cGAS*-WT and *cGAS*-KO syngeneic tumors treated with vehicle + control antibody or MS023 + anti-PD-1 mAb were plotted individually. *cGAS*-WT + vehicle and control antibody, *n* = 12, black lines; *cGAS*-WT + MS023 and anti-PD-1 mAb, *n* = 14, red lines; *cGAS*-KO + vehicle and control antibody, *n* = 12, blue lines; *cGAS*-KO + MS023 and anti-PD-1 mAb, *n* = 13, purple lines. **g** Kaplan–Meier survival curves for each treatment group in CT26-*cGAS*-WT and *cGAS*-KO syngeneic tumor model (*cGAS*-WT + vehicle and control antibody, *n* = 12; *cGAS*-WT + MS023 and anti-PD-1 mAb, *n* = 14; *cGAS*-KO + vehicle and control antibody, *n* = 12; *cGAS*-KO + MS023 and anti-PD-1 mAb, *n* = 13). Two-sided Gehan-Breslow-Wilcoxon test. **h** A schematic diagram shows that PRMT1 represses cGAS/STING signaling to promote tumor immune evading, while the combination therapy of PRMT1i and ICB boosts the immune response to promote tumor regression. The animal experiments in **a**–**g** were replicated twice, and the representative data were presented. Source data are provided as a Source Data file.

Given the critical role of PRMT1 in cGAS-dependent DNA sensing signaling and immune suppression in human and mouse tumor cells (Figs. 4 and 5), we next examined the potential of PRMT1 as a target of cancer immunotherapy. We noticed that PRMT1 inhibition strongly stimulated macrophage infiltration (Fig. 5k, l) and elevated PD-L1 expression in vitro and in vivo (Fig. 6, Supplementary Fig. 6), which provided a rationale for combination therapy using PRMT1 inhibitor and ICBs, such as anti-PD-1 antibody. Thus, we treated CT26- and MC38-derived syngeneic tumor models with either PRMT1 inhibitor MS023, or anti-PD-1 antibody, or their combination and monitored tumor growth and mouse survival for up to 90 days (Fig. 7a, Supplementary Fig. 7a). Notably, PRMT1 inhibition alone restricted neither tumor growth nor overall survival of CT26 or MC38, while the combination of PRMT1 inhibition and anti-PD-1 antibody substantially slowed tumor growth and increased the survival rate in both CT26 and MC38 tumor-engrafted mouse models (Fig. 6n–e, Supplementary Fig. 6b–e).

In line with the results of syngeneic tumor models, higher cytotoxic T lymphocytes (CTLs) infiltration predicted better prognosis only when PRMT1 expression level was low in human cancer patients, including COAD, AML, lymphoma, and glioma (Supplementary Fig. 6f–j). Notably, high PRMT1 expression also compromised the response efficacy of anti-PD-L1 antibody treatment in human bladder cancer^46^ (Supplementary Fig. 6k, l), possibly due to an inverse relation between PRMT1 expression and CTLs infiltration level in tumors (Fig. 4, Supplementary Fig. 4). Finally, we compared the responsiveness for the combination treatment of PRMT1 inhibitor and anti-PD-1 antibody between CT26-*cGAS*-WT and CT26-*cGAS*-KO syngeneic tumor models (Fig. 7m). We found that the combination therapy robustly slowed the tumor growth and increased the survival rate only in CT26-*cGAS*-WT tumor model, while *cGAS*-KO tumor-engrafted mice did not respond to the combination therapy (Fig. 7f, g, Supplementary Fig. 7n, o), supporting the notion that the therapeutic benefit of PRMT1 inhibition in the syngeneic tumor model was largely cGAS-dependent (Fig. 7h, Supplementary Fig. 7p).

## Discussion

PRMT1 is known as a regulator of immune function by directly interacting with interferon receptors, methylating STAT1, and promoting B cell and macrophage differentiation by methylating CDK4 or B cell antigen receptor^44,47–50^. However, the function of PRMT1 as a therapeutic target of cancer remains largely elusive, particularly for its role in cancer immunosurveillance. Here we revealed a critical role of PRMT1 in directly methylating cGAS and blocking cGAS/STING DNA sensing signaling, thus promoting tumor immune evasion, which provides a rationale for PRMT1 as a promising immunotherapeutic target. Several chemotherapy and targeted therapy drugs have been reported to activate STING signaling to boost tumor immunity, including etoposide and PARP inhibitor in LUCA and BRCA^15,16^. Given that the basal level of cGAS/STING signaling in tumors is suppressed, at least partially, by PRMT1-mediated cGAS methylation, PRMT1 inhibition is an attractive therapeutic choice to potentially synergize with these cytosolic DNA-elevating therapies, which might produce better immunogenicity in the tumor microenvironment for cancer patients. Indeed, PRMT1 inhibitor displays the most potent synergistic effect with PARP inhibitor for BRCA and LUCA cells in vitro^51,52^ and PRMT1 overexpression confers the chemoresistance to cisplatin^53^. Moreover, PRMT1 inhibition induces a viral mimicry response in human TNBC cells^36^. Thus it is worth further in-depth investigation of PRMT1-focused combination therapy in boosting anti-tumor immunity, especially in an immune-competent condition in vivo. In addition, PRMT1 governs spliceosome function, and PRMT1 inhibition boosts anti-tumor immunity through MHC-I-mediated neo-antigen presentation^54^. Recently, PRMT5, the major type II protein arginine methyltransferase, has also been reported as a suppressor of anti-tumor immunity in melanoma in a cGAS-independent manner through methylating IFI16 (a parallel signaling of cGAS/STING) and NLRC5 to block the transcription of type I interferons and major histocompatibility complex class I (MHC-I)^30–32^. Our defined PRMT1-cGAS signaling axis might partially explain the synergistic effect of inhibition of PRMT1 and PRMT5^40^, in which both the parallel cGAS and IFI16 signaling might be activated to maximal downstream cascade to trigger anti-tumor immunity. Given that intratumor PD-L1 expression level dictates the sensitivity to immune checkpoint blockade therapies, such as anti-PD-1 and anti-PD-L1 antibodies^55–57^, other therapies that increase PD-L1 expression might be a rational choice to be used together with immunotherapy. For example, CDK4/6 inhibition elevates PD-L1 expression and enhances the therapeutic outcomes of anti-PD-1 antibody^58,59^. Apart from boosting antigen presentation through type I/II interferon pathway, depletion or inhibition of PRMT1 also led to elevation in PD-L1 expression in vitro and in vivo, thus synergizing with the anti-PD-1 antibody in various syngeneic mouse models (Figs. 6 and 7). In our study, PRMT1 inhibitor plus the PD-1 antibody combined immunotherapy has been evaluated in two syngeneic mouse models, CT26 and MC38, which have relatively high immunogenicity and well response rate to the PD-1 antibody. In a recent study, PRMT1 inhibitor and the PD-1 antibody has been reported to be effective in suppressing B16-derived xenograft syngeneic mouse model^54^, indicating that this combined immunotherapy might be a valid option for tumor with either higher or low immunogenicity. Now, PRMT1 inhibitor is in phase I clinic trial^40^, and its effect as a combination immunotherapy awaits further in-depth investigation.

## Methods

### Cell lines

HEK293T, HeLa cells, mouse embryonic fibroblasts (MEFs), MB157, MDA-MB-231, MDA-MB-436, MDA-MB-436, Cal51, Cal120, Hs587T, BT-549, BT-20, T47D, SK-BR-3, iBMDM, CT26, MC38, 4T1, and B16F10 cell lines were cultured in Dulbecco’s Modified Eagle’s Medium (DMEM) containing 10% fetal bovine serum (FBS), 100 Units of penicillin and 100 µg/ml streptomycin. HCC1954, ZR-75-1, MDA-MB-468, HCC38, HCC1143, HCC1806, and HCC1937 cells were cultured in RPMI1640 containing 10% FBS, 100 Units of penicillin, and 100 µg/ml streptomycin. SUM149 cells were cultured in DMEM/F12 media containing 5% FBS, 5 µg/ml insulin, 1 µg/ml hydroxylcortisol, 100 Units of penicillin, and 100 µg/ml streptomycin. All the cell lines were authenticated and validated for mycoplasma negative.

### General cloning

Expression plasmids for GFP-PRMT1, 2, 3, 4, and 5 were kindly gift from Dr. Yanzhong Yang (City of Hope Cancer Center). Expression plasmids for C-terminal tagged HA-cGAS, HA-cGAS-R71/75E and cGAS-δ160 were kindly gift from Dr. Jonathan C. Kagan (Harvard Medical School). GFP-PRMT1-G98R, E162Q, E171A, HA-cGAS-R60K, R80K, R124/127 K, R133K, and R133F were constructed using the Site-Directed Mutagenesis Kit (Agilent) according to the manufacturer’s manual. Expression vectors for N-terminal tagged HA-cGAS was constructed by cloning the corresponding cDNAs into pcDNA3-HA vector. Expression plasmids for mouse cGAS were constructed by cloning the corresponding cDNAs from pMSCV-eGFP-mcGAS (Addgene, #108675) into pcDNA3-HA vector. Virus packaging vectors for HA-PRMT1, HA-PRMT1-E162Q, HA-cGAS, HA-cGAS-R133K, and HA-cGAS-R133F were constructed by cloning the corresponding pLenti-GFP vector. Mammalian expression vector for GST-cGAS were constructed by cloning the corresponding cDNA into pCMV-GST vector. Bacterial expression vectors for GST-cGAS and GST-cGAS-R133K were constructed by cloning the corresponding cDNA into pGEX-GST-4T1 vector. The shPRMT1 constructs were purchased from Sigma. The doxycycline-inducible knockdown plasmids for PRMT1 were constructed by subclone respective sequences into pLKO-tet-on-shRNA vector^60^. The sgRNA constructs for human and mouse cGAS were generated by inserting respective sgRNA into pLenti-CRISPR-v2 vector^61^.

### Antibodies

The anti-ADMA (13522), anti-human cGAS (15102), anti-mouse cGAS (31659), anti-STING (13647), anti-p-STING (Ser366, 50907), anti-IRF3 (11904), anti-mouse p-IRF3 (Ser 396, 29047), anti-TBK1 (3504), anti-p-TBK1 (Ser172, 5483), anti-PRMT1 (2449), anti-human PD-L1 (13684), anti-GST (2625) antibodies were obtained from Cell Signaling Technology. Anti-human p-IRF3 (Ser 396, ab76493) and anti-mouse PD-L1 (EPR20529, ab213480) antibodies were obtained from Abcam. Anti-Tubulin (sc-8035), anti-GFP (B-2, sc-9966), and anti-PRMT1 (B-2, sc-166963) antibodies were obtained from Santa Cruz Biotechnology. Mouse monoclonal anti-HA.11 epitope tag (16B12, 901513) was obtained from BioLegend. Anti-Vinculin (V9131), rabbit polyclonal anti-HA (H6908), Mouse monoclonal anti-Flag (F3165), Rabbit polyclonal anti-Flag (F7425), anti-mouse IgG (whole molecule)-peroxidase (A4416) and anti-rabbit IgG (whole molecule)-peroxidase (A4914) were obtained from Sigma-Aldrich. Mouse monoclonal ANTI-FLAG M2 affinity agarose gel (A2220) and Mouse monoclonal anti-HA-agarose (A2095) were obtained from Sigma-Aldrich. The antibodies used for FACS were as follow: anti-CD45 (30-F11, #103140, BioLegend); anti-CD3 BV785 (17A2, #100232, BioLegend); anti-CD3ε BV785 (145-2C11, #100355, BioLegend); anti-CD4 BV650 (GK1.5, #100469; RM4-5, #100555, BioLegend); anti-CD8a BV711 (53-6.7 #100748, BioLegend); anti-PD-L1 (10 F.9G2, # 124308, BioLegend); anti-Granzyme B Pacific Blue (GB11, #515408, BioLegend); anti-CD11b BV650 (M1/70, # 101259, BioLegend); anti-CD11c BV510 (N418, # 117353, BioLegend); anti-CD80 Pacific Blue (16-10A1, 104724, BioLegend).

### Co-immunoprecipitation and western blot

Cells were lysed in EBC buffer (50 mM Tris pH 7.5, 120 mM NaCl, 0.5% NP-40) supplemented with protease inhibitors (A32963, ThermoFisher) and phosphatase inhibitors (APExBIO, #K1015). The protein concentration was measured using the Bio-Rad protein assay reagent on a Beckman Coulter DU-800 spectrophotometer. The lysates were then resolved by SDS-PAGE and immunoblotted with indicated antibodies. For immunoprecipitation, 0.5 to 1 mg lysates were incubated with the appropriate antibody or beads overnight or for 4 hours at 4 °C. Immuno-complexes were washed four times with NETN buffer (20 mM Tris, pH 8.0, 100 mM NaCl, 1 mM EDTA and 0.5% NP-40) before being resolved by SDS-PAGE and immunoblotted for indicated proteins. For immunoblotting analysis, the primary antibodies were diluted in 1% BSA in TBST, and the secondary antibodies were diluted in 5% non-fat milk.

### In vitro methylation assays

GST-cGAS proteins were purified from BL-21 *E. Coli* were used for the in vitro methylation of cGAS. Briefly, the bacterial expression construct pGEX-GST-cGAS vector was transfected into BL-21 *E. Coli*, and the protein was induced by 100 µM of IPTG at 18 °C for 12 hours. Then, the bacteria was lysed in PBS with protease inhibitor with sonication for 15 minutes, then centrifuged at 10,000 × *g* for 30 minutes at 4 °C. The supernatant was incubated with Glutathione Sepharose 4B beads (GE Healthcare, GE17-7056-05) for 3 hours, washed with PBS four times. Finally, 10 µg GST-tagged proteins were incubated with S-(5’-Adenosyl)-L-methionine chloride (SAM), a methyl donor (Cayman, #13965, 1 mM final concentration), with or without 1 µg PRMT1 protein (Active motif, #31411) at 30 °C for 1.5 hours, followed by SDS-PAGE.

### Measurement of cGAMP level

The cGAMP level was measured using 2’3’-cGAMP ELISA kit (Cayman, #501700) following the manufacturer’s manual. Briefly, cells were lysed using the M-PER^tm^ Mammalian Protein Extraction Reagent (ThermoFisher, #78503), and 100 µL cell lysates were used for analysis. The OD450 were measured for calculation of cGAMP level, and finally normalized with protein concentration.

### Cell stimulation with DNA or RNA

To activate cellular DNA or RNA sensing pathways, cells were transfected with either HT-DNA (Sigma, D6898), or ISD45 (TACAGATCTACTAGTGATCTATGACTGATCTGTACATGATCTACA-3’, synthesized from IDT) or Poly(I:C) (InvivoGen, #tlrl-pic). Briefly, the HT-DNA, ISD45, and Poly(I:C) were transfected with PEI (Polysciences, #23966) for 12 hours.

### Biotin-pulldown assay

Cell lysates derived from HEK293T cells that express either wild-type cGAS or its mutants were mixed with biotin-ISD for 3 hours at 4 °C, followed by further incubation with 10 μL of streptavidin Agarose beads (Pierce, #20353) for 1 hour at 4 °C. Then, the beads were washed four times with wash buffer (50 mM Tris-HCl, pH 7.5, 100 mM NaCl, 10% glycerol, and 0.5% NP-40) and denatured by adding SDS sample buffer, followed by SDS-PAGE and immunoblotting.

### RNA-sequencing and bioinformatics analyses

Total RNAs were extracted from CT26 cells using QIAsheredder (Qiagen, #79656) and Qiagen RNeasy mini kit (Qiagen. #74106). Library preparation (Roche, Kapa mRNAseq Hyper prep) and sequencing analysis (Illumina NS500 Paired-end 2 × 150 bp) were performed at the Molecular Biology Core facility at Dana-Farber Cancer Institute. The data were aligned to mm10 by Salmon 1.4.0^62^ using the default parameters. The DEGs were calculated using DESeq2^63^. For GSEA analysis, we used the GSEA tool v.4.2.2^64^, with the MSigDB v.7.1 Hallmarks gene sets collection and the ‘classic’ method for calculating enrichment scores.

### RT-qPCR

Total RNAs were extracted using Qiagen RNeasy mini kit (Qiagen. #74106) and reversed transcripted into cDNA using iScript™ Reverse Transcription Supermix (Bio-Rad, # 1708841). RT-qPCR was performed with SYBR Select Master Mix (ThermoFisher, #4472908) using indicated primers.

### Treatment of wild-type mice with PRMT1 inhibitors

Six-weeks old BALB/c female mice (Taconic) were treated with MS023 (80 mg/kg body weight, by i.p. injection), GSK3368715 (80 mg/kg body weight, by gastric gavage), or respective vehicle, one dose daily for 21 days. MS023 (SelleckChem, #S8112) was dissolved in NMP (Sigma, #328643), and then sequentially diluted with 20% Captisol (SelleckChem, #S4592), PEG-400 (Sigma, PX1286B-2) and saline, with a final ratio of 5:20:20:55 (NMP/20% Captisol/PEG-400/saline, v/v/v/v)^65^. GSK3368715 (SelleckChem, #S8858) was dissolved in ddH_2_O. Then, tissues and organs were collected and analyzed by immunoblotting.

### In vivo experimental therapy in syngeneic mice tumor models

The study is compliant with all relevant ethical regulations regarding animal research. Animal experiments were approved by Dana-Farber Cancer Institute Institutional Animal Care and Use Committee (IACUC; protocol number 04–047) or Beth Israel Deaconess Medical Center (BIDMC) Institutional Animal Care and Use Committee (IACUC: Protocol #043–2019), and performed in accordance with guidelines established by NIH Guide for the care and use of laboratory animals. Briefly, a total of 2 × 10^5^ CT26 or MC38 cells in 100 μL HBSS saline buffer were injected subcutaneously into 6 weeks old BALB/c or C57BL/6 female mice (Jackson Lab or Taconic). Tumor sizes were measured every three days after tumor cells implantation, and tumor volume was calculated by L × W^2^ × 0.5 (L: length, W. width). On day 7 after tumor engraftment, mice were pooled and randomly divided into experimental groups. For experiment #1, mice were grouped into 4 groups: (1) CT26-cGAS^+/+^ treated with vehicle; (2) CT26-cGAS^+/+^ treated with MS023; (3) CT26-cGAS^−/−^ treated with vehicle; (4) CT26-mccGAS^−/−^ treated with MS023. For experiment #2 (CT26 cells) and 3 (MC38 cells), mice were grouped into 4 groups and treated with: (1) control antibody and vehicle; (2) anti-PD-1 mAb and vehicle; (3) control antibody and MS023; (4) anti-PD-1 mAb and MS023. For experiment #4, mice were grouped into 4 groups: (1) CT26-cGAS^+/+^ treated with vehicle and control antibody; (2) CT26-cGAS^+/+^ treated with MS023 and anti-PD-1 mAb; (3) CT26-cGAS^−/−^ treated with vehicle and control antibody; (4) CT26-cGAS^−/−^ treated with MS023 and anti-PD-1 mAb. The control and anti-PD-1 mAb (clone 1A12) were prepared in HBSS saline buffer in a final working solution of 1 mg/mL, and intraperitoneal injected with 200 μg/mouse in 200 μL HBSS saline buffer every three days for a total of 4 injections. MS023 treatment was given by daily intraperitoneally injection with 80 mg/kg body weight (in 5% NMP, 20% Captisol, 20% PEG-400 in saline) for a total of 14 injections. For survival studies, animals were monitored for tumor volumes for up to 92 days, until tumor volume exceeded 1000 mm^3^, or the diameter of tumor exceeded 1.5 cm, or until the tumor became ulcerated with ulcer diameter reaching 0.5 cm with a diameter of tumor exceeded 1.0 cm. Statistical analysis was conducted using the GraphPad Prism 8 software (GraphPad Software). Kaplan–Meier curves and corresponding Gehan-Breslow-Wilcoxon tests were used to evaluate statistical differences between groups in survival studies.

### Tumor-infiltrating lymphocytes analysis

Fourteen days after the treatment of CT26 tumors with indicated compounds, tumor-infiltrating lymphocytes (TILs) were isolated and stained as previous described. Briefly, tumor were dissected and chopped into fine pieces, digested in the dissociation buffer (RPMI1640, 5% FBS, 1 mg/mL Collagenase IV (Sigma, C5138), and 200 U/mL DNase I (Roch, #04536282001) at 37 °C for 20 minutes. Then, the digests were filtered with a 70 µm cell strainer (Falcon, #352350), and spun at 1900 RPM for 10 minutes. The cells were further treated with Red Cell Lysis Buffer (Sigma, #R7757) at RT for 1 minute, and stopped by adding R10 buffer (RPMI1640, 10% FBS), followed by spin at 1200 RPM for 5 minutes. The cell pellets were resuspended in 2 mL of 40% Percoll (GE Healthcare, #17-0891-01) in PBS, topped on 2 mL of 70% Percoll, and spun at 2000 RPM for 30 minutes. Finally, the immune cell layer between the two Percoll interphase was collected and washed once with R10 buffer and spun at 1500 RPM for 5 minutes. The purified immune cells were resuspended in MACS buffer (HBSS, no Ca2^+^, no Mg2^+^, Gibco, #14175-095, 1% FBS, 2 mM EDTA).

For FACS staining, 100 µL cells in MACS buffer were blocked by 1 µg Fc blocker ((anti-mouse CD16/CD32 antibody, Clone 2.4G2, Bio X Cell) for 15 minutes, then stained with Live-Dead NIR (Invitrogen, #L10119) and individual antibodies at RT for 40 minutes, washed with MACS buffer. Then, the stained cells were further fixed and permeabilizated using the eBioscience Foxp3/Transcription Factor Staining Buffer (ThermoFisher, #00-5533-00), followed by staining with Foxp3 and GramB antibodies at RT for 40 minutes, and washed with MACS buffer. Stained cells were analyzed by multicolor flow cytometry (BD LSR Fortessa X-20) and the FACS data were analyzed using FlowJo_V10.6.1 software (Tree Star). T cells gating strategy: gate cells exclude dead cells and debris based on cells size, then gate live cells based on Live-Dead NIR negative cells, then gate CD45^+^ cells, then gate CD45^+^CD3^+^ cells, then gate CD45^+^CD3^+^CD8^+^ cells and CD45^+^CD3^+^CD4^+^ cells. Macrophage gating strategy: gate cells exclude dead cells and debris based on cell size, then gate live cells based on Live-Dead NIR negative cells, then gate CD45^+^ cells, then gate CD45^+^CD11b^+^ cells.

### Transcripts and survival analyses

PRMT1 transcripts across all cancer types, the association between PRMT1 expression and effector T cell signature, as well as the association between cGAS and PD-L1 expression were analyzed using the gene expression profiling interactive analysis v2 (GEPIA2)^66^ (http://gepia2.cancer-pku.cn). The association between PRMT1 expression and immune cell infiltration in tumors was analyzed using TIMER2^67^ (http://timer.cistrome.org/). The association between cGAS and PD-L1 expression in breast cancer patients was analyzed using cBioPortal^68,69^ (https://www.cbioportal.org). The customized genomic analysis was based on The Cancer Genome Atlas (TCGA) data (https://cancergenome.nih.gov/). The expression data of cGAS and PD-L1 in a panel of BRCA cell lines were obtained from GEO dataset GSE73526^70^. The data for PRMT1 expression and survival of patients with bladder cancer after PD-L1 antibody treatment were generated using the TIDE tool^71^ (http://tide.dfci.harvard.edu) and the source data are based on the Mariathasan2018_PDL1cohort^72^. The association between PRMT1 expression level and T cell dysfunction in multiple cancer types was generated using the TIDE tool and the source data are derived from data in TCGA and PRECOG^73^.

### Statistics and reproducibility

All quantitative data were presented as the Mean ± S.D., as indicated by at least three independent experiments or biological replicates unless otherwise stated. Statistical analyses were performed using GraphPad Prism 8 and Excel unless indicated otherwise. The statistical tests and *P* values were described in the figure legend for each experiment. All *t*-tests were two-sided. *P* < 0.05 was considered statistically significant. All data shown are representative of two or more independent experiments with similar results, unless indicated otherwise.

### Reporting summary

Further information on research design is available in the Nature Portfolio Reporting Summary linked to this article.

## Supplementary information

Supplementary Information

Reporting Summary

## Data Availability

Data supporting the findings of this work are available within the paper and the Supplementary Information. RNA-seq data used to support the present study have been deposited in the Gene Expression Omnibus with an access number of GSE203466. Further information and requests for resources and reagents should be directed to the lead author, W.W. Source data are provided with the paper.

## Source data

Source Data
