# Supplementary material for: PRMT1 mediated methylation of cGAS suppresses anti-tumor immunity
Source: Nat Commun. 2023 May 17;14:2806. doi: 10.1038/s41467-023-38443-3 (PMC10188589; doi:10.1038/s41467-023-38443-3)

Supplementary Information for

PRMT1 mediated methylation of cGAS suppresses anti-tumor immunity

Jing Liu[1, 2, 3, 4, 9], Xia Bu[5, 9], Chen Chu[6, 7], Xiaoming Dai[1], John M. Asara[8], Piotr Sicinski[6, 7], Gordon J. Freeman[5, *] and Wenyi Wei[1, *]

[*]Corresponding author

gordon_freeman@dfci.harvard.edu (G.F.)

wwei2@bidmc.harvard.edu (W.W.).

The PDF file includes:

Supplementary Figure 1 to 7
Supplementary Table 1 and 2

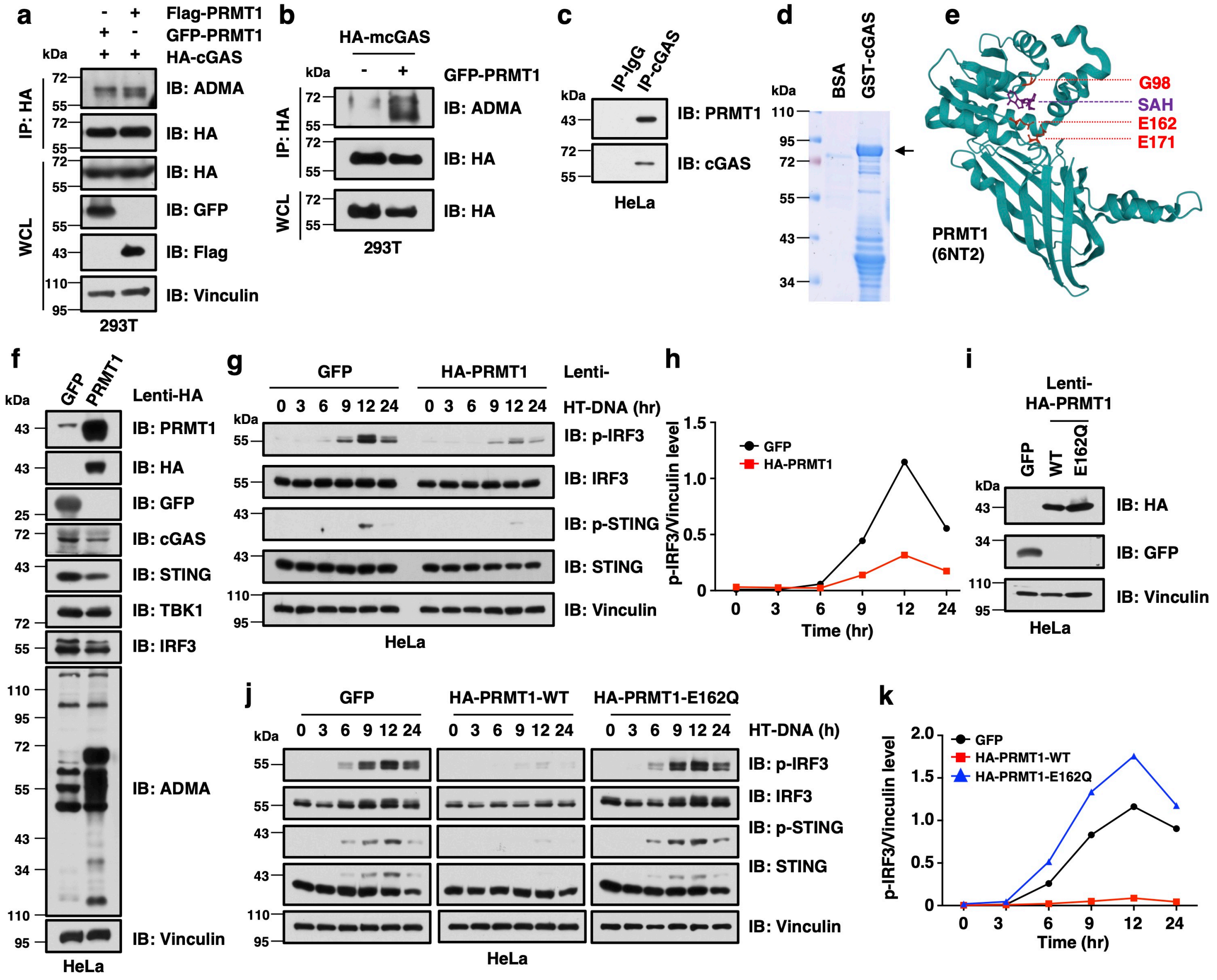

Supplementary Figure 1. PRMT1 methylates cGAS and inhibits the cGAS/STING signaling.

a. PRMT1 methylates human cGAS. Immunoblot analysis of HA-immunoprecipitants and WCL derived from HEK293T ectopically transfection of HA-GAS and GFP-PRMT1 or Flag-PRMT1.

b. PRMT1 methylates mouse cGAS. Immunoblot analysis of HA-immunoprecipitants and WCL derived from HEK293T ectopically transfection of GFP-PRMT1 and HA-mcGAS.

c. Endogenous PRMT1 binds with cGAS. Immunoblot analysis of cGAS or normal IgG-immunoprecipitants derived from HeLa cells.

d. Gel staining for GST-cGAS protein purified from E. Coli. Arrow indicates the band of GST-cGAS.

e. A schematic diagram shows the critical residues for the arginine methylase activity of PRMT1. The graphic is derived from the PRMT1 structure (6NT2). G98: glycine 98, E162: glutamic

acid 162, E171: glutamic acid 171, SAH: A- adenosylhomocysteine.

f. Immunoblot analysis of HeLa stable cell lines expressing either GFP or HA-PRMT1. HeLa cells were infected with GFP or HA-PRMT1 lentivirus, selected with hygromycin for 3 days, then harvested for immunoblot analysis.

g. PRMT1 overexpression suppresses DNA sensing. Immunoblot analysis of HeLa stable cell lines expressing either GFP or HA-PRMT1 as in **e** after stimulating with HT-DNA for indicated times.

h. Quantification for the ratio of p-IRF3 to Vinculin in **f**.

i. Immunoblot analysis of HeLa stable cell lines expressing either GFP or HA-PRMT1, or HA-PRMT1-E162Q. HeLa cells were infected with GFP or HA-PRMT1 lentivirus, selected with hygromycin for 3 days, then harvested for immunoblot analysis.

j. PRMT1 suppresses DNA sensing in a time- and catalytic-dependent manner. HeLa stable cell lines expressing either GFP, HA-PRMT1-WT, or HA-PRMT1-E162Q as in h were stimulated with HT-DNA for indicated time, then harvested for immunoblot analysis.

k. Quantification for the ratio of p-IRF3 to Vinculin in **i**.

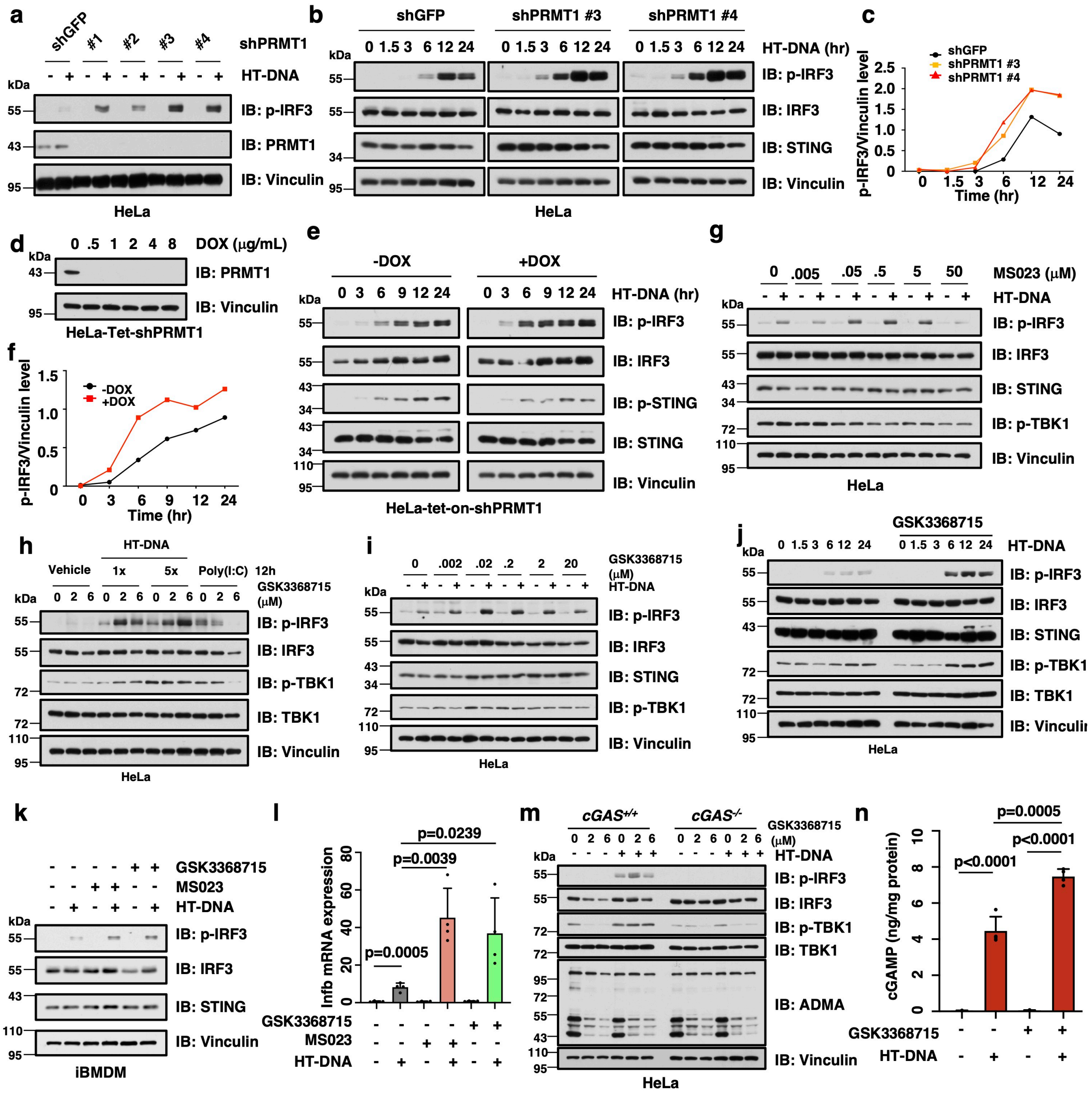

Supplementary Figure 2. Genetic ablation or pharmaceutical inhibition of PRMT1 activates the cGAS/STING DNA sensing signaling pathway.

a. Depletion of *PRMT1* activates cGAS/STING DNA sensing signaling. HeLa cells were infected with indicated shRNA lentivirus, selected with 1 μg/mL of puromycin for 3 days. The stable cell lines were stimulated with 1 μg/mL of HT-DNA for 12 hours and harvested for immunoblot analysis.

b. Depletion of *PRMT1* activates the cGAS/STING DNA sensing signaling in a time-dependent manner. HeLa stable cell lines after depletion of *PRMT1* as in fig. S2A were stimulated with

1 μg/mL of HT-DNA for indicated time points and harvested for immunoblot analysis.

c. Quantification for the ratio of p-IRF3 to Vinculin in b.

d. Immunoblot analysis of HeLa-tet-sh*PRMT1* stable cell lines after treatment with indicated doses of DOX for 3 days.

e. DOX-induced depletion of *PRMT1* activates cGAS/STING DNA sensing signaling in a time-dependent manner. Immunoblot analysis of HeLa-tet-sh*PRMT1* cells with or without DOX treatment of 3 days, followed by stimulating with 1 μg/mL of HT-DNA for 12 hours and harvested for immunoblot analysis.

f. Quantification for the ratio of p-IRF3 to Vinculin in e.

g. PRMT1 inhibitor MS023 activates the cGAS/STING DNA sensing signaling in a dose-dependent manner. HeLa cells after treatment with indicated doses of PRMT1 inhibitor MS023 for 48 hours, followed by stimulation with 1 μg/mL of HT-DNA for 12 hours and harvesting for immunoblot analysis.

h. PRMT1 inhibition activates DNA sensing signaling but not RNA sensing signaling. HeLa cells were treated with 2 or 6 μM of PRMT1 inhibitor GSK3368715 for 48 hours, stimulated with HT-DNA (1x: 1 μg/mL; 5x: 5 μg/mL) or Poly(I:C) for 12 hours, then harvested for immunoblot analysis.

i. PRMT1 inhibitor GSK3368715 activates the cGAS/STING DNA sensing signaling in a dose-dependent manner. HeLa cells after treatment with indicated doses of GSK3368715 for 48 hours, followed by stimulation with 1 μg/mL of HT-DNA for 12 hours and harvested for immunoblot analysis.

j. PRMT1 inhibitor GSK3368715 activates the cGAS/STING signaling with different dose of DNA challenge. HeLa cells after treatment with indicated 2 μM of GSK3368715 for 48 hours, followed by stimulation with 1 μg/mL of HT-DNA for indicated hours and harvested for immunoblot analysis.

k. PRMT1 inhibitors activate the cGAS/STING DNA sensing signaling in iBMDM cells. The iBMDM cells were treated with PRMT1 inhibitor for 24 hours, followed by stimulation with 1 μg/mL of HT-DNA for 3 hours and harvested for immunoblot analysis.

l. PRMT1 inhibition elevates interferon transcription in iBMDM cells. The iBMDM cells were treated with PRMT1 inhibitor for 24 hours, followed by stimulation with 1 μg/mL of HT-DNA for 3 hours and harvested for RT-qPCR analysis. n=4. Data are presented as mean values ±

SD. Two tailed unpaired Student *t*-test.

m. PRMT1 inhibitor GSK3368715 activates the cGAS/STING signaling in a cGAS-dependent manner. HeLa-cGAS-WT or cGAS-KO cells were treated with 2 or 6 μM of PRMT1 inhibitor GSK3368715 for 48 hours, followed by stimulation with 1 μg/mL of HT-DNA for 12 hours, and harvested for immunoblot analysis.

n. PRMT1 inhibitor GSK3368715 increases DNA-stimulated cGAMP production in a cGAS-dependent manner. HeLa-cGAS-WT or cGAS-KO cells were treated with 2 μM of GSK3368715 for 48 hours, followed by stimulation with 1 μg/mL of HT-DNA for 12 hours, and harvested for ELISA analysis of cGAMP abundance. n=4. Data are presented as mean values ± SD. Two tailed unpaired Student *t*-test.

Source data are provided as a Source Data file.

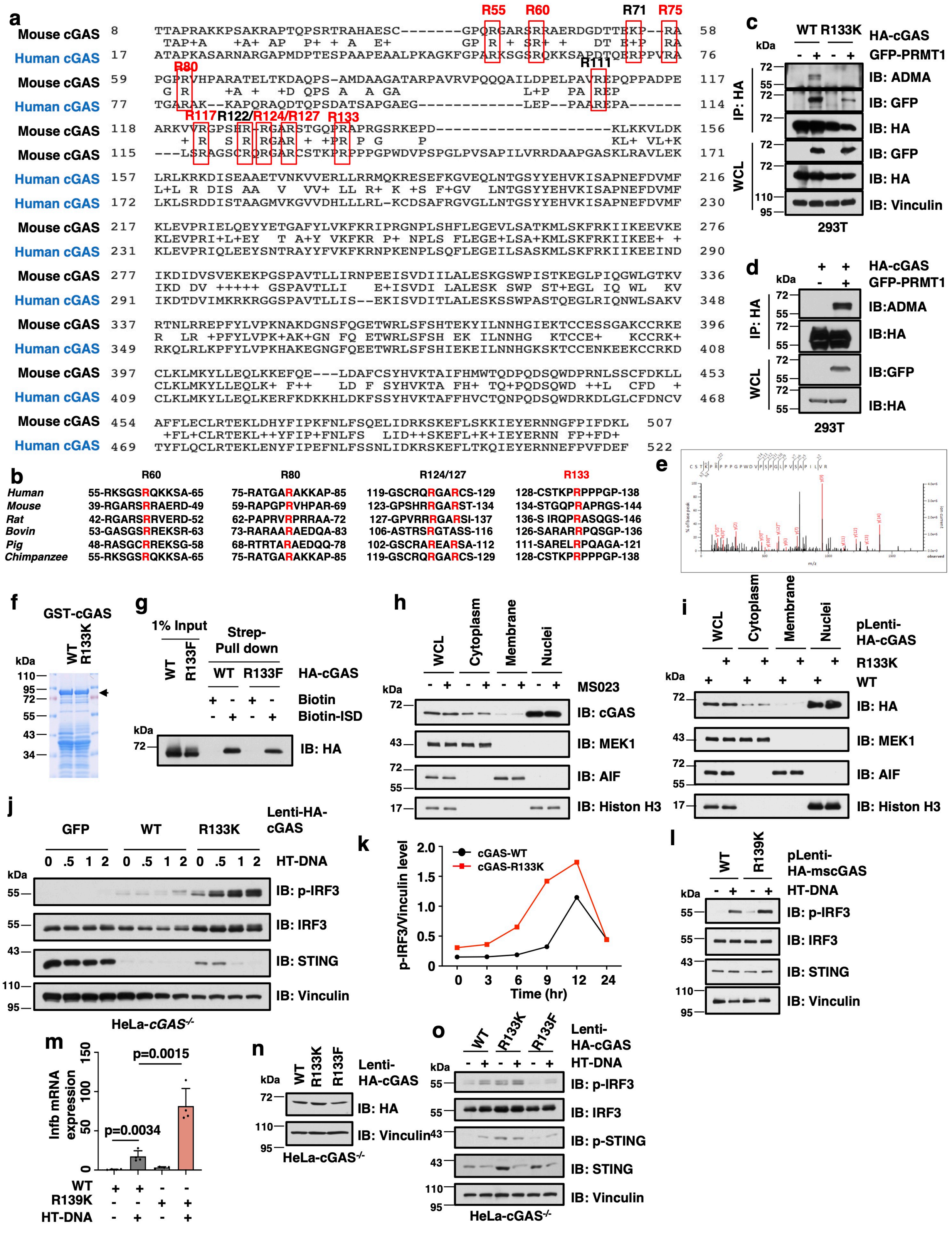
a
R55 R60 R71 R75
Mouse cGAS 8 TTAPRAKKPSAKRAPTQPSRTRAHAESC--------GPQRGARSRRAERDGDTTEKP--RA 58
TAP+A +A+ AP P+ + A E+ GP R SR+ + DT E+P RA
Human cGAS 17 ATAPKASARNARGAPMDPTESPAAEAALPKAGKFGPARKSGSRQKKSAPDTQERPPVRA 76
R80 R111
Mouse cGAS 59 PGPRVHPARATELTKDAQPS-AMDAAGATARPAVRVPQQQAILDPELPAVREPQPPADPE 117
G R +A + +D QPS A A GA L+P PA REP
Human cGAS 77 TGARAK--KAPQRAQDTQPSDATSAPGAEG------------LEP--PAAREPA------ 114
R117 R122/R124/R127 R133
Mouse cGAS 118 ARKVVRGPSHRGARSTGQPRAPRGSRKEPD--------------------KLKKVLDK 156
+ R S R RGAR + +PR P G P KL+ VL+K
Mouse cGAS 115 ---LSRAGSCRQRGARCSTKPRPPPGPWDVPSPGLPVSAPILVRRDAAPGASKLRAVLEK 171
Human cGAS 157 LRLKRKDISEAAETVNKVVERLLRRMQKRESEFKGVEQLNTGSYYEHVKISAPNEFDVMF 216
L+L R DIS AA V VV+ LL R+ K +S F+GV LNTGSYYEHVKISAPNEFDVMF
Human cGAS 172 LKLSRDDISTAAGMVKGVVDHLLLRL-KCDSAFRGVGLLNTGSYYEHVKISAPNEFDVMF 230
Mouse cGAS 217 KLEVPRIELQEYYETGAFYLVKFKRIPRGNPLSHFLEGEVLSATKMLSKFRKIIKEEVKE 276
KLEVPRI+L+EY T A+Y VKFKR P+ NPLS FLEGE+LSA+KMLSKFRKIIKEE+ +
Human cGAS 231 KLEVPRIQLEEYSNTRAYYFVKFKRNPKENPLSQFLEGEILSASKMLSKFRKIIKEEIND 290
Mouse cGAS 277 IKDIDVSVEKEKPGSPAVTLLIRNPEEISVDIILALESKGSWPISTKEGLPIQGWLGTKV 336
IKD DV +++++ GSPAVTLLI E+ISVDI LALESK SWP ST+EGL IQ WL KV
Human cGAS 291 IKDTDVIMKRKRGGSPAVTLLIS--EKISVDITLALESKSSWPASTQEGLRIQNWLSAKV 348
Mouse cGAS 337 RTNLRREPFYLVPKNAKDGNSFQGETWRLSFSHTEKYILNNHGIEKTCCESSGAKCCRKE 396
R LR +PFYLVPK+AK+GN FQ ETWRLSFSH EK ILNNHG KTCCE+ KCCRK+
Human cGAS 349 RKQLRLKPFYLVPKHAKEGNGFQEETWRLSFSHIEKEILNNHGKSKTCCENKEEKCCRKD 408
Mouse cGAS 397 CLKLMKYLLEQLKKEFQE---LDAFCSYHVKTAIFHMWTQDPQDSQWDPRNLSSCFDKLL 453
CLKLMKYLLEQLK+ F++ LD F SYHVKTA FH+ TQ+PQDSQWD ++L CFD +
Human cGAS 409 CLKLMKYLLEQLKERFKDKKHLDKFSSYHVKTAFFHVCTQNPQDSQWDRKDLGLCFDNCV 468
Mouse cGAS 454 AFFLECLRTEKLDHYFIPKFNLFSQELIDRKSKEFLSKKIEYERNNGFPIFDKL 507
+FL+CLRTEKL++YFIP+FNLFS LID++SKEFL+K+IEYERNN FP+FD+
Human cGAS 469 TYFLQCLRTEKLENYFIPEFNLFSSNLIDKRSKEFLTKQIEYERNNEFPVFDEF 522
b
R60 R80 R124/127 R133
Human 55-RKSGSRQKKSA-65 75-RATGARAKKAP-85 119-GSCRQRGARCS-129 128-CSTKPRPPPGP-138
Mouse 39-RGARSRRAERD-49 59-RAPGPRVHPAR-69 123-GPSHRRGARST-134 134-STGQPRAPRGS-144
Rat 42-RGARSRRVERD-52 62-PAPRVRPRRAA-72 127-GPVRRRGARSI-137 136-S IRQPRASQGS-146
Bovin 53-GASGSRREKSR-63 73-RARAARAEDQA-83 106-ASTRSRGTASS-116 126-SARARRPQSGP-136
Pig 48-RASGCRREKSG-58 68-RTRTARAEDQQ-78 102-GSCRAREARSA-112 111-SARELRPQAGA-121
Chimpanzee 55-RKSGSRQKKSA-65 75-RATGARAKKAP-85 119-GSCRQRGARCS-129 128-CSTKPRPPPGP-138
c
WT R133K HA-cGAS
kDa - + - + GFP-PRMT1
IP: HA
IB: ADMA
IB: GFP
IB: HA
WCL
IB: GFP
IB: HA
IB: Vinculin
293T
d
+ + HA-cGAS
- + GFP-PRMT1
IP: HA
IB:ADMA
IB:HA
WCL
IB:GFP
IB:HA
293T
e
C S T K P R P P P G P W D V P S P G L P V S A P I L V R
f
GST-cGAS
WT R133K
kDa
110 95 72 55 43 34
g
1% Input
WT R133F
Strep-Pull down
WT R133F HA-cGAS
+ - + - Biotin
- + - + Biotin-ISD
kDa 72
IB: HA
h
WCL Cytoplasm Membrane Nuclei
- + - + - + - + MS023
IB: cGAS
IB: MEK1
IB: AIF
IB: Histon H3
i
WCL Cytoplasm Membrane Nuclei
pLenti-HA-cGAS
+ + + + R133K
+ + + + WT
IB: HA
IB: MEK1
IB: AIF
IB: Histon H3
j
GFP WT R133K Lenti-HA-cGAS
0 .5 1 2 0 .5 1 2 0 .5 1 2 HT-DNA
IB: p-IRF3
IB: IRF3
IB: STING
IB: Vinculin
HeLa-cGAS-/-
k
cGAS-WT
cGAS-R133K
p-IRF3/Vinculin level
Time (hr)
0 3 6 9 12 24
0 0.5 1.0 1.5 2.0
l
WT R139K pLenti-HA-mscGAS
- + - + HT-DNA
IB: p-IRF3
IB: IRF3
IB: STING
IB: Vinculin
m
Infb mRNA expression
p=0.0015
p=0.0034
0 50 100 150
WT + + - -
R139K - - + +
HT-DNA - + - +
n
WT R133K R133F Lenti-HA-cGAS
IB: HA
IB: Vinculin
HeLa-cGAS-/-
o
WT R133K R133F Lenti-HA-cGAS
- + - + - + HT-DNA
IB: p-IRF3
IB: IRF3
IB: p-STING
IB: STING
IB: Vinculin
HeLa-cGAS-/-

Supplementary Figure 3. Identification of the arginine methylation site in cGAS that is mediated by PRMT1.

a. Alignment of human and mouse cGAS. The conserved arginine residues in the N-terminus of human and mouse cGAS were boxed and highlighted.

b. Alignment of the conserved arginine residues in the N-terminus of cGAS among different spices.

c. The R133K mutation abolished PRMT1-mediated asymmetric demethylation (ADMA) on cGAS. Immunoblot analysis of the HA immunoprecipitate and WCL derived from HEK293T cells that expressed GFP-PRMTs and indicated HA-cGAS mutants.

d. Immunoblot analysis of the HA immunoprecipitate and WCL derived from HEK293T cells that expressed GFP-PRMTs and HA-cGAS.

e. Mass spectrometry analysis shows the arginine methylation of R133 in cGAS. HA immunoprecipitated from **d** were separated by SDS-PAGE, and collected for mass spectrometry analysis.

f. Gel staining for GST-cGAS-WT and GST-cGAS-R133K proteins purified from *E. Coli*.

g. The arginine methylation mimic mutant R133F does not affect the DNA binding affinity to DNA. Immunoblot analysis of streptavidin-biotin-ISD pulldown products and WCL derived from HEK293T that were ectopically transfection with either HA-GAS-WT or HA-GAS-R133F mutant.

h. Immunoblot analysis of subcellular fractions derived from HeLa cells with or without pretreated with MS023.

i. Immunoblot analysis of subcellular fractions derived from HeLa cells that expressed with cGAS WT or R133K mutant.

j. Mutation of the arginine methylation R133 residue activates cGAS/DNA sensing signaling. HeLa-*cGAS*$^{-/-}$ cells were infected with either GFP, or HA-GAS-WT or HA-GAS-R133K mutant lentivirus and selected with hygromycin for 3 days. The stable cell lines were stimulated with indicated doses of HT-DNA for 12 hours, and harvested for immunoblot analysis.

k. Quantification for the ratio of p-IRF3 to Vinculin in Figure 3f.

l. Immunoblot analysis of iBMDM cells that expressed mouse GAS-WT or R139K mutants.

m. RT-qPCR analysis of iBMDM cells that expressed mouse GAS-WT or R139K mutants. n=4.

Data are presented as mean values ± SD.

n. Immunoblot analysis of HeLa stable cell lines that expressed HA-GAS-WT, HA-GAS-R133K or HA-GAS-R133F mutants.

o. Immunoblot analysis of HeLa stable cell lines that expressed HA-GAS-WT HA-GAS-R133K or HA-GAS-R133F mutants after stimulation with HT-DNA for 12 hours.

Source data are provided as a Source Data file.

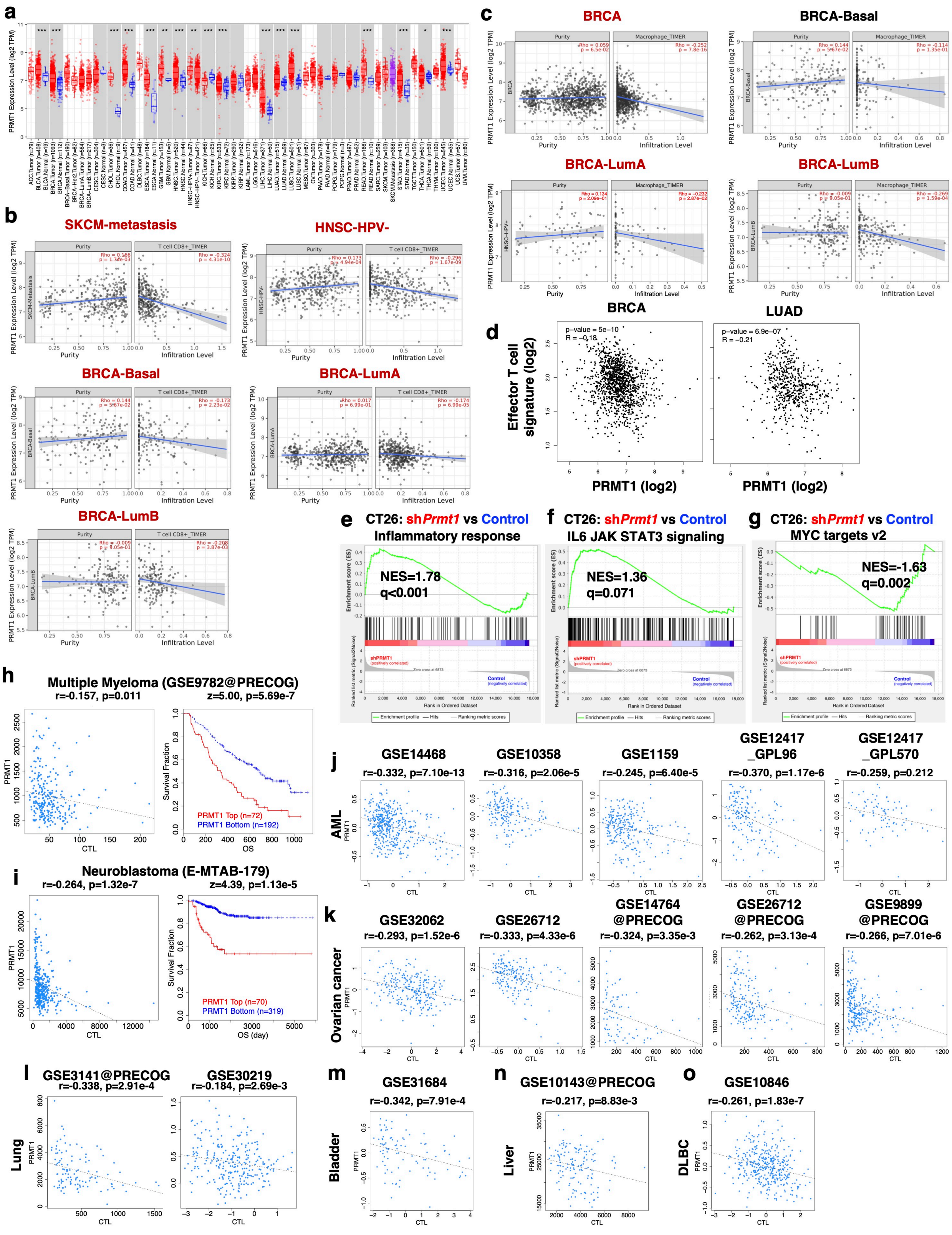
a
PRMT1 Expression Level (log2 TPM)
b
SKCM-metastasis
HNSC-HPV-
BRCA-Basal
BRCA-LumA
BRCA-LumB
Purity
T cell CD8+_TIMER
Infiltration Level
c
BRCA
BRCA-Basal
BRCA-LumA
BRCA-LumB
Macrophage_TIMER
d
BRCA
LUAD
p-value = 5e-10
R = -0.18
p-value = 6.9e-07
R = -0.21
Effector T cell signature (log2)
PRMT1 (log2)
e
CT26: shPrmt1 vs Control
Inflammatory response
NES=1.78
q<0.001
f
CT26: shPrmt1 vs Control
IL6 JAK STAT3 signaling
NES=1.36
q=0.071
g
CT26: shPrmt1 vs Control
MYC targets v2
NES=-1.63
q=0.002
h
Multiple Myeloma (GSE9782@PRECOG)
r=-0.157, p=0.011
z=5.00, p=5.69e-7
PRMT1 Top (n=72)
PRMT1 Bottom (n=192)
i
Neuroblastoma (E-MTAB-179)
r=-0.264, p=1.32e-7
z=4.39, p=1.13e-5
PRMT1 Top (n=70)
PRMT1 Bottom (n=319)
j
AML
GSE14468
r=-0.332, p=7.10e-13
GSE10358
r=-0.316, p=2.06e-5
GSE1159
r=-0.245, p=6.40e-5
GSE12417_GPL96
r=-0.370, p=1.17e-6
GSE12417_GPL570
r=-0.259, p=0.212
k
Ovarian cancer
GSE32062
r=-0.293, p=1.52e-6
GSE26712
r=-0.333, p=4.33e-6
GSE14764@PRECOG
r=-0.324, p=3.35e-3
GSE26712@PRECOG
r=-0.262, p=3.13e-4
GSE9899@PRECOG
r=-0.266, p=7.01e-6
l
Lung
GSE3141@PRECOG
r=-0.338, p=2.91e-4
GSE30219
r=-0.184, p=2.69e-3
m
Bladder
GSE31684
r=-0.342, p=7.91e-4
n
Liver
GSE10143@PRECOG
r=-0.217, p=8.83e-3
o
DLBC
GSE10846
r=-0.261, p=1.83e-7
CTL
PRMT1
Survival Fraction
OS
OS (day)

Supplementary Figure 4. PRMT1 overexpression predicts lower cancer immunity, lower CTL infiltration and worse prognosis in cancer patients.

a. Compared of PRMT1 expression levels in different cancers versus normal tissues by TIMER2. n is shown in figure. Data are presented as mean values ± SEM. The 25%, 50% and 75% are shown in the box plot.

b. Correlation analysis between PRMT1 expression and CD8+ T cells infiltration in multiple cancer types in TCGA by TIMER2. SKCM: skin cutaneous melanoma, HNSC: head and neck squamous cell carcinoma, BRCA: breast invasive carcinoma. The error bands represent the 95% confidence interval.

c. Correlation analysis between PRMT1 expression and macrophages infiltration in BRCA by TIMER2. Error band represents the 95% confidence interval.

d. Correlation analysis between PRMT1 expression with effect T cell signatures (CX3CR1, FGFBP2, and FCGR3A) in BRCA and LUAD by GEPIA2. LUAD: lung adenocarcinoma. Pearson correlation rho and p value are presented.

e. Gene set enrichment plots of the inflammatory response hallmarks after genetic ablation of *Prmt1* in CT26 cells. NES=1.78, q<0.001.

f. Gene set enrichment plots of the IL6-JAK-STAT3 hallmarks after genetic ablation of *Prmt1* in CT26 cells. NES=1.36, q<0.001.

g. Gene set enrichment plots of the MYC targets v2 hallmarks after genetic ablation of *Prmt1* in CT26 cells. NES=-1.63, q=0.002.

h. Correlation analysis between PRMT1 expression with CTL infiltration and survival in multiple myeloma (MM), derived from GSE9782 by TIDE.

i. Correlation analysis between PRMT1 expression with CTL infiltration and survival in neuroblastoma (NB) melanoma, derived from E-MTAB-179 by TIDE.

j. Correlation analysis between PRMT1 expression with CTL infiltration in acute myeloid leukemia (AML), derived from GSE14468, GSE10358, GSE1159, and GSE12417 by TIDE.

k. Correlation analysis between PRMT1 expression with CTL infiltration in ovary cancer (OVCA), derived from GSE32062, GSE26712, GSE14764, GSE26712, and GSE9899 by TIDE.

l. Correlation analysis between PRMT1 expression with CTL infiltration in lung cancer (LUAD), derived from GSE3141 and GSE30219 by TIDE.

m. Correlation analysis between PRMT1 expression with CTL infiltration in bladder cancer derived from GSE31684 by TIDE.

n. Correlation analysis between PRMT1 expression with CTL infiltration in liver cancer derived from GSE10143 by TIDE.

o. Correlation analysis between PRMT1 expression with CTL infiltration in diffuse large B cell lymphoma (DLBC) derived from GSE10846 by TIDE.

Spearman correlation rho and p value are presented for extended data figures 4b, c, h-o.

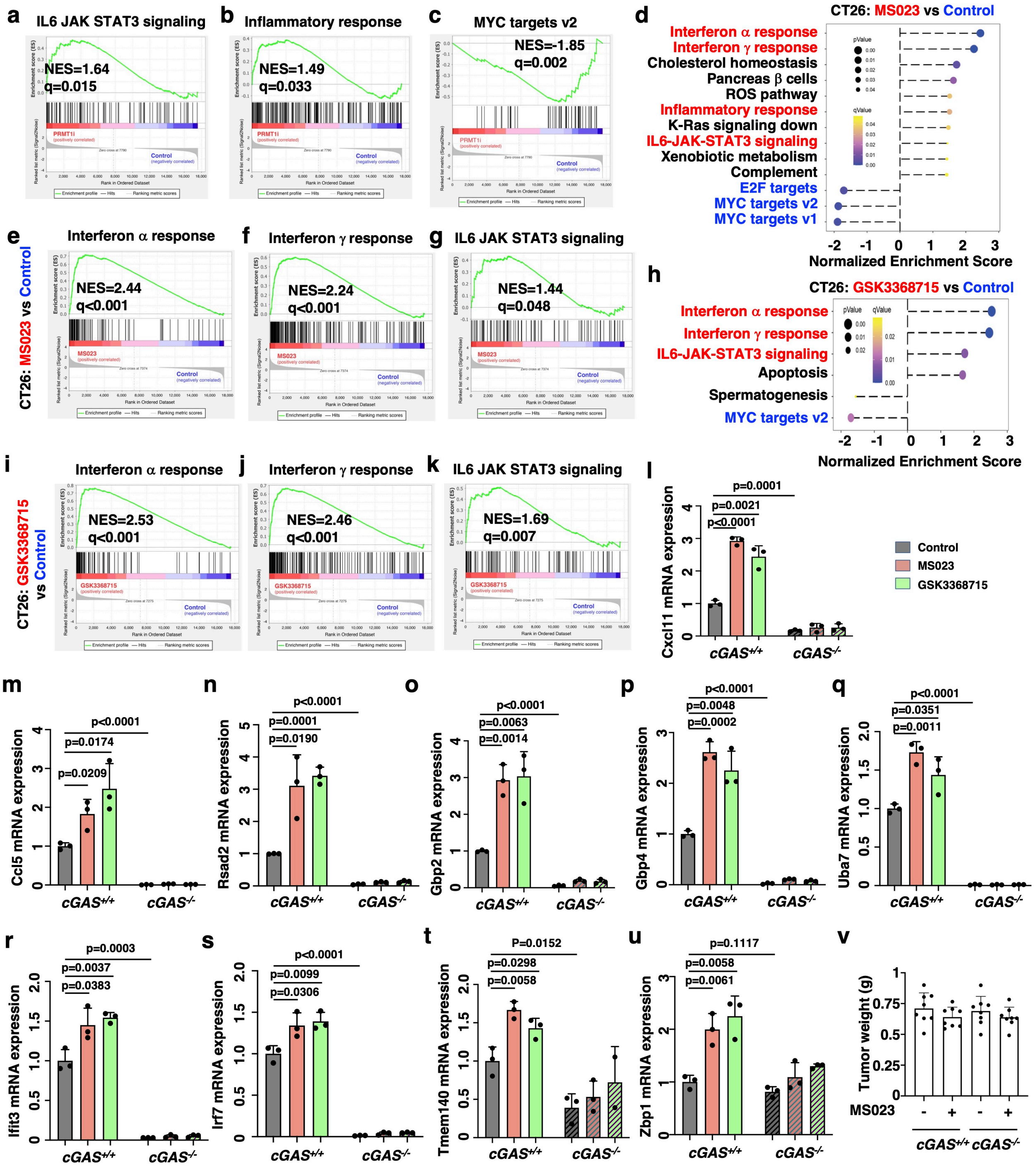

Supplementary Figure 5. Inhibition of PRMT1 triggers type I and II interferon gene expressions in a cGAS-dependent manner.

a. Gene set enrichment plots of the IL6-JAK-STAT3 hallmarks after treatment with PRMT1 inhibitors in CT26 cells. NES=1.64, q=0.015. PRMT1i represents a total of 6 samples with either MS023 or GSK3368715 treatment. n=3 replicates for each treatment group.

b. Gene set enrichment plots of the inflammatory response hallmarks after treatment with PRMT1 inhibitors in CT26 cells. NES=1.49, q=0.033.

c. Gene set enrichment plots of the MYC targets v2 hallmarks after treatment with PRMT1 inhibitors in CT26 cells. NES=-1.85, q=0.002.

d. PRMT1 inhibition activates the expression of type I and II interferon genes. CT26 mouse tumor cells were treated with DMSO, 6 μM of MS023 for 48 hours, then subjected to RNA-sequencing and GSEA analysis.

e-g. Gene set enrichment plots of the IFN-α response (e, NES=2.44, q<0.001), IFN-γ (f, NES=2.24, q<0.001) response, and IL6-JAK-STAT3 (g, NES=1.44, q=0.048) after treatment with MS023 in CT26 cells.

h. PRMT1 inhibition activates the expression of type I and II interferon genes. CT26 mouse tumor cells were treated with DMSO, 6 μM of GSK3368715 for 48 hours, then subjected to RNA-sequencing and GSEA analysis.

i-k. Gene set enrichment plots of the IFN-α response (i, NES=2.53, q<0.001), IFN-γ (j, NES=2.46, q<0.001) response, and IL6-JAK-STAT3 (k, NES=1.69, q=0.007) after treatment with MS023 in CT26 cells.

l-u. RT-qPCR analysis of Cxcl11 (l), Ccl5 (m), Rsad2 (n), Gbp2 (o), Gbp4 (p), Uba7 (q), Ifit3 (r), Irf7 (s), Tmem140 (t), and Zbp1 (u) in CT26-cGAS-WT and cGAS-KO cells after treatment with 6 μM of MS023 or GSK3368715 for 48 hours. n=4. Data are presented as mean values ± SD.

v. The weight of CT26 cells-derived tumor as in Figure 5j. n=8. Data are presented as mean values ± SD.

Source data are provided as a Source Data file.

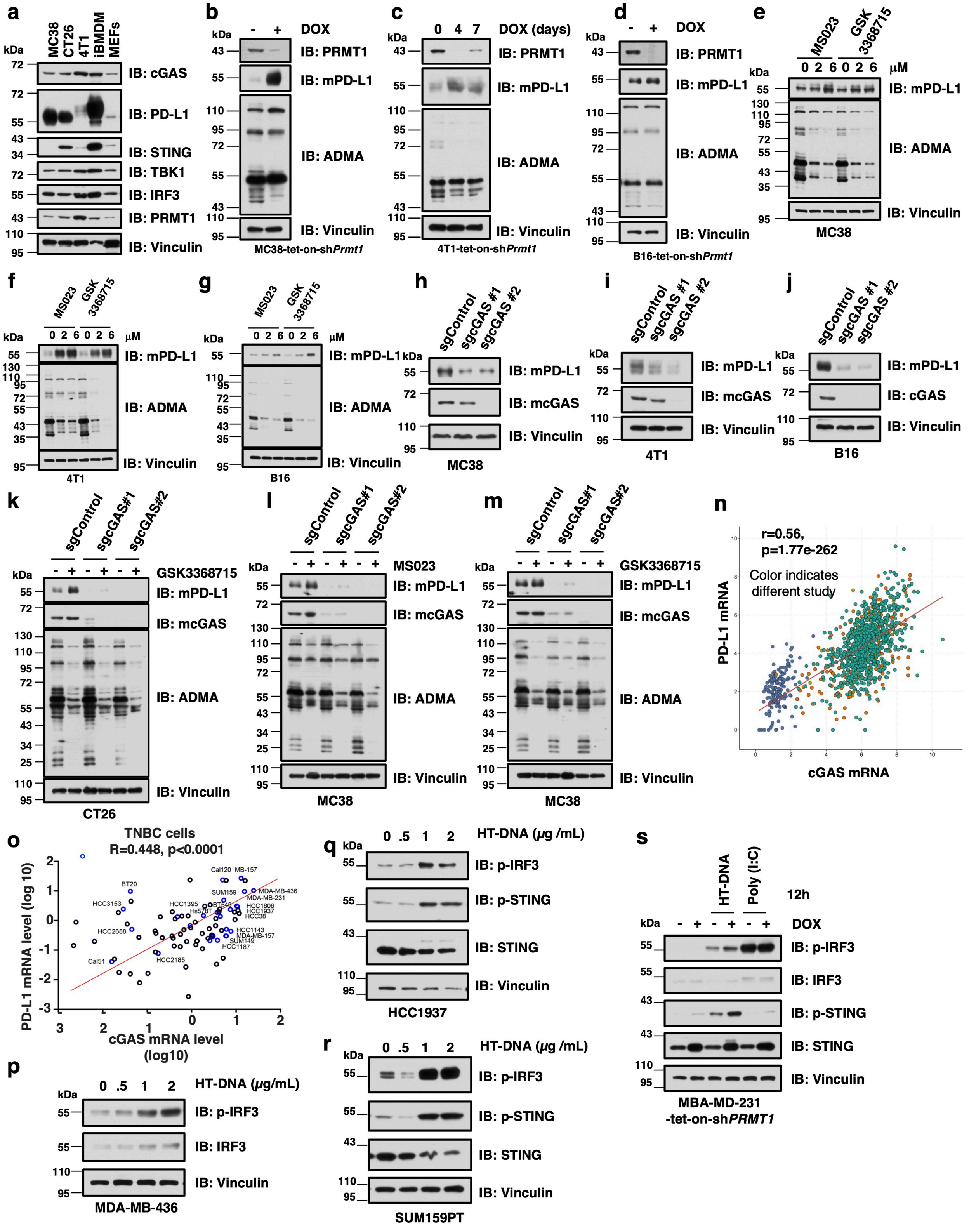
a
MC38 CT26 4T1 iBMDM MEFs
IB: cGAS
IB: PD-L1
IB: STING
IB: TBK1
IB: IRF3
IB: PRMT1
IB: Vinculin
b
DOX
IB: PRMT1
IB: mPD-L1
IB: ADMA
IB: Vinculin
MC38-tet-on-shPrmt1
c
DOX (days)
IB: PRMT1
IB: mPD-L1
IB: ADMA
IB: Vinculin
4T1-tet-on-shPrmt1
d
DOX
IB: PRMT1
IB: mPD-L1
IB: ADMA
IB: Vinculin
B16-tet-on-shPrmt1
e
MS023
GSK 3368715
μM
IB: mPD-L1
IB: ADMA
IB: Vinculin
MC38
f
IB: mPD-L1
IB: ADMA
IB: Vinculin
4T1
g
IB: mPD-L1
IB: ADMA
IB: Vinculin
B16
h
sgControl sgcGAS #1 sgcGAS #2
IB: mPD-L1
IB: mcGAS
IB: Vinculin
MC38
i
IB: mPD-L1
IB: mcGAS
IB: Vinculin
4T1
j
IB: mPD-L1
IB: cGAS
IB: Vinculin
B16
k
sgControl sgcGAS#1 sgcGAS#2
GSK3368715
IB: mPD-L1
IB: mcGAS
IB: ADMA
IB: Vinculin
CT26
l
MS023
IB: mPD-L1
IB: mcGAS
IB: ADMA
IB: Vinculin
MC38
m
GSK3368715
IB: mPD-L1
IB: mcGAS
IB: ADMA
IB: Vinculin
MC38
n
r=0.56,
p=1.77e-262
Color indicates different study
PD-L1 mRNA
cGAS mRNA
o
TNBC cells
R=0.448, p<0.0001
PD-L1 mRNA level (log 10)
cGAS mRNA level (log10)
q
HT-DNA (μg /mL)
IB: p-IRF3
IB: p-STING
IB: STING
IB: Vinculin
HCC1937
s
HT-DNA
Poly (I:C)
12h
DOX
IB: p-IRF3
IB: IRF3
IB: p-STING
IB: STING
IB: Vinculin
MBA-MD-231
-tet-on-shPRMT1
r
HT-DNA (μg /mL)
IB: p-IRF3
IB: p-STING
IB: STING
IB: Vinculin
SUM159PT
p
HT-DNA (μg/mL)
IB: p-IRF3
IB: IRF3
IB: Vinculin
MDA-MB-436

Supplementary Figure 6. Genetic or pharmaceutical ablation of PRMT1 elevates PD-L1 expression *in vitro* and *in vivo*.

a. Immunoblot analysis of mouse cell lines.

b-d. Genetic ablation of *Prmt1* elevates mPD-L1 expression in MC38 (b), 4T1 (c), and B16 cells (d). The tet-inducible stable cell lines were treated with indicated doses of DOX for 3 days to induce deletion of endogenous *Prmt1*, and harvested for immunoblot analysis.

e-g. Inhibition of PRMT1 elevates mPD-L1 expression in MC38, 4T1 and B16 cells. The MC38 (e), 4T1 (f), and B16 (g) mouse tumor cells were treated with indicated doses of MS023 or GSK3368715 for 48 hours, and harvested for immunoblot analysis.

h-j. Depletion of endogenous *cGAS* leads to reduce in mPD-L1 expression in MC38, 4T1 and B16 cells. The MC38 (h), 4T1 (i), and B16 (j) cells were infected with sgControl or sg*cGAS* lentivirus and selected with puromycin for 7 days, and then harvested for immunoblot analysis.

k. Inhibition of PRMT1 elevates mPD-L1 expression in a cGAS-dependent manner in CT26 cells. The stable cell lines as in Fig. 3C were treated with 6 μM of GSK3368715 for 48 hours and harvested for immunoblot analysis.

l, m. Inhibition of PRMT1 elevates mPD-L1 expression in a cGAS-dependent manner in MC38 cells. The stable cell lines as in fig. 12I were treated with 6 μM of MS023 (l) or GSK3368715 (m) for 48 hours and harvested for immunoblot analysis.

n. Correlation analysis of cGAS and PD-L1 mRNA expression in human BRCA samples in TCGA. Pearson correlation, r=0.56, p=1.77e-262. Different color indicates samples from different studies.

o. Correlation analysis of cGAS and PD-L1 mRNA expression in human BRCA cell lines. The mRNA levels were retrieved from *Marcotte R, et al., 2016, Cell*. Pearson correlation, r=0.448, p<0.0001. The blue color indicates triple negative breast cancer (TNBC) cell lines.

p-r. Immunoblot analysis of MDA-MB-436 (p), HCC1937 (q) and SUM159PT (r) cells after stimulation with indicated doses of HT-DNA for 12 hours.

s. Genetic ablation of *PRMT1* increases the cGAS/STING DNA sensing signaling in MBA-MD-231 cells. MDA-MB-231 cells were infected with tet-on-sh*PRMT1* lentivirus and selected with 1 μg/mL of puromycin for 7days. The stable cell lines were treated with DOX for 3 days, stimulated with HT-DNA or Poly(I:C) for 12 hours, followed by harvesting for immunoblot analysis. Source data are provided as a Source Data file.

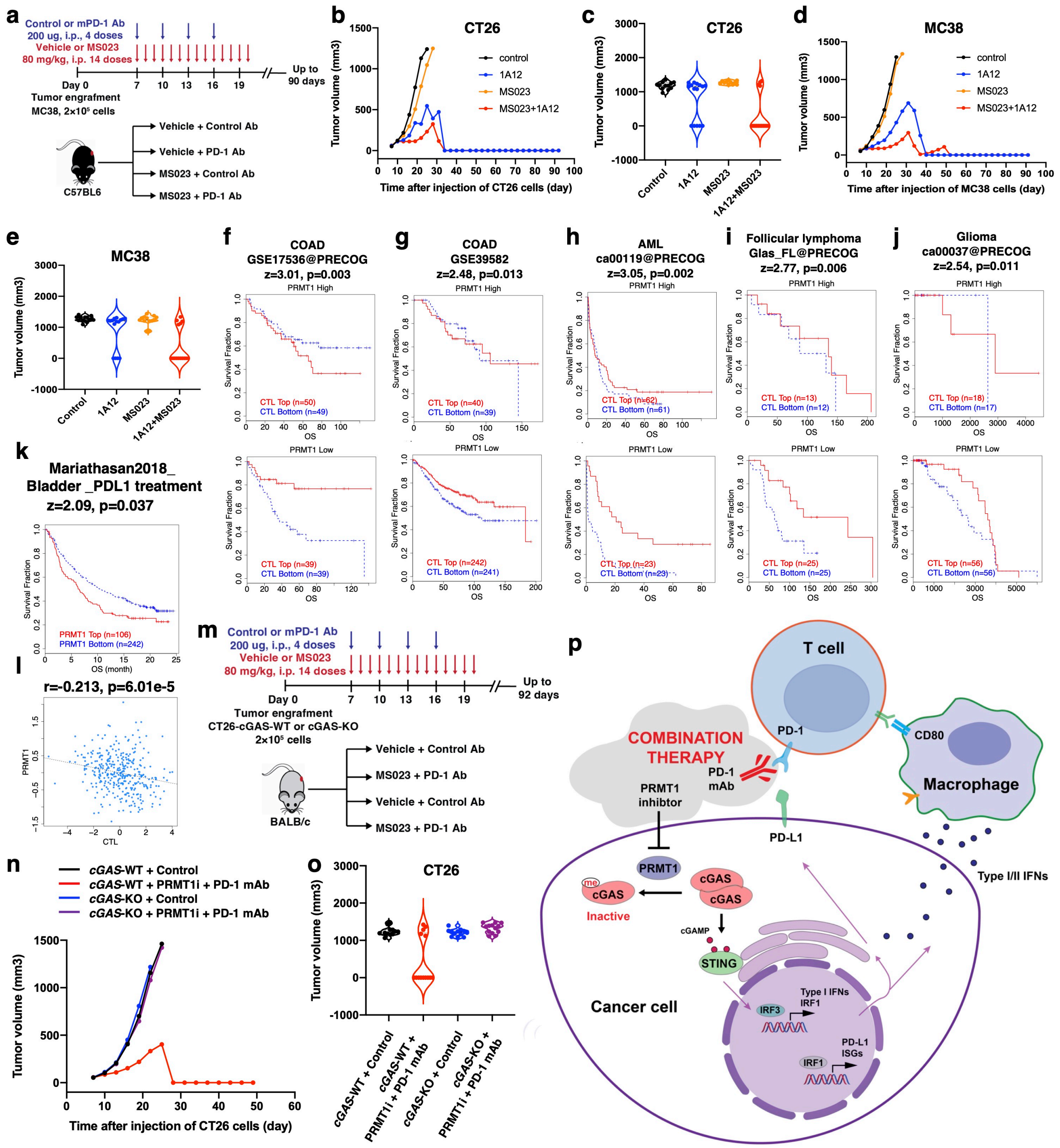

Supplementary Figure 7. PRMT1 inhibitor synergizes with the anti-PD-1 antibody in syngeneic tumor models.

a. A schematic diagram illustrates the animal experiment design for the MC38 syngeneic tumor model with PRMT1 inhibitor and anti-PD-1 antibody treatment.

b. The tumor volume curves of the CT26 tumor cell-derived syngeneic tumor after treated with either MS023, or anti-PD-1 antibody (1A12), or combination as shown in a.

c. The final tumor volume of the CT26 tumor cell-derived syngeneic tumor after treated with either MS023 or anti-PD-1 antibody (1A12), or combination as shown in a.

d. The tumor volume curves of the MC38 tumor cell-derived syngeneic tumor after treated with either MS023 or anti-PD-1 antibody (1A12), or combination as shown in b.

e. The final tumor volume of the MC38 tumor cell-derived syngeneic tumor after treated with either MS023 or anti-PD-1 antibody (1A12), or combination as shown in b.

f-j. Correlation analysis between PRMT1 expression with T cell dysfunction in Colon adenocarcinoma. (COAD), AML, lymphoma and glioma. The data were derived from Tide for COAD (GSE17536@PRECOG, f; GSE39582, g), AML (ca00119@PRECOG, h), lymphoma (Glas_FL@PRECOG, i) and glioma (ca00037@PRECOG, j). AML: acute myeloid leukemia; COAD: Colon adenocarcinoma. Kaplan-Meier survival curve was used to calculate the Cox-PH z-scores.

k. The survival response to PD-L1 treatment in bladder cancer (BLCA) patients with different PRMT1 expression, derived from Mariathasan2018_PDL1 by TIDE.

l. Correlation analysis between PRMT1 expression with CTL infiltration in bladder cancer.

m. A schematic diagram illustrates the animal experiment design for CT26-*cGAS-WT* and *cGAS-KO* tumor cells derived syngeneic tumor model, with PRMT1 inhibitor and anti-PD-1 antibody treatment.

n. The tumor volume curves of CT26-*cGAS*-WT and *cGAS*-KO tumor cells-derived syngeneic tumor after combinational treatment with MS023 and anti-PD-1 antibody as shown in m.

o. The final tumor volume of CT26-*cGAS*-WT and *cGAS*-KO tumor cells-derived syngeneic tumor after combinational treatment with MS023 and anti-PD-1 antibody as shown in m.

p. A schematic diagram shows that PRMT1-mediated arginine methylation of cGAS represses the cGAS/STING DNA sensing signaling, thus evading tumor immune surveillance. To this end, PRMT1 inhibition re-activates the cGAS/STING signaling, which promotes the expression type I interferon-regulated genes to facilitate antigen presentation. One the other hand, PRMT1 inhibition increased PD-L1 expression to represses cytotoxic T cell function, and further combination treatment with anti-PD-1 antibody releases PD-L-induced suppression on cytotoxic T cell, thus leading to tumor regression.

Source data are provided as a Source Data file.

Supplementary Table 1. Primers for RT-qPCR

Primers	Sequence
**mouse Actin-f**	5′-TACCACAGGCATTGTGATGG-3′
**mouse Actin-r**	5′-TCTCAGCTGTGGTGGTGAAG-3′
**mouse Ifnβ-f**	5′-GCTCCAAGAAAGGACGAACA-3′
**mouse Ifnβ-r**	5′-CATCTCTTGGATGGCAAAGG-3′
**mouse Cxcl10-f**	5’-CCAAGTGCTGCCGTCATTTTC-3’
**mouse Cxcl10-r**	5’-GGCTCGCAGGGATGATTTCAA-3’
**mouse Cxcl11-f**	5′-GGCTTCCTTATGTTCAAACAGGG-3′
**mouse Cxcl11-r**	5′-GCCGTTACTCGGGTAAATTACA-3′
**mouse Ccl5-f**	5’-CTCACCATATGGCTCGGACA-3’
**mouse Ccl5-r**	5’-CTTCTCTGGGTTGGCACACA-3’
**mouse Gbp2-f**	5′-CTGCACTATGTGACGGAGCTA-3′
**mouse Gbp2-r**	5′-GAGTCCACACAAAGGTTGGAAA-3′
**mouse Gbp4-f**	5′-GGAGAAGCTAACGAAGGAACAA-3′
**mouse Gbp4-r**	5′-TTCCACAAGGGAATCACCATTTT-3′
**mouse Ifit3-f**	5′-CCTACATAAAGCACCTAGATGGC-3′
**mouse Ifit3-f**	5′-ATGTGATAGTAGATCCAGGCGT-3′
**mouse Irf7-f**	5′-GAGACTGGCTATTGGGGGAG-3′
**mouse Irf7-r**	5′-GACCGAAATGCTTCCAGGG-3′
**mouse Rsad2-f**	5′-TGCTGGCTGAGAATAGCATTAGG-3′
**mouse Rsad2-f**	5′-GCTGAGTGCTGTTCCCATCT-3′
**mouse Sell-f**	5′-TACATTGCCCAAAAGCCCTTAT-3′
**mouse Sell-r**	5′-CATCGTTCCATTTCCCAGAGTC-3′
**mouse Tmem140-f**	5′-GCCCTGTGCCTGATGTTCTAC-3′
**mouse Tmem140-f**	5′-GCCCATGTCCTCCTTCCAC-3′
**mouse Uba7-f**	5′-CTACGAGCGACTCCATATACCT-3′
**mouse Uba7-r**	5′-TACACACAGGGTAGGGAGCAT-3′
**mouse Zbp1-f**	5′-AAGAGTCCCCTGCGATTATTTG-3′
**mouse Zbp1-r**	5′-TCTGGATGGCGTTTGAATTGG-3′

**hCXCL10-f**	5′-GTGGCATTCAAGGAGTACCTC-3′
**hCXCL10-r**	5′-TGATGGCCTTCGATTCTGGATT-3′
**hCCL5-f**	5′-CCAGCAGTCGTCTTTGTCAC-3′
**hCCL5-r**	5′-CTCTGGGTTGGCACACACTT-3′
**hGAPDH-f**	5′-ACAACTTTGGTATCGTGGAAGG-3′
**hGAPDH-r**	5′-GCCATCACGCCACAGTTTC-3′

Supplementary Table 2. sgRNA and shRNA

**shRNA sequence**	
**shPRMT1 #1 (TRCN0000290479)**	CCGGGTGTTCCAGTATCTCTGATTACTCGAGTAATCAGAG ATACTGGAACACTTTTTG
**shPRMT1 #2 (TRCN0000296582)**	CCGGCTTACCGCAACTCCATGTTTCCTCGAGGAAACATG GAGTTGCGGTAAGTTTTTG
**shPRMT1 #3 (TRCN0000310243)**	CCGGTGAGCGTTCCTAGGCGGTTTCCTCGAGGAAACCGC CTAGGAACGCTCATTTTTG
**shPRMT1 #4 (TRCN0000382079)**	GTACCGGGGACATGACATCCAAAGATTACTCGAGTAATCT TTGGATGTCATGTCCTTTTTTG
**tet-on-shPRMT1-UTR**	CCGGTGAGCGTTCCTAGGCGGTTTCCTCGAGGAAACCGC CTAGGAACGCTCATTTTTG
**sgRNA sequence**	
**sghcGAS**	AAGTGCGACTCCGCGTTCAGAGG
**sghSTING**	GGTGCCTGATAACCTGAGTATGG
**sgmcGAS#1**	AAATTCAAAAGAATTCCACGAGG
**sgmcGAS#2**	TGATAAGAAGTGTTACAGCAGGG

Fig. S1a

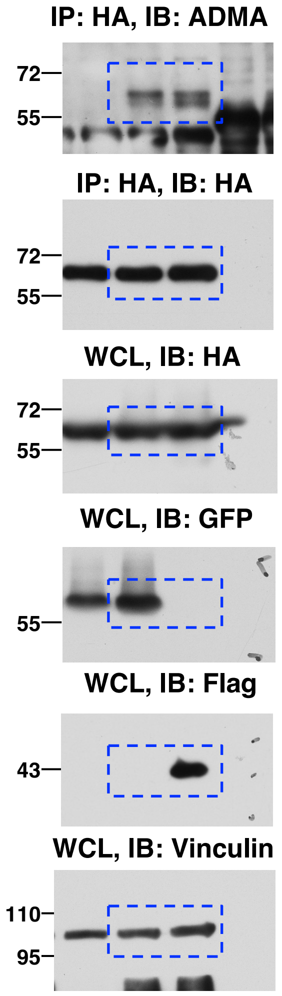

Fig. S1b

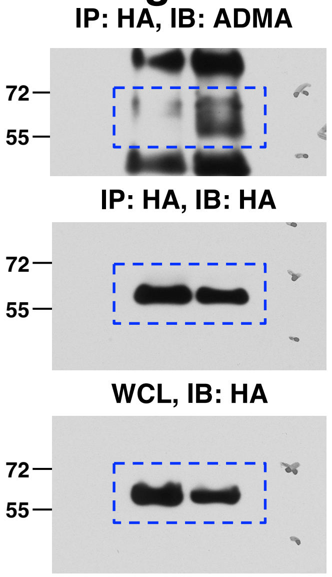

Fig. S1d

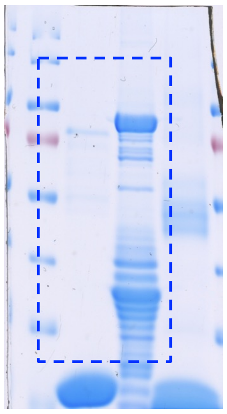

Fig. S1f

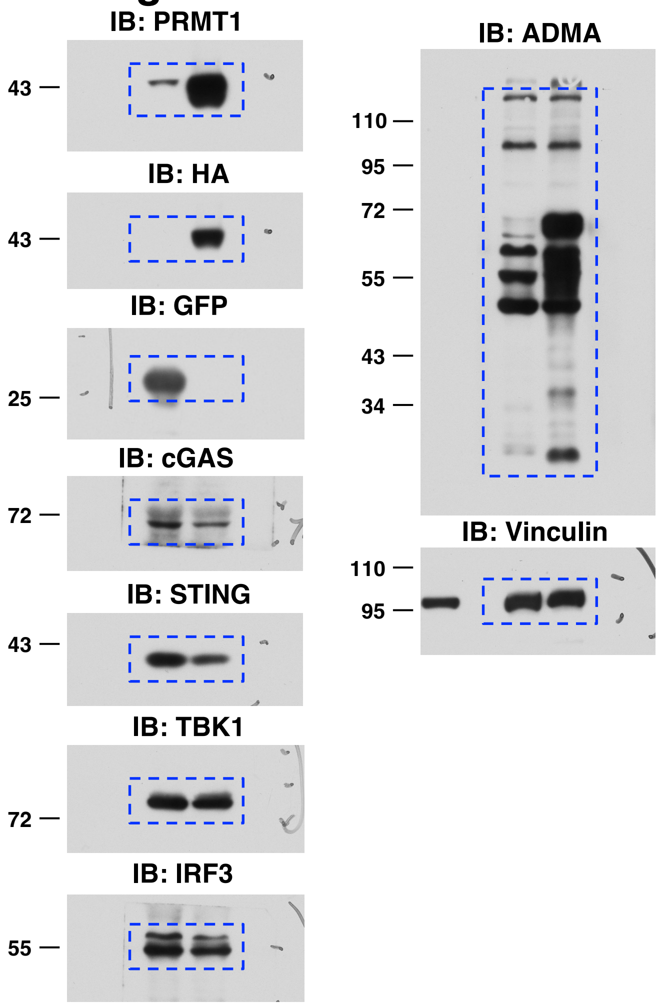

Fig. S1i

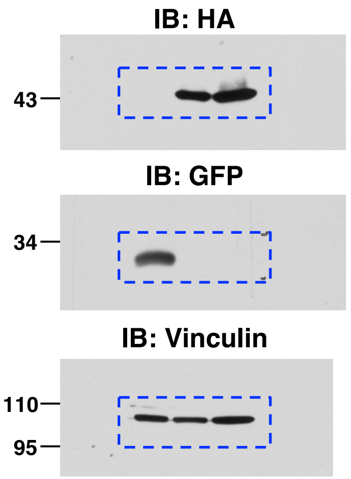

Fig. S1c

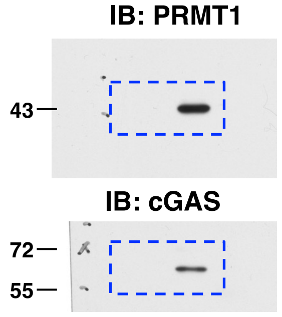

Fig. S1g

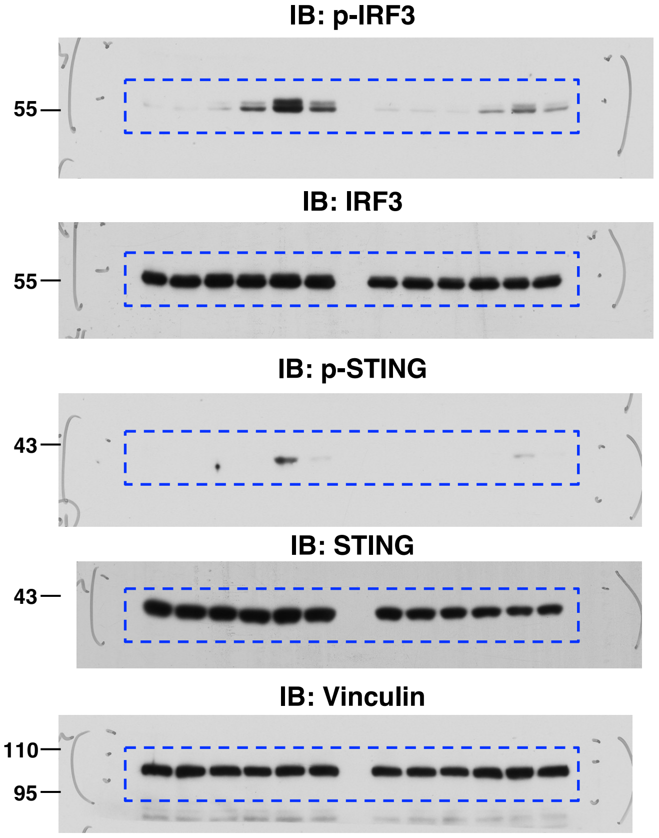

Fig. S1j

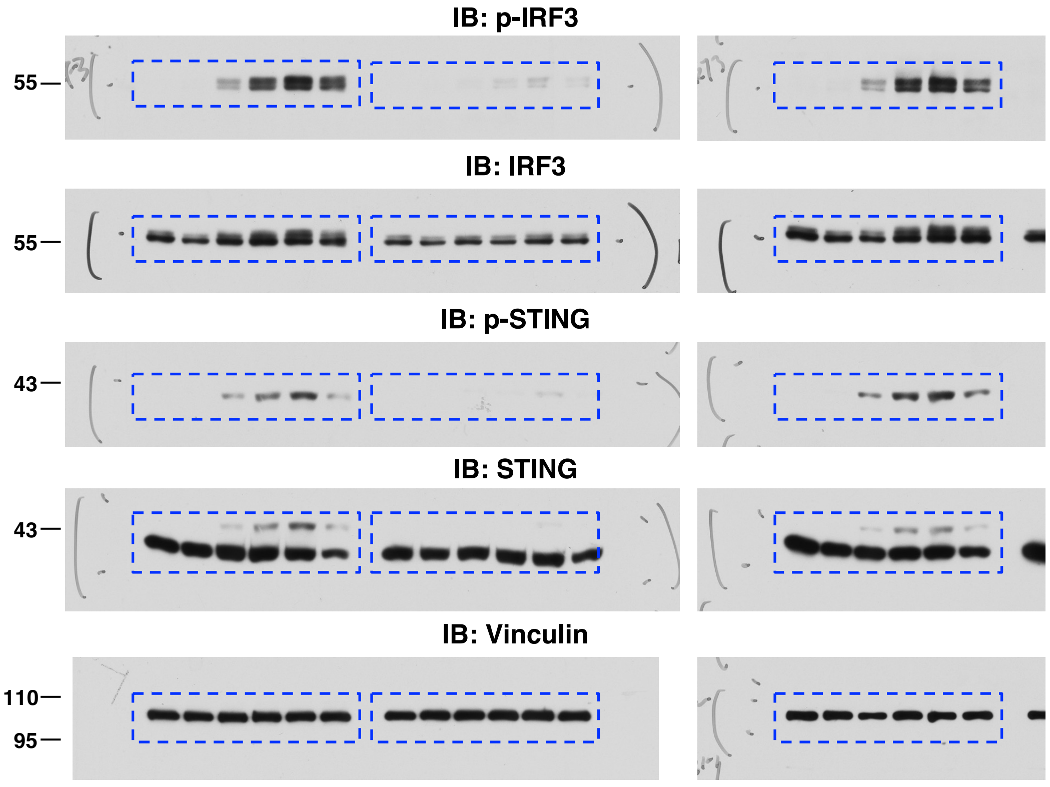

Fig. S2a

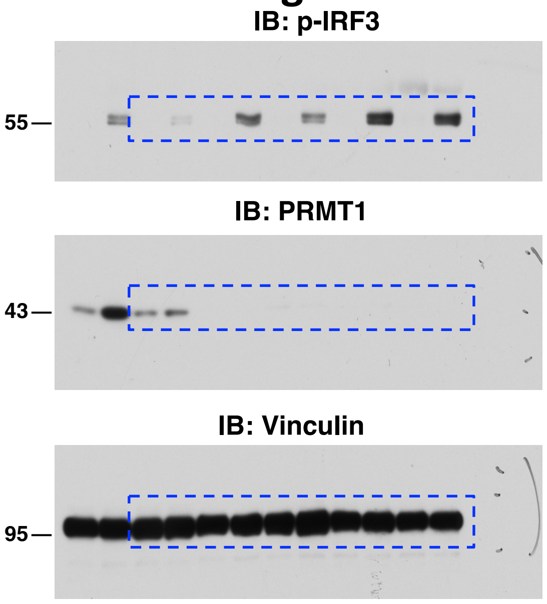

Fig. S2b

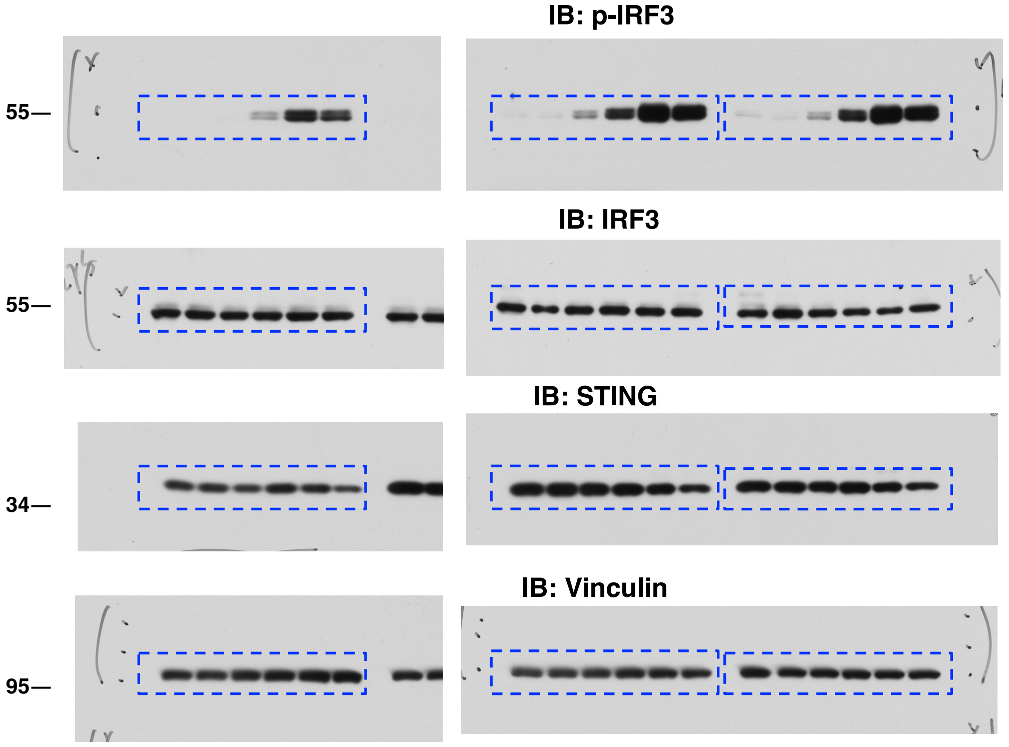

Fig. S2d

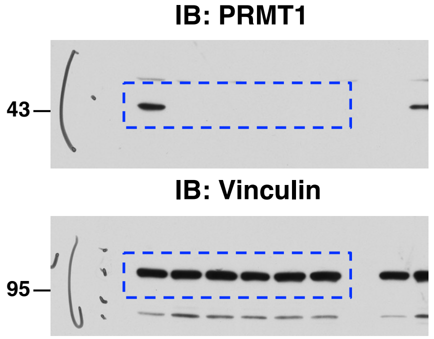

Fig. S2e

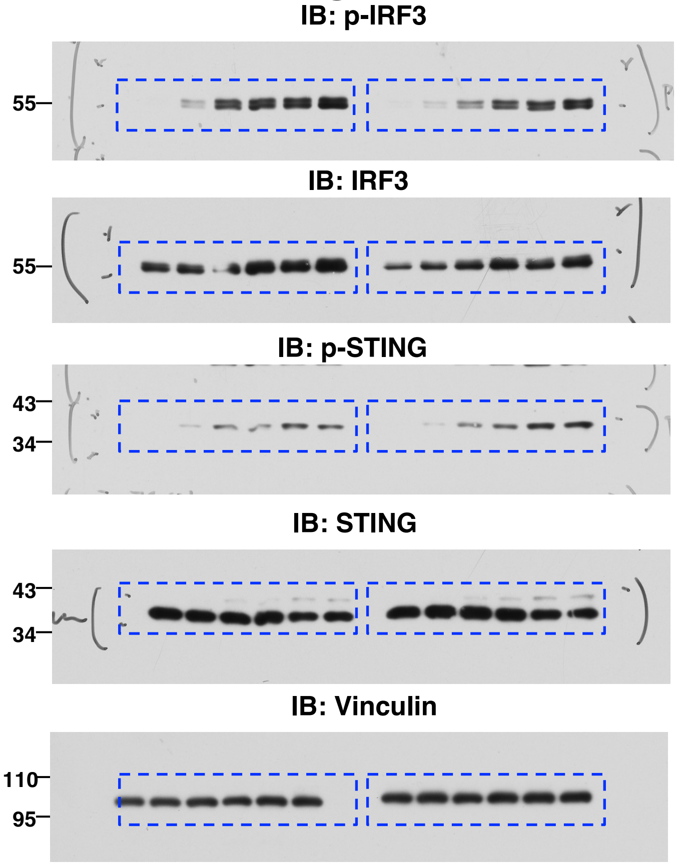

Fig. S2g

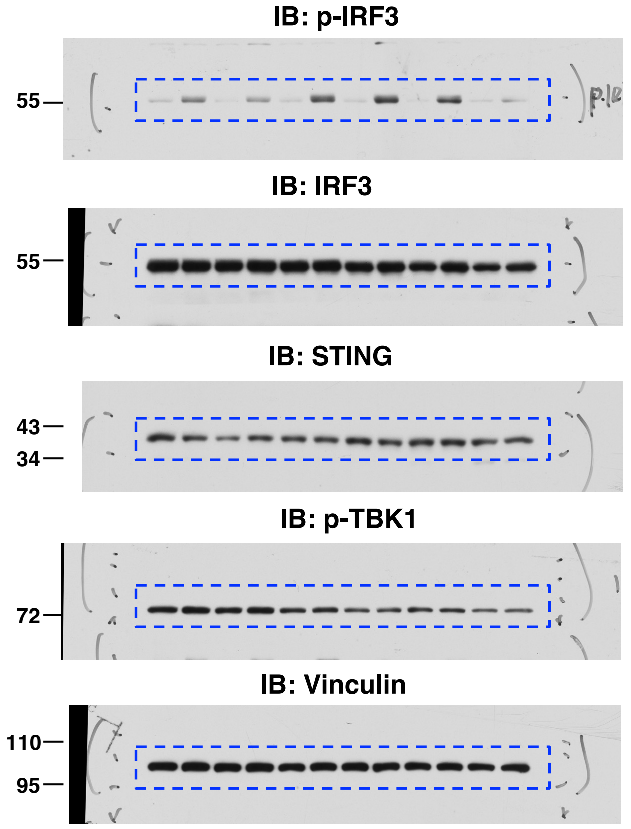

Fig. S2h

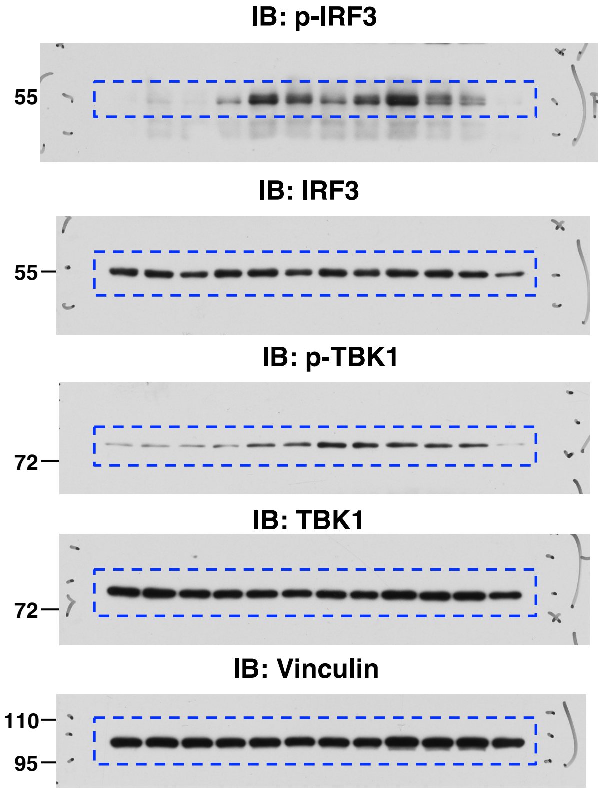

Fig. S2i

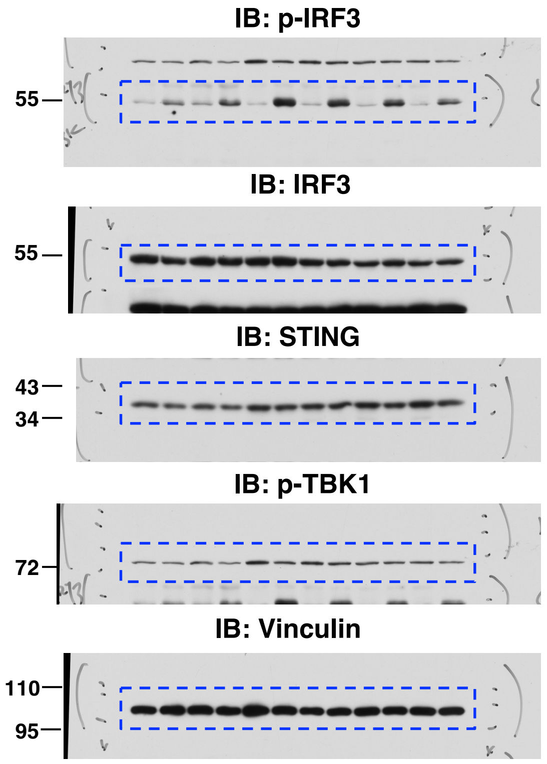

Fig. S2j

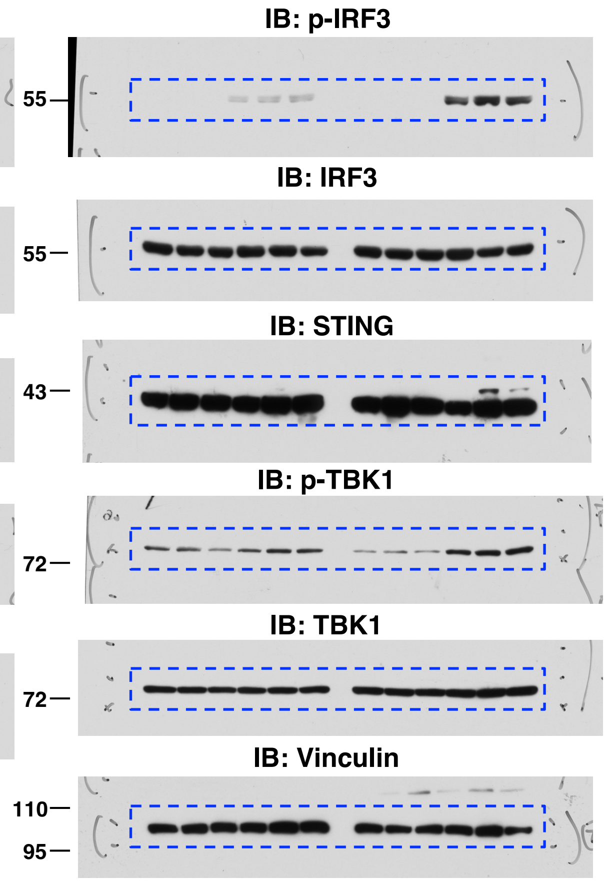

Fig. S2k

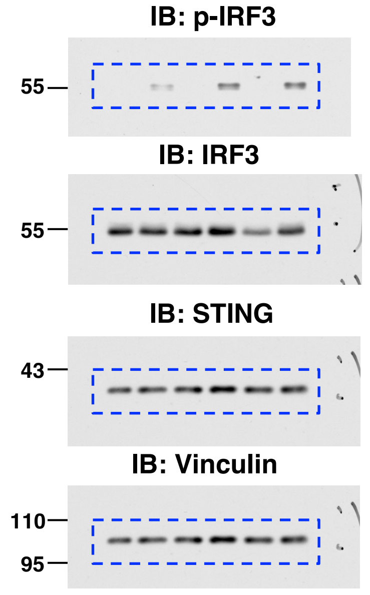

Fig. S2m

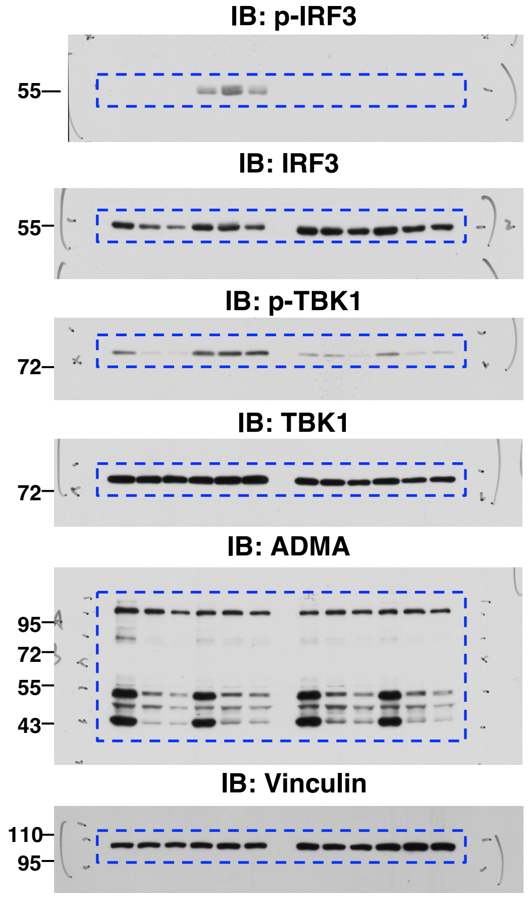

Supplement: Supplementary file 1 — Supplementary Information [file 41467_2023_38443_MOESM1_ESM.pdf]
