# Supplementary material for: PRMT1 mediated methylation of cGAS suppresses anti-tumor immunity
Source: Nat Commun. 2023 May 17;14:2806. doi: 10.1038/s41467-023-38443-3 (PMC10188589; doi:10.1038/s41467-023-38443-3)
Supplement: Supplementary file 2 — Reporting Summary [file 41467_2023_38443_MOESM2_ESM.pdf]

nature portfolio

Corresponding author(s): Wenyi Wei

Last updated by author(s): 04/08/2023

Reporting Summary

Nature Portfolio wishes to improve the reproducibility of the work that we publish. This form provides structure for consistency and transparency in reporting. For further information on Nature Portfolio policies, see our Editorial Policies and the Editorial Policy Checklist.

Statistics

For all statistical analyses, confirm that the following items are present in the figure legend, table legend, main text, or Methods section.

n/a	Confirmed	
☐	☒	The exact sample size (*n*) for each experimental group/condition, given as a discrete number and unit of measurement
☐	☒	A statement on whether measurements were taken from distinct samples or whether the same sample was measured repeatedly
☐	☒	The statistical test(s) used AND whether they are one- or two-sided<br>*Only common tests should be described solely by name; describe more complex techniques in the Methods section.*
☒	☐	A description of all covariates tested
☒	☐	A description of any assumptions or corrections, such as tests of normality and adjustment for multiple comparisons
☐	☒	A full description of the statistical parameters including central tendency (e.g. means) or other basic estimates (e.g. regression coefficient) AND variation (e.g. standard deviation) or associated estimates of uncertainty (e.g. confidence intervals)
☐	☒	For null hypothesis testing, the test statistic (e.g. *F*, *t*, *r*) with confidence intervals, effect sizes, degrees of freedom and *P* value noted<br>*Give P values as exact values whenever suitable.*
☒	☐	For Bayesian analysis, information on the choice of priors and Markov chain Monte Carlo settings
☒	☐	For hierarchical and complex designs, identification of the appropriate level for tests and full reporting of outcomes
☐	☒	Estimates of effect sizes (e.g. Cohen's *d*, Pearson's *r*), indicating how they were calculated

Our web collection on statistics for biologists contains articles on many of the points above.

Software and code

Policy information about availability of computer code

Data collection	No software was used for data collection.
Data analysis	Graphic were generated using GraphPad PRISM8. For RNA-seq data analysis, data were aligned to mm10 by Salmon 1.4.0 using the default parameters. The DEGs were calculated using DESeq2. For GSEA analysis, we used the GSEA tool v.4.2.2, with the MSigDB v.7.1 Hallmarks gene sets collection and the 'classic' method for calculating enrichment scores.

For manuscripts utilizing custom algorithms or software that are central to the research but not yet described in published literature, software must be made available to editors and reviewers. We strongly encourage code deposition in a community repository (e.g. GitHub). See the Nature Portfolio guidelines for submitting code & software for further information.

Data

Policy information about availability of data

All manuscripts must include a data availability statement. This statement should provide the following information, where applicable:

- Accession codes, unique identifiers, or web links for publicly available datasets
- A description of any restrictions on data availability
- For clinical datasets or third party data, please ensure that the statement adheres to our policy

Data supporting the findings of this work are available within the paper and the Supplementary Information. RNA-seq data used to support the present study have been deposited in the Gene Expression Omnibus with an access number of GSE203466. Further information and requests for resources and reagents should be directed to the lead author, W.W. Source data are provided with the paper.

March 2021

The customized genomic analysis was based on The Cancer Genome Atlas (TCGA) data (https://cancergenome.nih.gov/). The data for PRMT1 expression and survival of patients with bladder cancer after PD-L1 antibody treatment were generated using the TIDE tool (http://tide.dfci.harvard.edu) and the source data are based on the Mariathasan2018_PDL1cohort. The association between PRMT1 expression level and T cell dysfunction in multiple cancer types were generated using the TIDE tool and the source data are derived from data in TCGA and PRECOG. The expression data of cGAS and PD-L1 in a panel of BRCA cell lines were obtained from GEO dataset GSE73526. RNA-seq data used to support the present study have been deposited in the Gene Expression Omnibus (GSE203466). The expression data of cGAS and PD-L1 were obtained from GEO dataset GSE73526.

Human research participants

Policy information about studies involving human research participants and Sex and Gender in Research.

Reporting on sex and gender	N/A
Population characteristics	N/A
Recruitment	N/A
Ethics oversight	N/A

Note that full information on the approval of the study protocol must also be provided in the manuscript.

Field-specific reporting

Please select the one below that is the best fit for your research. If you are not sure, read the appropriate sections before making your selection.

- [x] Life sciences
- [ ] Behavioural & social sciences
- [ ] Ecological, evolutionary & environmental sciences

For a reference copy of the document with all sections, see nature.com/documents/nr-reporting-summary-flat.pdf

Life sciences study design

All studies must disclose on these points even when the disclosure is negative.

Sample size	For animal experiments, 15 mice have been used in each group according to previous study in the lab (PMID: 31270456, PMID: 32839551, PMID: 33909988).
Data exclusions	For the syngeneic mouse model experiments, those mice without tumor at day 7 after implantation were excluded, and then all mice were randomly assigned into different treatment groups. For figure 7a, a total of 8 mice were excluded due to failure in tumor implantation. For Figure 7c, atotal of 9 mice were excluded.
Replication	All biochemical experiments were repeated at least 3 times and all animal works in Figure 5 and 7 were repeated twice unless otherwise indicated, and all attempts at replication were successful.
Randomization	Animal were randomly grouped before treatment. Randomization is not relevant to other experiments.
Blinding	No blinding is applicable to the study, as all cell and animal treatment conditions and groups are clear to the researchers.

Reporting for specific materials, systems and methods

We require information from authors about some types of materials, experimental systems and methods used in many studies. Here, indicate whether each material, system or method listed is relevant to your study. If you are not sure if a list item applies to your research, read the appropriate section before selecting a response.

Materials & experimental systems

n/a	Involved in the study	
[ ]	[x]	Antibodies
[ ]	[x]	Eukaryotic cell lines
[x]	[ ]	Palaeontology and archaeology
[ ]	[x]	Animals and other organisms
[x]	[ ]	Clinical data
[x]	[ ]	Dual use research of concern

Methods

n/a	Involved in the study	
[x]	[ ]	ChIP-seq
[ ]	[x]	Flow cytometry
[x]	[ ]	MRI-based neuroimaging

Antibodies

Antibodies used	The anti-ADMA (13522), anti-human cGAS (15102), anti-mouse cGAS (31659), anti-STING (13647), anti-p-STING (Ser366, 50907), anti-IRF3 (11904), anti-mouse p-IRF3 (Ser 396, 29047), anti-TBK1 (3504) , anti-p-TBK1 (Ser172, 5483), anti-PRMT1 (2449), anti-human PD-L1 (13684), anti-GST (2625) antibodies were obtained from Cell Signaling Technology. Anti-human p-IRF3 (Ser 396, ab76493) and anti-mouse PD-L1 (EPR20529, ab213480) antibodies were obtained from Abcam. Anti-Tubulin (sc-8035), anti-GFP (B-2, sc-9966) and anti-PRMT1 (B-2, sc-166963) antibodies were obtained from Santa Cruz Biotechnology. Mouse monoclonal anti-HA.11 epitope tag (16B12, 901513) was obtained from BioLegend. Anti-Vinculin (V9131), rabbit polyclonal anti-HA (H6908), Mouse monoclonal anti-Flag (Clone M2, F3165), Rabbit polyclonal anti-Flag (F7425), anti-mouse IgG (whole molecule)-peroxidase (A4416) and anti-rabbit IgG (whole molecule)-peroxidase (A4914) were obtained from Sigma-Aldrich. Mouse monoclonal ANTI-FLAG M2 affinity agarose gel (A2220) and Mouse monoclonal anti-HA-agarose (A2095) were obtained from Sigma-Aldrich. The antibodies used for FACS were as follow: anti-CD45 (30-F11, #103140, BioLegend); anti-CD3 BV785 (17A2, #100232, BioLegend); anti-CD3 BV785 (145-2C11, #100355, BioLegend); anti-CD4 BV650 (GK1.5, #100469; RM4-5, #100555, BioLegend); anti-CD8a BV711 (53-6.7 #100748, BioLegend); anti-PD-L1 (10F.9G2, # 124308, BioLegend); anti-CD11b BV650 (M1/70, # 101259, BioLegend); anti-CD11c BV510 (N418, # 117353, BioLegend); anti-CD80 Pacific Blue (16-10A1, 104724, BioLegend), anti-FoxP3 PerCP-Cy™5.5 (Clone R16-715, BD563902), anti-GranB Pacific Blue (GB11, #515408, BioLegend).
Validation	All antibodies used in this study were obtained from reputable commercial vendors.<br><br>Biolegend: "Antibody clones are then tested in a variety of assays to see which applications they are suited for....Thus, the clone cross-validates itself by demonstrating functionality across orthogonal testing methods."<br><br>Cell Signaling Technology: "This product has met all of the quality control standards defined by Cell Signaling Technology, Inc."<br><br>Abcam: "Our Abpromise guarantee covers the use of it in the following tested applications."<br><br>Sigma :"SSigma-Aldrich warrants, that at the time of the quality release or subsequent retest date this product conformed to the information contained in this publication. "

Eukaryotic cell lines

Policy information about cell lines and Sex and Gender in Research

Cell line source(s)	MDA-MB-231 (HTB-26, ATCC) was from the A. Toker laboratory (BIDMC). Mouse embryonic fibroblasts (MEFs), SK-BR-3, BT-474, T47D, MB157, MDA-MB-468, HCC38, HCC1143, HCC1806, SUM149PT, Cal-51, Cal120, and BT-20 cells were from the P. Sicinski laboratory (Dana-Farber Cancer Institute). Mouse tumor-derived CT26, 4T1, and B16F10 cells were routinely cultured in Gordon Freeman's laboratory (Dana-Farber Cancer Institute). Mouse tumor-derived MC38 cell line was a kind gift from Dr. Arlene Sharpe (Harvard Medical School). The iBMDM cells was a gift from Dr. Jonathan C. Kagan (Harvard Medical School). BT-549, Hs587T, HEK293T, HeLa, MDA-MB-436, HCC1937, SUM159PT, ZR-75-1, ZR-75-30, CAMA-1, HCC1428, AU565, HCC1954 and HCC-1569 cells were purchased from American Type Culture Collection (ATCC).
Authentication	All cell lines were authenticated.
Mycoplasma contamination	All cells were tested and validated for mycoplasma-free.
Commonly misidentified lines (See ICLAC register)	No commonly misidentified cell lines were used in the study.

Animals and other research organisms

Policy information about studies involving animals; ARRIVE guidelines recommended for reporting animal research, and Sex and Gender in Research

Laboratory animals	6-week old BABL/C and C57/B6 mice were used.<br>Dark/light cycle: 12 hours of light/12 hours of dark.<br>Ambient temperature range: 68-79F (20-26C)<br>Ambient humidity range: 30-70%
Wild animals	This study did not involved wild animals.
Reporting on sex	Female
Field-collected samples	This study did not involved samples collected from the field.
Ethics oversight	Animal experiments were approved by Dana-Farber Cancer Institute Institutional Animal Care and Use Committee (IACUC; protocol number 04–047) or Beth Israel Deaconess Medical Center (BIDMC) Institutional Animal Care and Use Committee (IACUC: Protocol #043–2019), and performed in accordance with guidelines established by NIH Guide for the care and use of laboratory animals.

Note that full information on the approval of the study protocol must also be provided in the manuscript.

Flow Cytometry

Plots

Confirm that:

- [ ] The axis labels state the marker and fluorochrome used (e.g. CD4-FITC).
- [x] The axis scales are clearly visible. Include numbers along axes only for bottom left plot of group (a 'group' is an analysis of identical markers).
- [ ] All plots are contour plots with outliers or pseudocolor plots.
- [x] A numerical value for number of cells or percentage (with statistics) is provided.

Methodology

Sample preparation	For FACS staining, 100 µL cells in MACS buffer were blocked by 1 µg Fc blocker ((anti-mouse CD16/CD32 antibody, Clone 2.4G2, Bio X Cell) for 15 minutes, then stained with Live-Dead NIR (Invitrogen, #L10119) and individual antibodies at RT for 40 minutes, washed with MACS buffer. Then, the stained cells were further fixed and permeabilizated using the eBioscience Foxp3/Transcription Factor Staining Buffer (ThermoFisher, #00-5533-00), followed by staining with Foxp3 and GramB antibodies at RT for 40 minutes, and washed with MACS buffer.
Instrument	BD LSR Fortessa X-20
Software	FlowJo_V10.6.1 software (Tree Star)
Cell population abundance	10,000 cells were gated for CD45+ cells.
Gating strategy	T cells gating strategy: gate cells exclude dead cells and debris based on cells size, then gate live cells based on Live-Dead NIR negative cells, then gate CD45+ cells, then gate CD45+CD3+ cells, then gate CD45+CD3+CD8+ cells and CD45+CD3+CD4+ cells. Macrophage gating strategy: gate cells exclude dead cells and debris based on cell size, then gate live cells based on Live-Dead NIR negative cells, then gate CD45+ cells, then gate CD45+CD11b+ cells.

- [x] Tick this box to confirm that a figure exemplifying the gating strategy is provided in the Supplementary Information.
