# Supplementary material for: PRMT1 mediated methylation of cGAS suppresses anti-tumor immunity
Source: Nat Commun. 2023 May 17;14:2806. doi: 10.1038/s41467-023-38443-3 (PMC10188589; doi:10.1038/s41467-023-38443-3)

Figure 1a

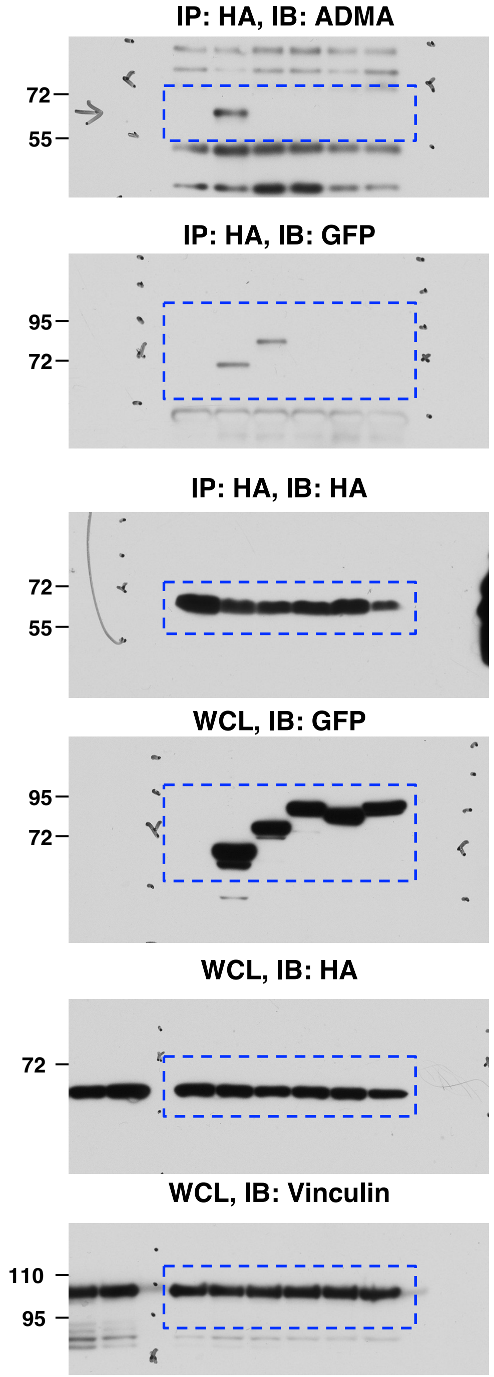

Figure 1b

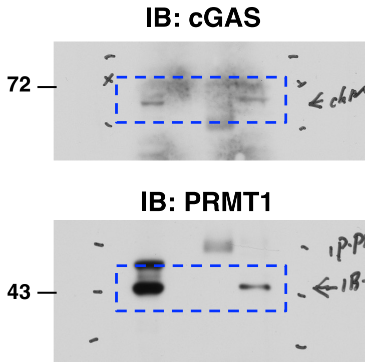

Figure 1c

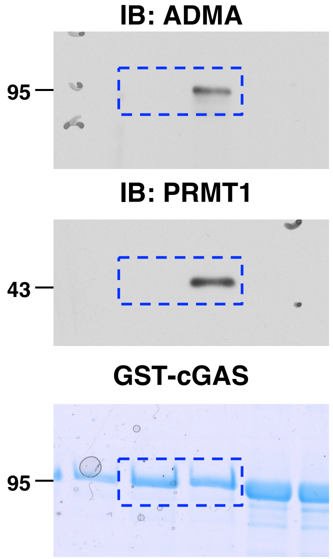

Figure 1d

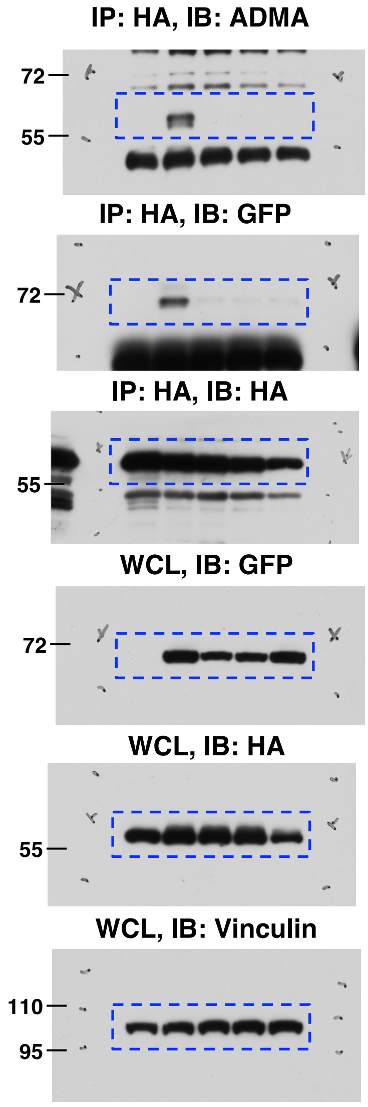

Figure 1e

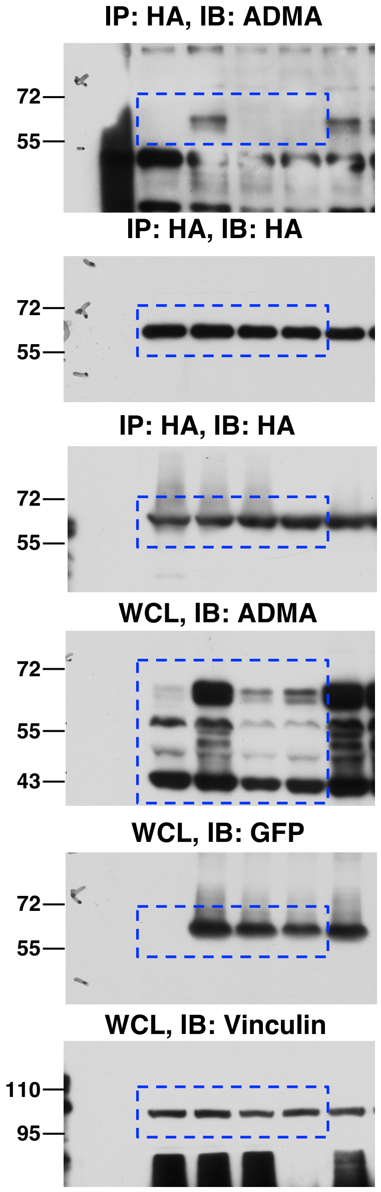

Figure 1h

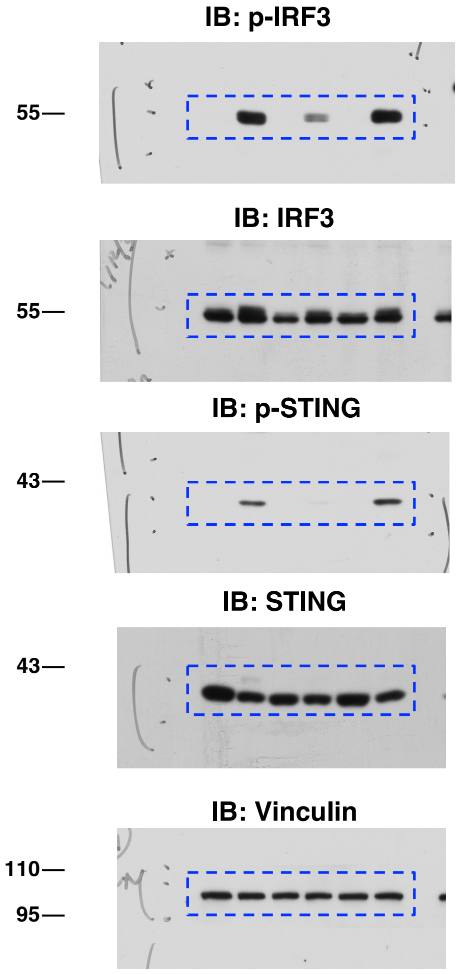

Figure 1g

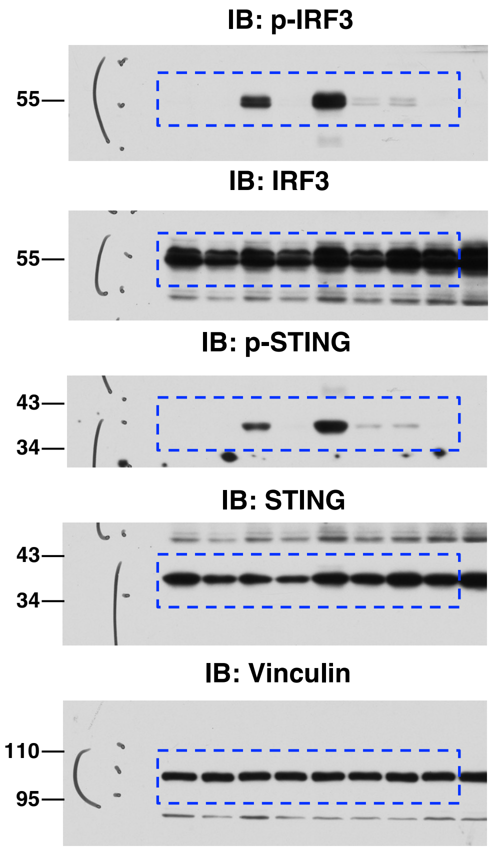

Figure 2a

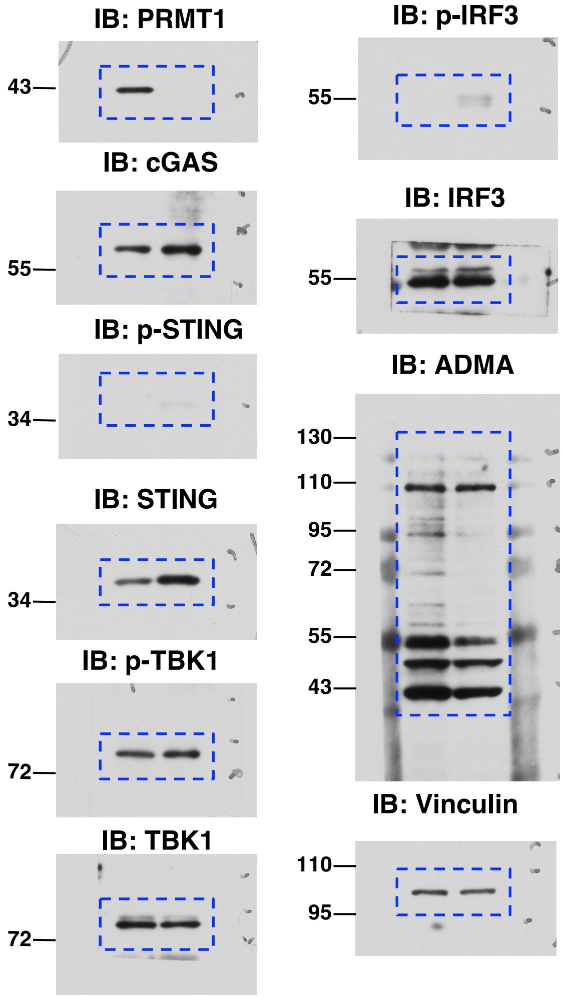

Figure 2b

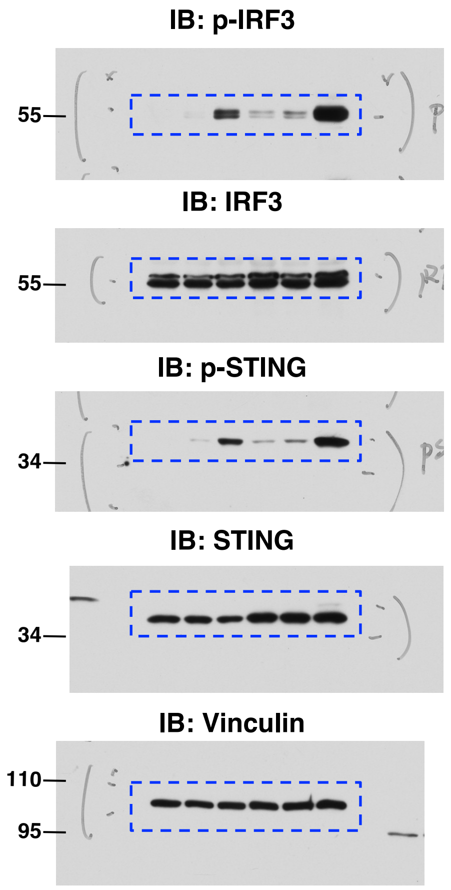

Figure 2e

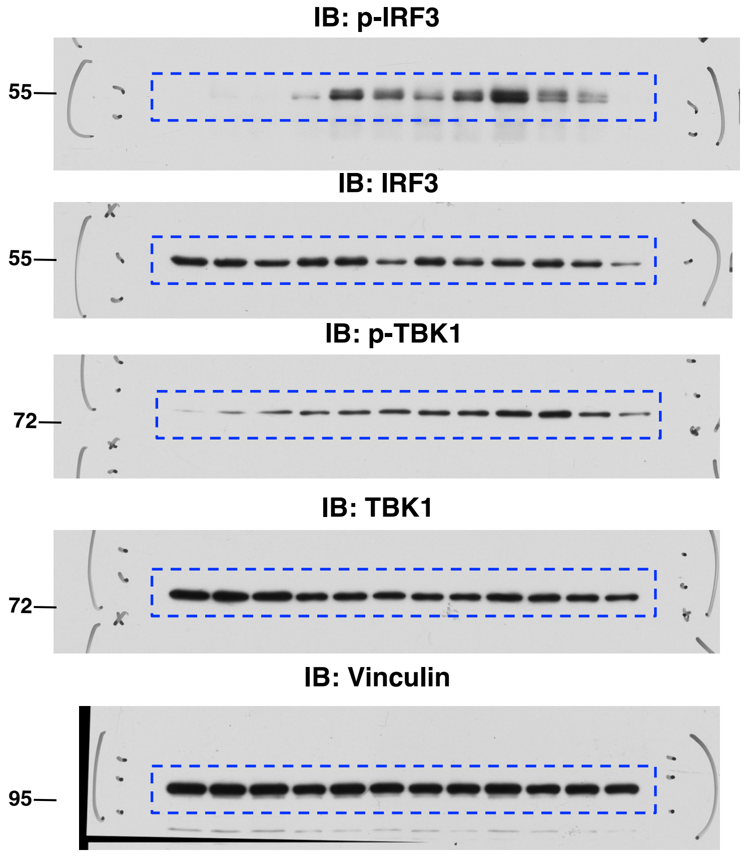

Figure 2f

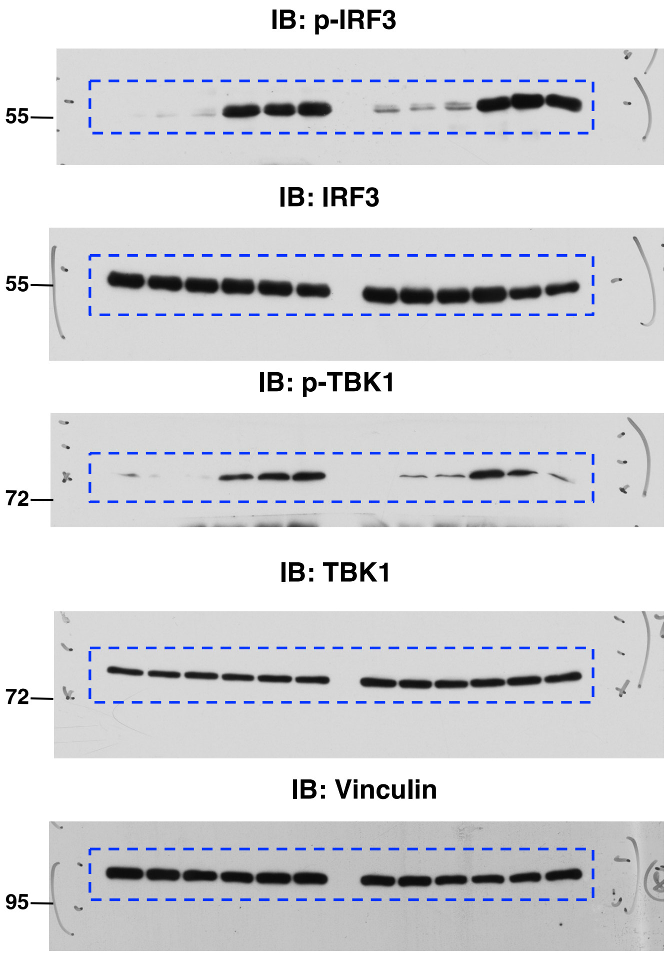

Figure 2g

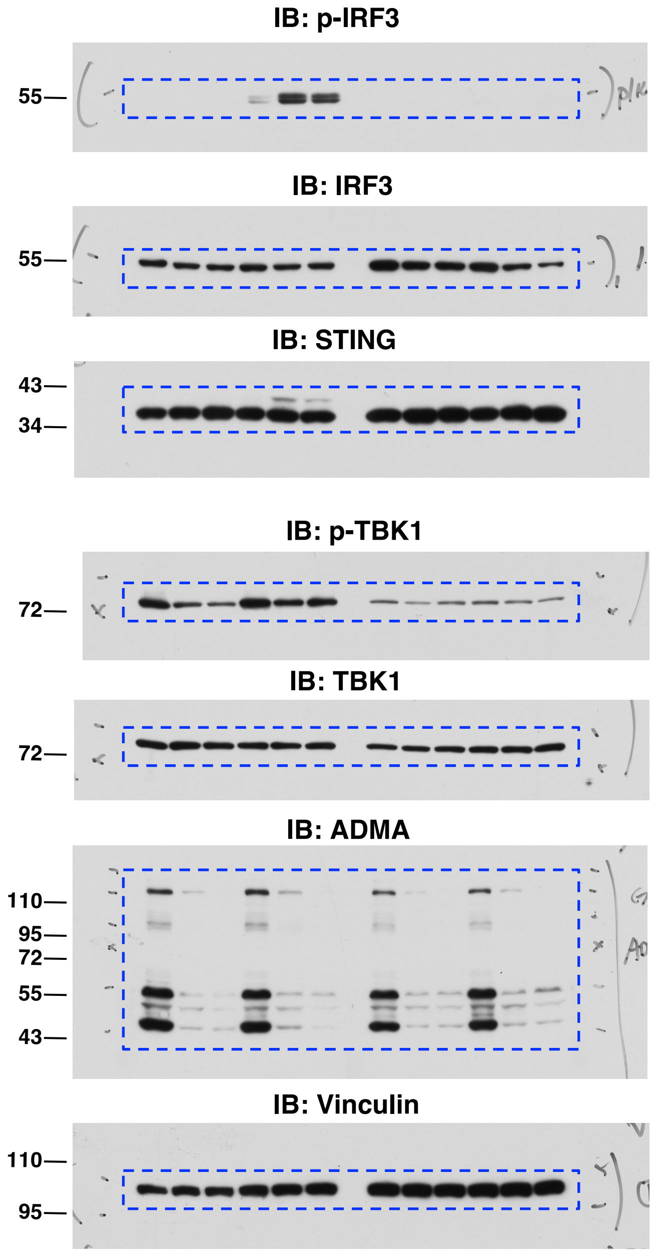

Figure 3b

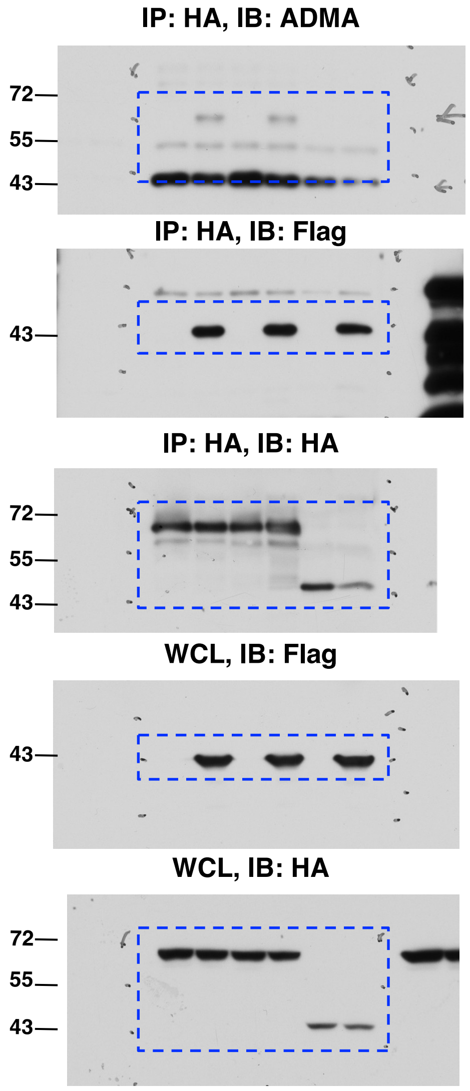

Figure 3c

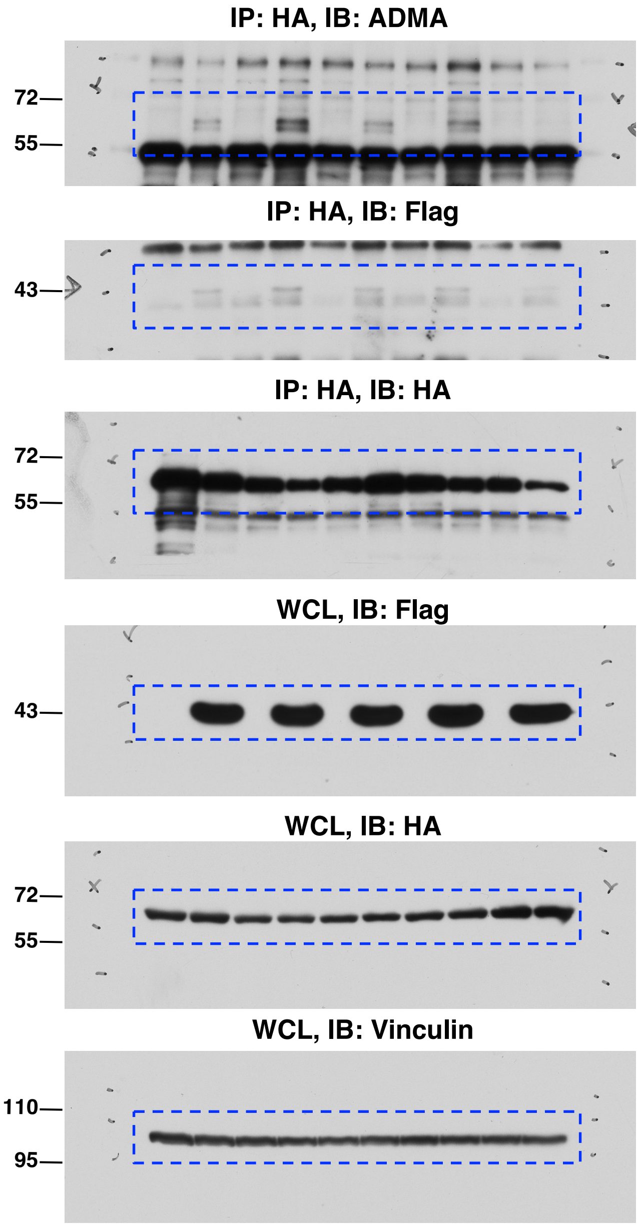

Figure 3d

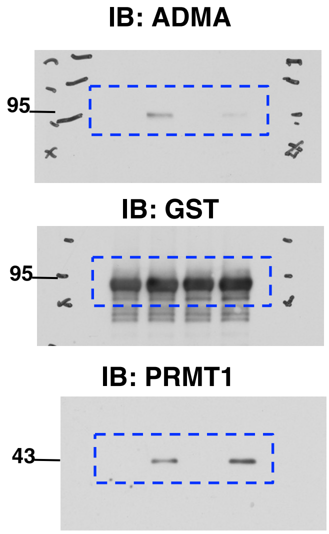

Figure 3e

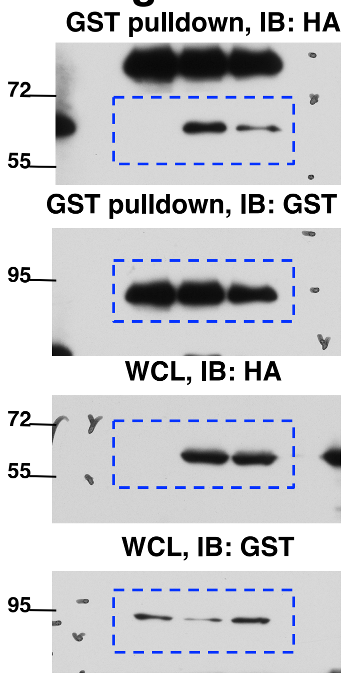

Figure 3i

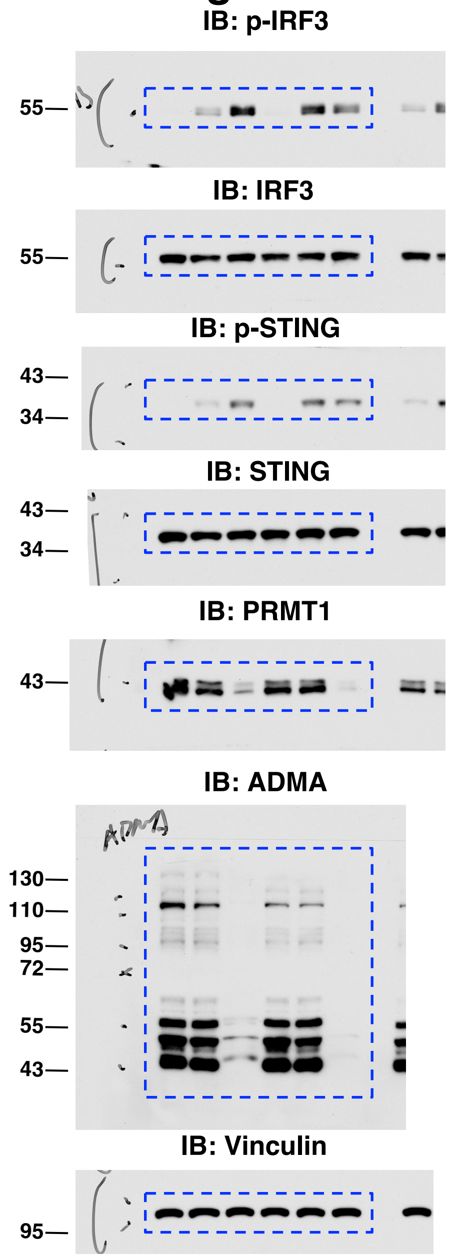

Figure 3f

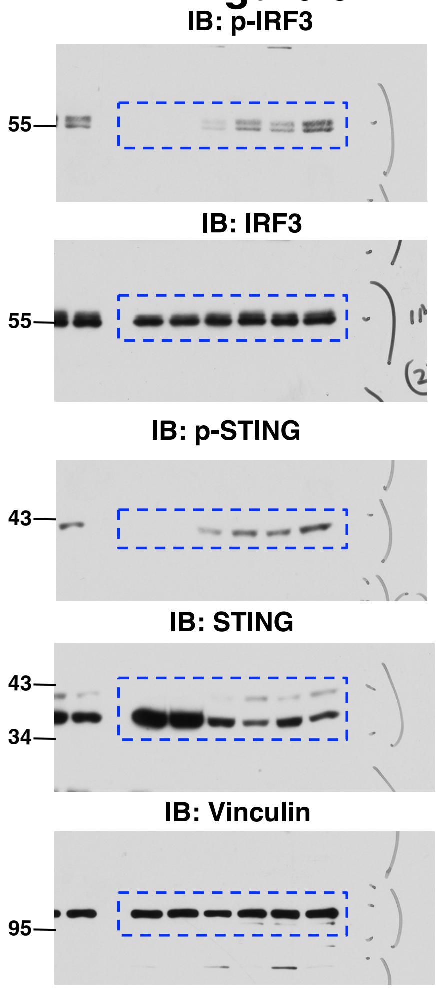

Figure 3g

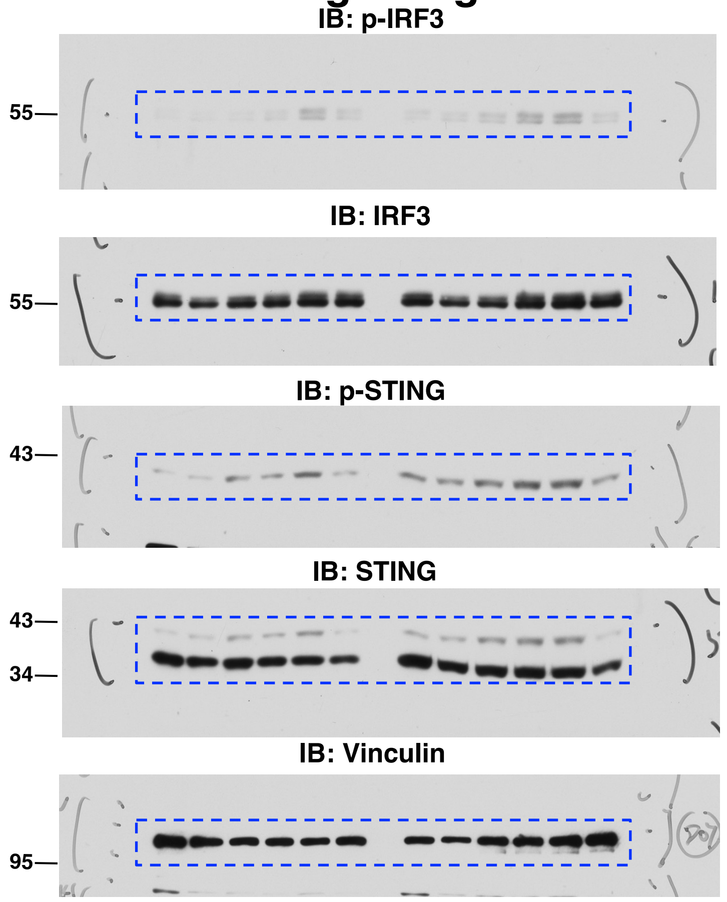

Figure 3j

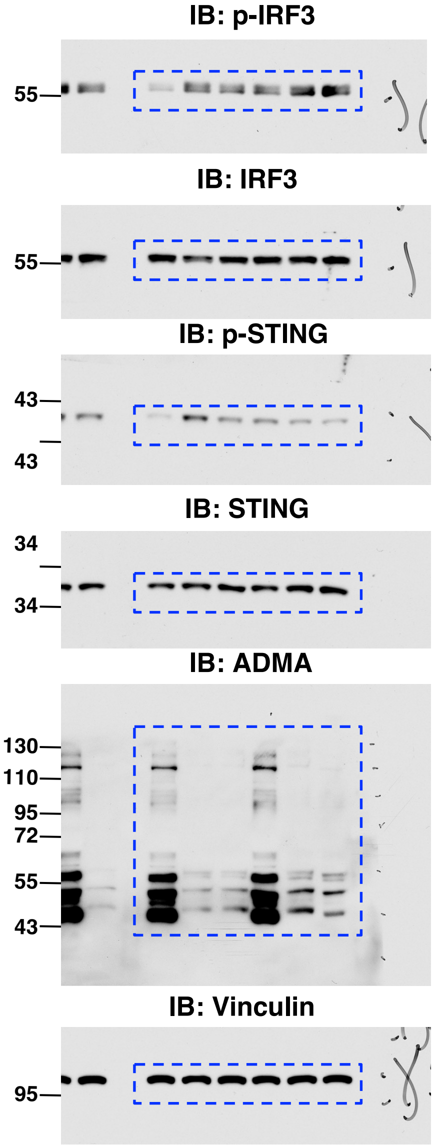

Figure 4b

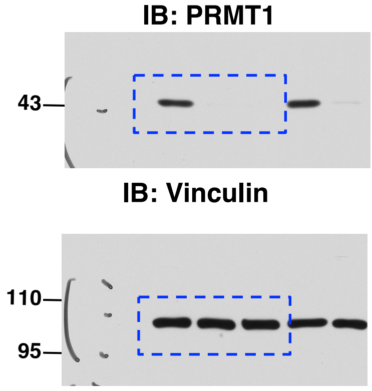

Figure 5a

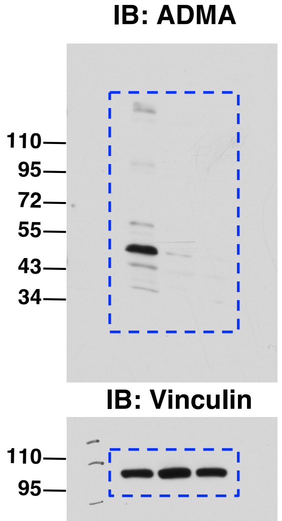

Figure 6a

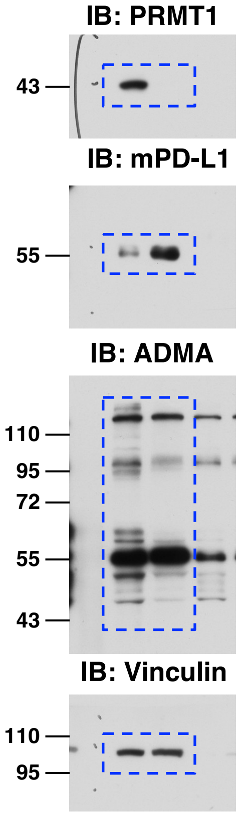

Figure 6b

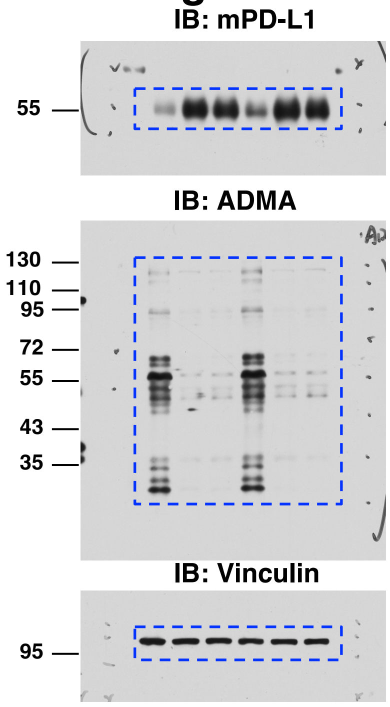

Figure 6c

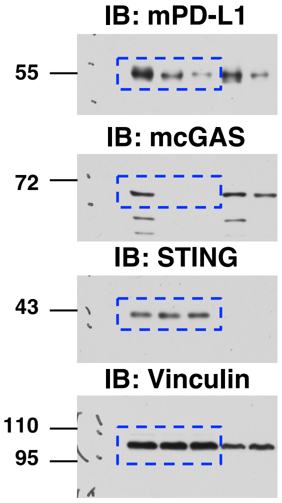

Figure 6d

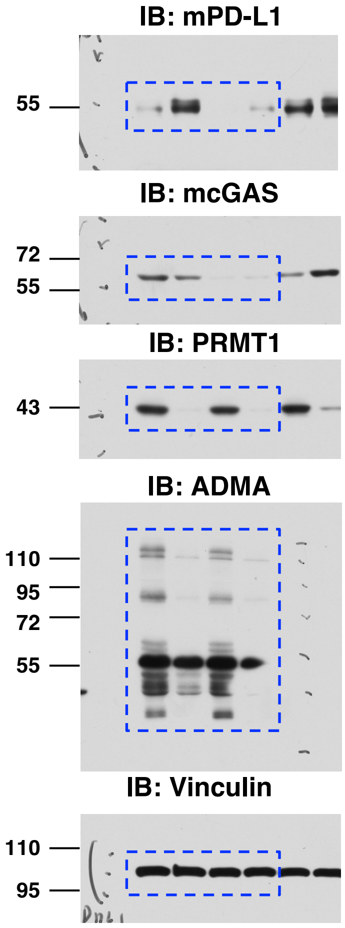

Figure 6e

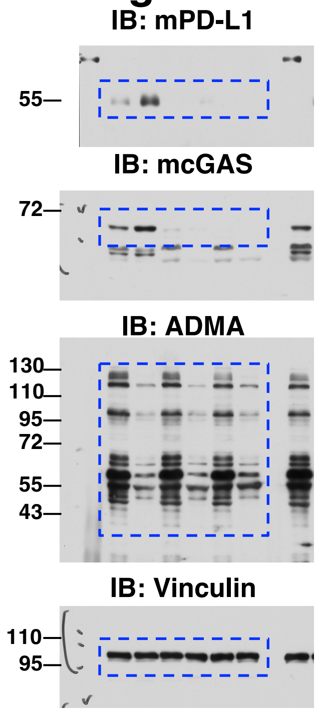

Figure 6f

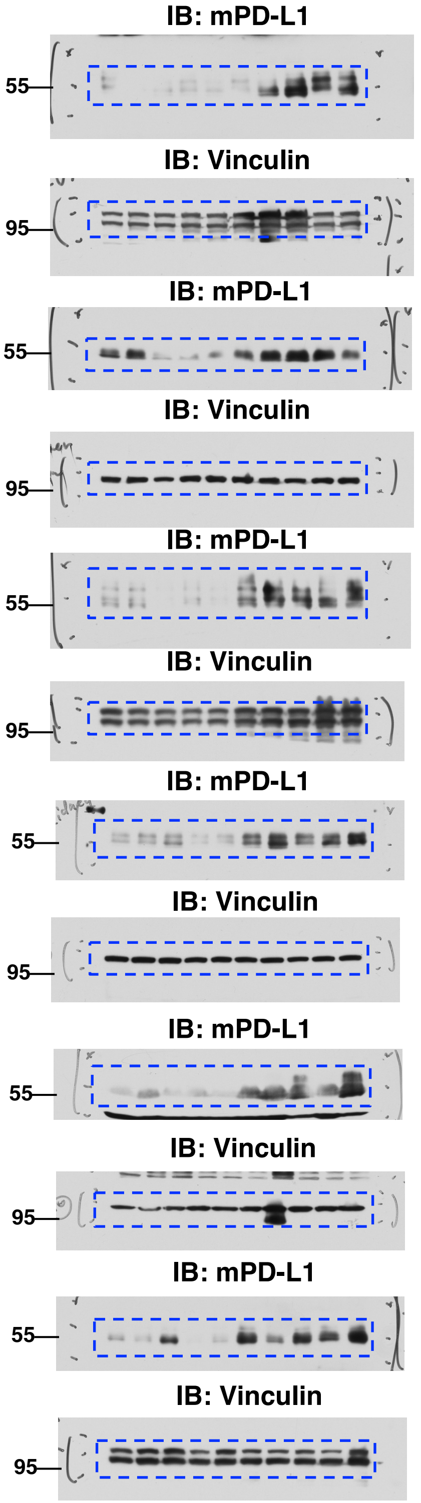

Figure 6h

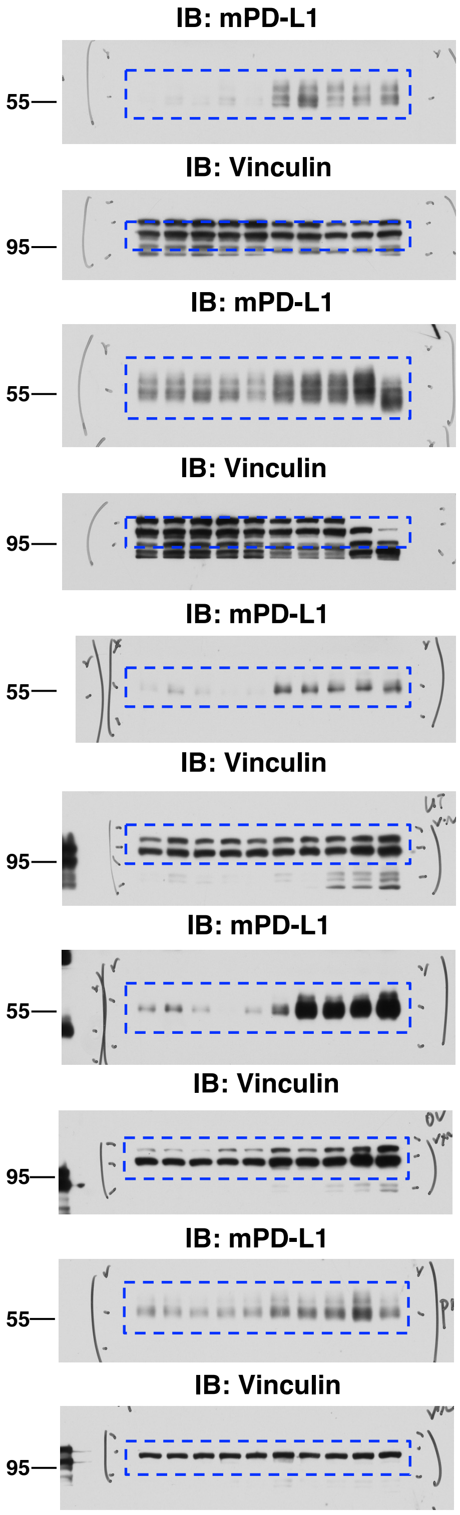

Figure 6k

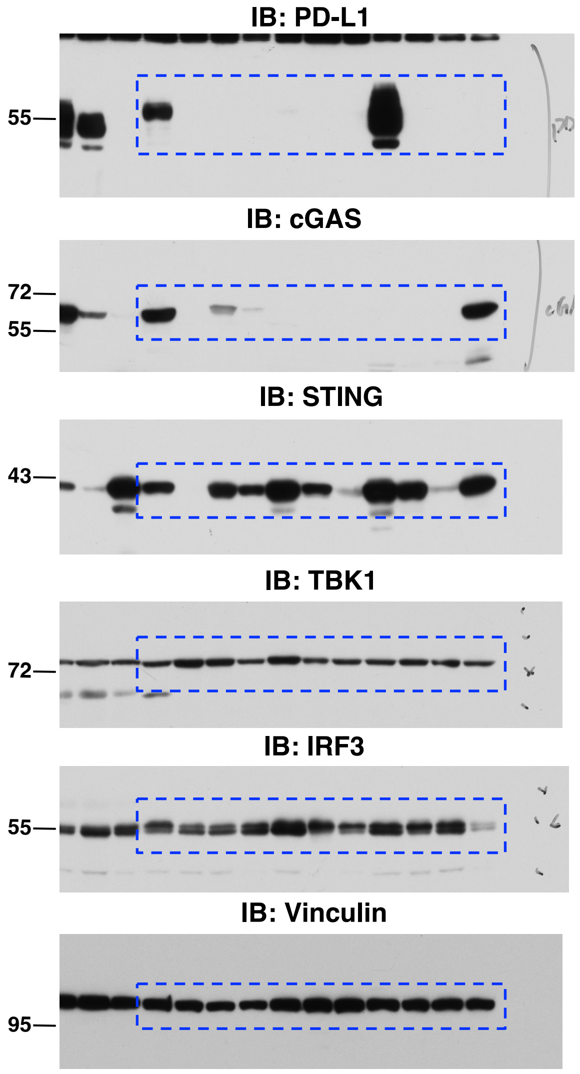

Figure 6l

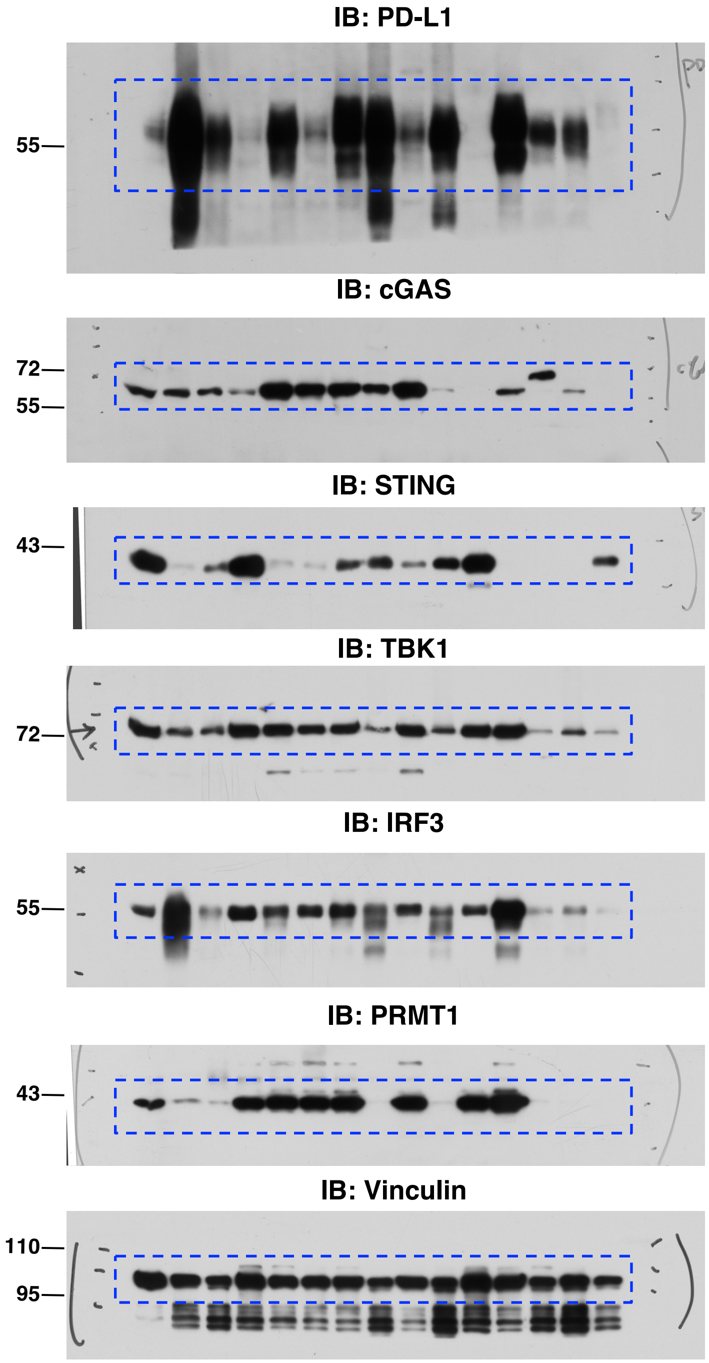

Figure 6m

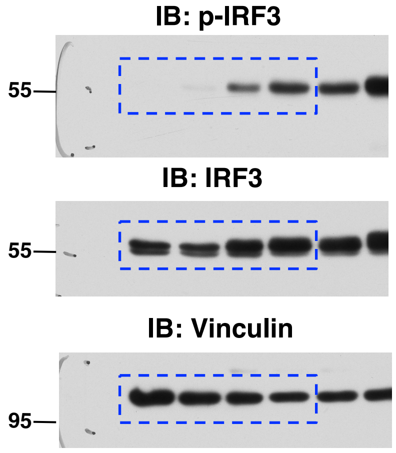

Figure 6n

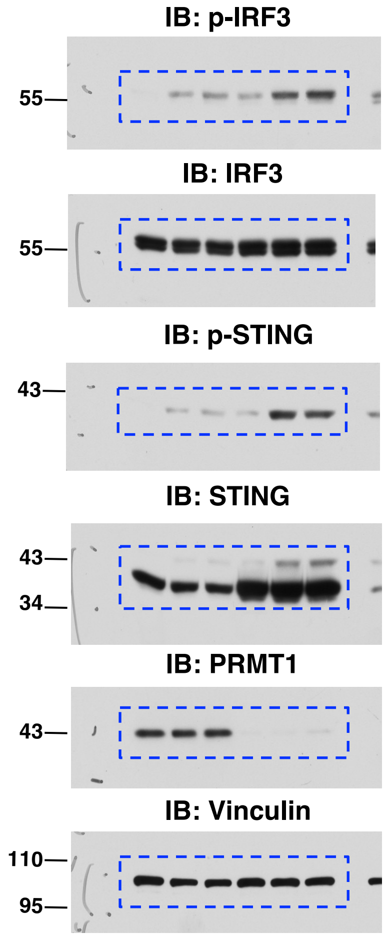

Gating strategy for Fig. 5k, l, and o

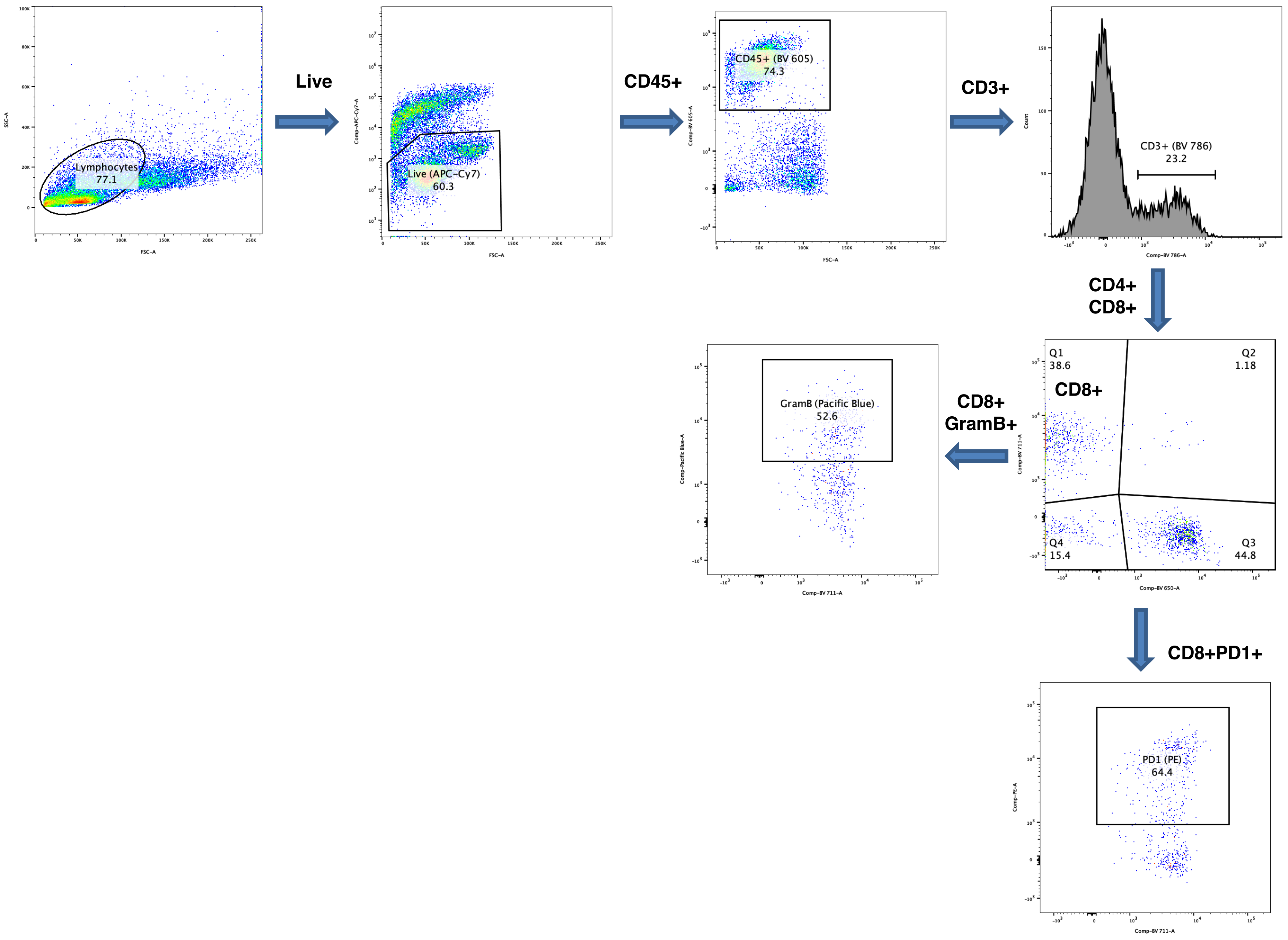

Gating strategy for Fig. 5m and n.

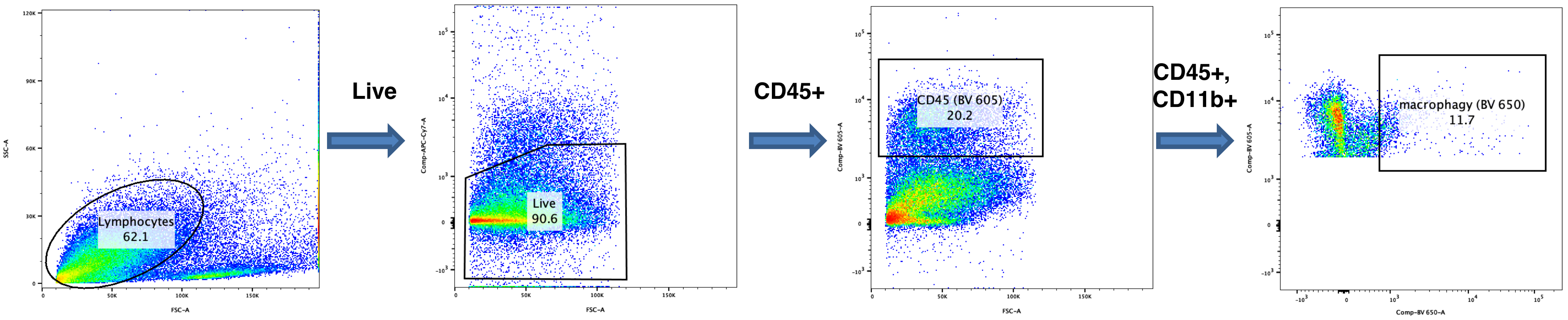

Supplement: Supplementary file 3 — Source Data [file 41467_2023_38443_MOESM3_ESM.zip › Source data-uncropped data.pdf]
